# Genetic effects on gene expression across human tissues

**DOI:** 10.1038/nature24277

**Published:** 2017-10-11

**Authors:** GTEx consortium

## Abstract

Characterization of the molecular function of the human genome and its variation across individuals is essential for identifying the cellular mechanisms that underlie human genetic traits and diseases. The Genotype-Tissue Expression (GTEx) project aims to characterize variation in gene expression levels across individuals and diverse tissues of the human body, many of which are not easily accessible. Here we describe genetic effects on gene expression levels across 44 human tissues. We find that local genetic variation affects gene expression levels for the majority of genes, and we further identify inter-chromosomal genetic effects for 93 genes and 112 loci. On the basis of the identified genetic effects, we characterize patterns of tissue specificity, compare local and distal effects, and evaluate the functional properties of the genetic effects. We also demonstrate that multi-tissue, multi-individual data can be used to identify genes and pathways affected by human disease-associated variation, enabling a mechanistic interpretation of gene regulation and the genetic basis of disease.

The human genome encodes instructions for the regulation of gene expression, which varies both across cell types and across individuals. Recent large-scale studies have characterized the regulatory function of the genome across a diverse array of cell types, each from a small number of samples^1–3^. Measuring how gene regulation and expression vary across individuals has further expanded our understanding of the functions of healthy tissues and the molecular origins of complex traits and diseases^4–9^. However, these studies have been conducted in limited, accessible cell types, thus restricting the utility of these studies in informing regulatory biology and human health.

The Genotype-Tissue Expression (GTEx) project was established to characterize human transcriptomes within and across individuals for a wide variety of primary tissues and cell types. Here, we report on a major expansion of the GTEx project that includes publicly available genotype, gene expression, histological and clinical data for 449 human donors across 44 (42 distinct) tissues. This enables the study of tissue-specific gene expression and the identification of genetic associations with gene expression levels (expression quantitative trait loci, or eQTLs) across many tissues, including both local (*cis*-eQTLs) and distal (*trans*-eQTLs) effects.

In this study, we associate genetic variants with gene expression levels from the GTEx v6p release. We found pervasive *cis*-eQTLs, which affect the majority of human genes. In addition, we identify *trans*-eQTLs across 18 tissues and highlight their increased tissue specificity relative to *cis*-eQTLs. We evaluate both *cis*- and *trans*-eQTLs with respect to their functional characteristics, genomic context, and relationship to disease-associated variation.

## Study design

**Figure 1:**
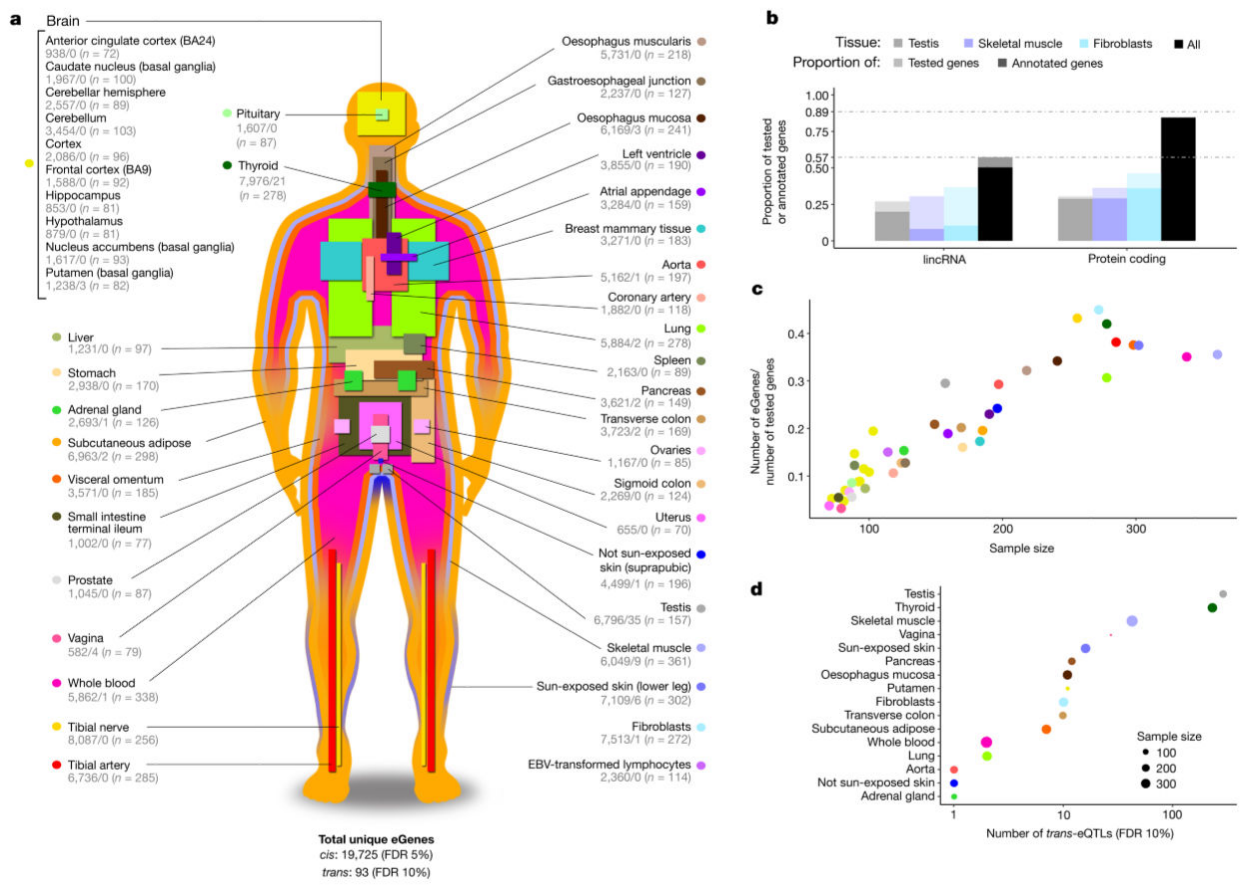
Sample size and eGene discovery in the GTEx v6p study **a**, Illustration of the 44 tissues and cell lines included in the GTEx v6p project with the associated number of *cis*- (left) and *trans*-eGenes (right) and sample sizes. Each tissue has a unique colour code (defined in Supplementary Fig. 5). **b**, Fraction of genes that are eGenes across all tissues by transcript class. The three tissues highlighted are: testis, which has the highest proportion of *trans*-eGenes; skeletal muscle, which has the largest sample size; and fibroblasts, which have the highest proportion of *cis*-eGenes. Dark bars depict the fraction of all curated human genes in GENCODE v19. Light bars depict the fraction of genes expressed in one or more tissues. **c**, Proportion of expressed genes that are *cis*-eGenes (*y*-axis) as a function of tissue sample size (*x*-axis). Colours represent tissues, as in **a. d**, Number of *trans*-eQTLs (*x*-axis) per tissue (*y*-axis), with sample size indicated by point size.

The GTEx project has created a reference resource of gene expression levels from ‘normal’, non-diseased tissues. Every tissue sample was examined histologically; the sample was accepted for the project if the tissue was non-diseased and in the normal range for the age of the donor. RNA was isolated from postmortem samples in an ongoing manner as donors were enrolled into the study. For this data release, 44 sampled regions or cell lines were considered, each from at least 70 donors, and thereby considered suitable for eQTL analysis: 31 solid- organ tissues, 10 brain subregions including duplicates of two regions (cortex and cerebellum), whole blood, and two cell lines derived from donor blood and skin samples. We hereafter refer to these tissues, regions, and cell lines as the ‘tissues’ used in eQTL analysis. A total of 7,051 samples from 449 donors represent the GTEx v6p analysis freeze (Fig. 1a; Supplementary Information 1–5; Supplementary Figs 1–6; Supplementary Tables 1–10). RNA sequencing (RNA-seq) samples were sequenced to a median depth of 78 million reads. This is 4.3 times more samples than reported in the GTEx pilot phase^10^. DNA was genotyped at 2.2 million sites and imputed to 12.5 million sites (11.5 million autosomal and 1 million X chromosome sites) using the multi-ethnic reference panel from 1000 Genomes Project Phase 1 v3^11^. Sampled donors were 83.7% European American and 15.1% African American. Whole-genome sequencing was performed for 148 donors to a mean coverage greater than 30×, and all donors were exome-sequenced to a mean coverage over captured exons of 80×. The resulting data provide the deepest survey of individual- and tissue- specific gene expression to date, enabling a comprehensive view of the impact of genetic variation on gene expression levels. All data are available from dbGaP (accession phs000424.v6.p1) with multiple data views publicly available from the GTEx Portal (www.gtexportal.org).

## Expression QTLs across human tissues

**Extended Data Figure 1:**
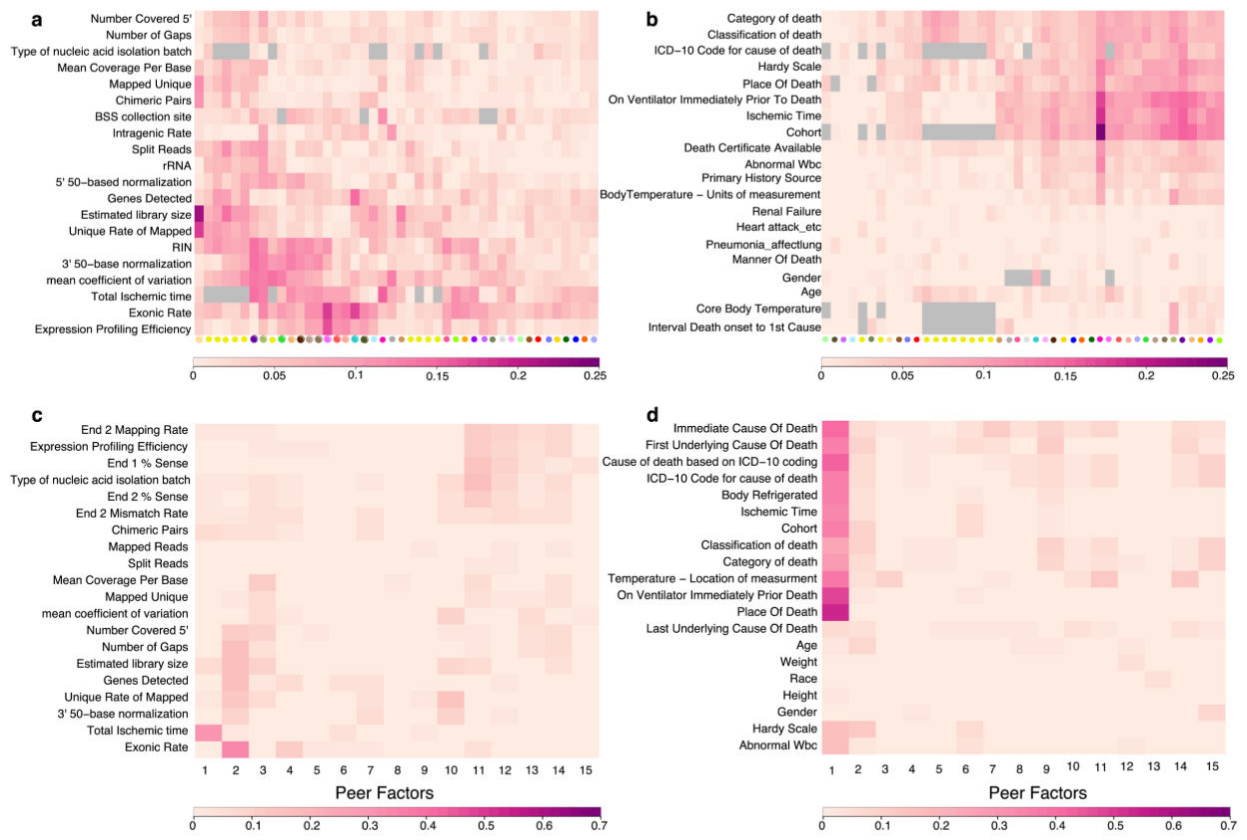
Association of known covariates with expression components removed by PEER Each cell depicts the adjusted (*R*^2^) between a pair of variables. Scale bar specific to each panel is displayed at the bottom. Grey cells represent pairs of variables without sufficient data to estimate correlation. **a**, For each tissue, adjusted (*R*^2^) reflecting the proportion of variance explained by each covariate of the entire PEER component removed from the expression data. A selected set of the most relevant sample-specific covariates is shown here. **b**, For each tissue, adjusted (*R*^2^) reflecting the proportion of variance explained by each covariate of the entire PEER component removed from the expression data. A selected set of the most relevant donor-specific covariates is shown here. See Supplementary Information for complete set of covariates. **c**, Adjusted *R*^2^ between each PEER factor and known sample covariates in skeletal muscle. **d**, Adjusted *R*^2^ between each PEER factor and known donor covariates in skeletal muscle.

**Extended Data Figure 2:**
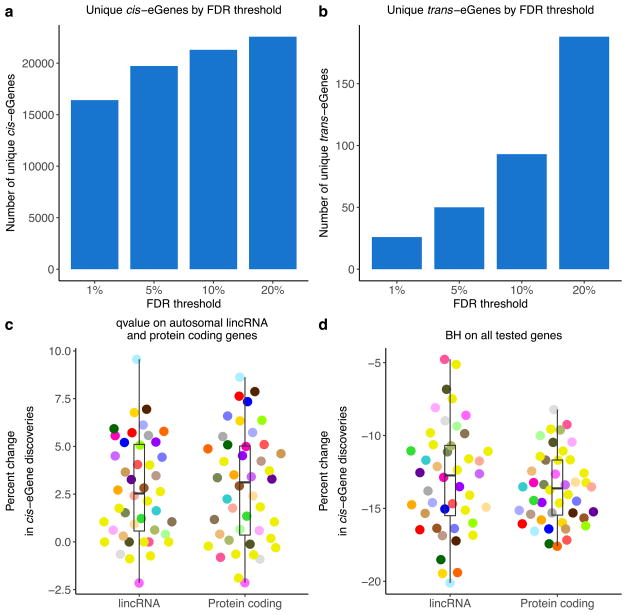
Controlling the discovery of *cis*- and *trans*-eGenes **a**, **b**, The number of unique *cis*- (**a**) and *trans*-eGenes (**b**) across all tissues at varying FDR thresholds. **c**, The per cent change in the number of *cis*-eGene discoveries comparing FDR (Storey’s *q* value) calculated across all tested genes in each tissue to FDR calculated only across autosomal lincRNA and protein-coding genes. Results are shown for each tissue and are stratified by gene type. The per cent change for each tissue and gene type is calculated as 100 ×(no. of *cis*-eGenes from *q* value on the restricted gene set – no. of *cis*-eGenes from *q* value on all tested genes)/(no. of *cis*-eGenes from *q* value on all tested genes). **d**, The per cent change in the number of *cis*-eGene discoveries comparing *q* value and the Benjamini–Hochberg (BH) procedure applied to all tested genes in each tissue. The percent change for each tissue and gene type is calculated as 100 ×(no. of *cis*-eGenes from BH – no. of *cis*-eGenes from *q* value)/(no. of *cis*-eGenes from *q* value). Box plots depict the IQR, whiskers depict 1.5× IQR.

**Extended Data Figure 3:**
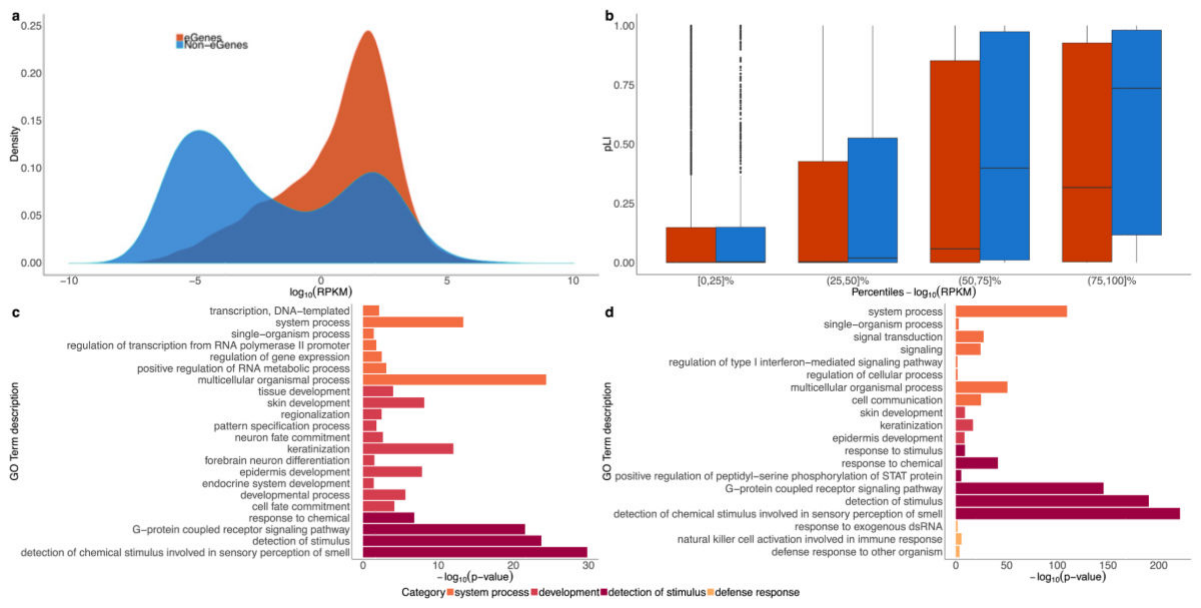
Properties of *cis*-eGenes and non-eGenes In these analyses, ‘non-eGenes’ refers to the set of genes with no significantly associated *cis*-eQTLs. **a**, Density of expression (mean across samples, median across tissues, in log_10_ scale) for *cis*-eGenes and non-eGenes. The difference in mean expression for *cis*-eGenes and non-eGenes is significant (Wald test; *P* <1 ×10^−16^). **b**, Box plots comparing the probability of being loss-of-function-intolerant (pLI) scores^41^ for *cis*-eGenes and non-eGenes, stratified by expression percentile across all genes (mean across samples, median across tissues, in log_10_ scale). On average, highly expressed non-eGenes have higher pLI scores than highly expressed *cis*-eGenes (*t*-test for the difference in mean pLI score between *cis*-eGenes and non-eGenes; BH adjusted *P* = 8.3 ×10^−4^ and 2.1 ×10^−9^ for (50,75]% and (75,100]%, respectively) and lowly expressed non-eGenes (*t*-test for the difference in mean pLI score between highly and lowly expressed non-eGenes; BH adjusted *P* ≤7.8 ×10^−46^, *P* ≤5.5 ×10^−17^ and *P* ≤2.1 ×10^−3^ for (75,100]% vs [0,25]%, (75,100 vs (25,50]% and (75,100]% vs (50,75]%, respectively). **c**, Gene Ontology (GO) analysis of tested protein-coding non-eGenes. We used the PANTHER overrepresentation test^78^ (release 20160715) against 20,972 human genes as background to test for enrichment in GO biological processes using the GO database release 2017-02-28. Significant GO IDs (Bonferroni adjusted *P* < 0.05) were selected for analysis with REVIGO^79^ to group similar ontological terms, which yielded 22 over-represented GO IDs. **d**, GO analysis of a more stringent set of protein-coding non-eGenes. Selected genes included those not tested in GTEx (532 genes) or those with a minimum nominal *P* value across tissues greater than 0.1 (692 genes). Of these stringent 1,224 non-eGenes, 808 were mapped in the GO analysis. Using a similar approach as in **c**, 20 over-represented GO IDs were identified. For both **c** and **d**, the *x*-axis represents the −log_10_ P value resulting from GO analysis. GO IDs are coloured by the broader enrichment category to which each corresponds. Box plots depict the IQR, whiskers depict 1.5× IQR.

We identified associations between the expression levels of all expressed genes (eGenes) and genetic variants (eVariants) located within 1 Mb of the target gene’s transcription start site (TSS), which we refer to as *cis*-eQTLs for convenience, without requiring evidence of allelic effects at each locus. However, the majority of *cis*-eQTLs do exhibit allele specific expression. We applied a linear model controlling for ancestry, sex, genotyping platform and latent factors^12^ in the expression data for each tissue that may reflect batch or other technical variables (see Methods; Extended Data Fig. 1, Supplementary Information 6 and Supplementary Figs 7–10). Considering all tissues, we found a total of 152,869 *cis*-eQTLs for 19,725 genes, representing 50.3% and 86.1% of all known autosomal long intergenic noncoding RNA (lincRNA) and protein-coding genes, respectively (Fig. 1a, b). We identified a median of 2,816 autosomal protein-coding or lincRNA eGenes at a 5% false discovery rate (FDR) within each tissue (Extended Data Fig. 2a). Protein-coding genes without a *cis*-eQTL in any tissue were more likely to be expressed at low levels or loss-of-function intolerant and were enriched for functions related to development and environmental response, indicating specific contexts in which additional eQTLs may be identified (Extended Data Fig. 3). To identify *cis*-eGenes affected by more than one functional regulatory variant, we applied forward–backward stepwise regression (see Methods). This approach identified an additional 24,886 secondary *cis*-eQTLs, with 41.2% of protein-coding genes and 24.8% of lincRNAs having multiple, conditionally independent eVariants in at least one tissue (Supplementary Fig. 11).

**Extended Data Figure 4:**
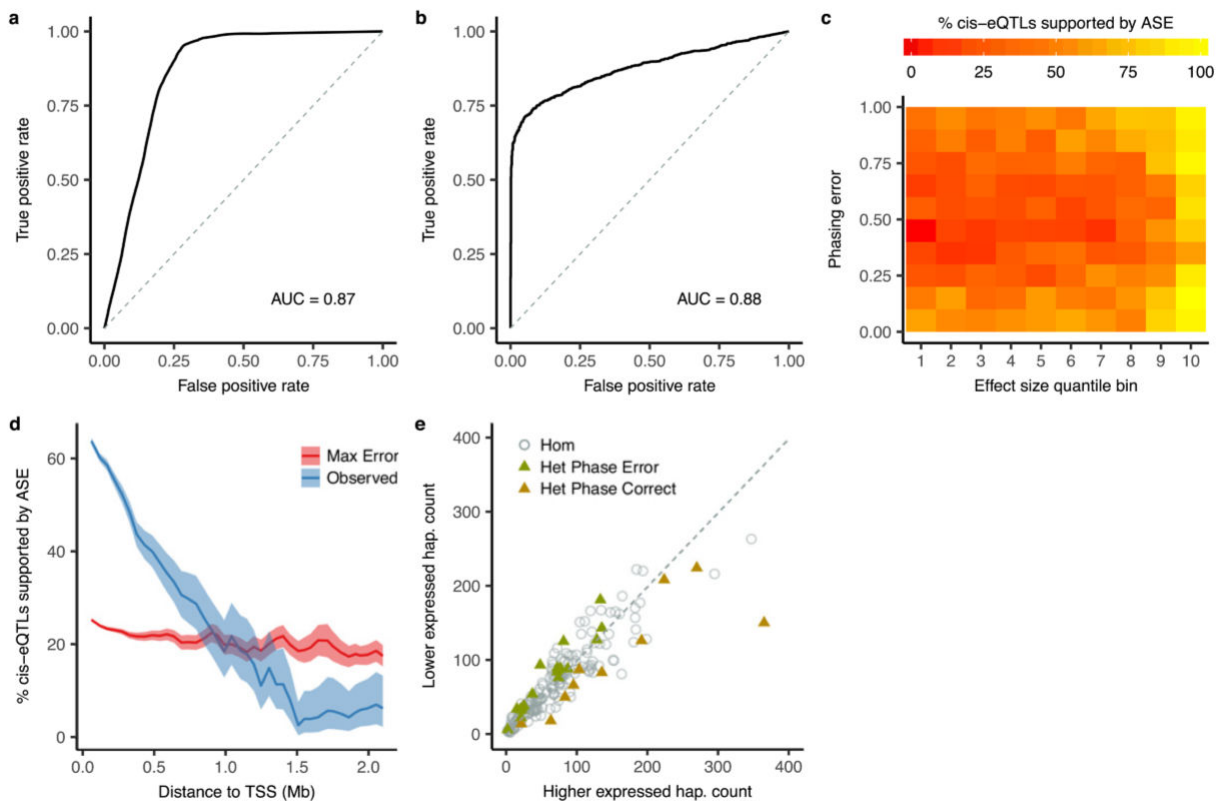
Identification of *cis*-acting eQTLs using allele-specific expression at chromosome-wide distances **a**, A logistic regression based model was developed to predict the probability of phasing error as a function of distance and variant minor allele frequencies. When applied to chromosome 2 of 1000 Genomes sample NA12878, this model had a receiver operating characteristic (ROC) area under the curve (AUC) of 0.87 using population phasing compared to transmission phasing. **b**, ROC when applying the beta-binomial mixture model to detect *cis*-acting regulation to the GTEx v6p subcutaneous adipose *cis*-eQTLs, with an AUC of 0.88. As the null, eGenes were shuffled with respect to eVariants. **c**, Power analysis using all nominally significant (*P* <1.0 ×10^−5^) linkage disequilibrium pruned associations within 100 kb of the TSS illustrating the number of eQTLs with nominally significant (*P* ≤ 0.01) evidence of *cis*-regulation as a function of phasing error and eQTL effect size. Expression QTL effect size was calculated using a companion method^28^, and uniform phasing error between 0 and 100% was introduced *in silico*. **d**, Proportion of nominally significant (*P* <1.0 ×10^−5^) linkage disequilibrium-pruned intrachromosomal eQTLs with nominally significant (*P* ≤ 0.01) ASE supported evidence of *cis* regulation in bins of increasing TSS distance. Observed indicates what is seen in the data, while Max Error indicates what would be expected in the worst-case scenario of phasing error (50%). **e**, Example of significant ASE supported *cis*-regulation at a distance of 52.7 Mb between eVariant rs17494053 and eGene ENSG00000108509 in whole blood. Each point represents allelic imbalance in a single eVariant homozygote (circle) or heterozygote (triangle).

To identify *trans*-eQTLs, we tested for association between every protein-coding or lincRNA gene and all autosomal variants where the gene and variant were on different chromosomes. To minimize false positives in *trans*-eQTL detection, we controlled for the same observed and inferred confounders as in the *cis*-eQTL analysis, and further removed genes with poor mappability, variants in repetitive regions, and *trans*-eQTLs between pairs of genomic loci with evidence of RNA-seq read cross-mapping due to sequence similarity. Applying this approach, we identified 673 *trans*-eQTLs at a 10% genome-wide FDR. This includes 112 distinct loci (*R*^2^ ≤ 0.2) and 93 unique genes (94 total gene associations, including a *trans*-eGene detected in both testis and thyroid) in 16 tissues (Extended Data Table 1, Extended Data Fig. 2b, Supplementary Information 7, 8, Supplementary Figs 12–15 and Supplementary Table 11). An alternative approach to quantifying FDR at the gene level (Supplementary Information 8 and Supplementary Table 12) identified 46 genes at 10% FDR, with estimated *q* values of less than 0.4 for all 94 gene associations identified using the genome-wide FDR^16^. By investigating long-range intra-chromosomal eQTLs (≥5 Mb from the TSS), we discovered an additional 33 eGenes (10% FDR; Extended Data Table 2 and Supplementary Information 9). We found decaying support for *cis*-regulation (or interaction between *cis*- and *trans*-effects) over increased genomic distances based on evidence of allelic effects (Extended Data Fig. 4). Evidence of *cis*-regulation fell below background levels between 0.85 and 1.3 Mb from the TSS, empirically supporting the conventional distance threshold of 1 Mb for *cis*-eQTL detection.

**Extended Data Figure 5:**
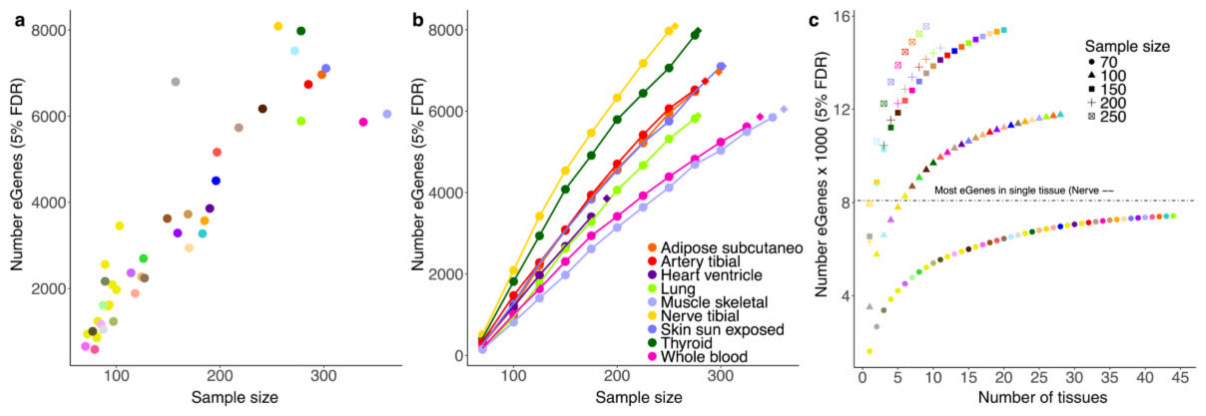
Effects of sample size and assayed tissues on *cis*-eGene discovery **a**, Number of significant *cis*-eGenes at 5% FDR (*y*-axis) discovered in 44 GTEx tissues versus sample size (*x*-axis). **b**, Number of significant eGenes at FDR 5% (*y*-axis) discovered in nine GTEx tissues each subsampled to various sizes where possible (*n* =70, 100, 125, 150, 175, 200, 225, 250, 275, 300, 325, and 350; *x*-axis). We computed the number of *cis*-eGenes at each subsample size (circles connected by lines). We also plotted the number of *cis*-eGenes discovered with no subsampling of donors (diamonds). **c**, Number of significant *cis*-eGenes at FDR 5% (*y*-axis) as a function of sample size and number of tissues assayed (*x*-axis). Each tissue was subsampled to 70, 100, 150, 200, and 250 donors, and a forward search was used to assess sequential combinations of tissues that maximize the total number of unique *cis*-eGenes discovered.

As expected, sample size greatly affects eQTL mapping. Discovery of eGenes increased with sample size with no evidence of saturation at the full sample size for each tissue (Fig. 1c). The tissue with the highest number of identified *cis*-eGenes was tibial nerve, with 8,087 eGenes in 256 samples. Testis had the most *trans*-eGenes, with 35 eGenes in 157 samples (Fig. 1d), consistent with the elevated number of expressed genes (16,853 protein-coding genes and 4,362 lincRNA genes) and *cis*-eGenes (6,796 genes). Continued discovery of eGenes with increasing sample size suggests that the expression of nearly all genes is influenced by genetic variation (Extended Data Fig. 5a, b). We further observed that, for sub-sampled data ranging from 70 to 250 donors, sample size was a more significant contributor than additional tissues to the discovery of novel *cis*-eGenes (Extended Data Fig. 5c). For *trans*-eQTL mapping, we used informed subsets of variants to reduce the number of tests by one to three orders of magnitude (Supplementary Information 10 and Supplementary Table 13). We found statistical power to detect additional associations in these restricted tests, such as the test restricted to *cis*-eVariants. Our results indicate that ongoing increases in sample size will continue to yield additional eQTLs, both in the *cis*-eQTL setting, where smaller and conditionally independent effects will be identified, and in the *trans*-eQTL setting, where statistical power is the main limitation.

## Allele-specific expression across human tissues

**Extended Data Figure 6:**
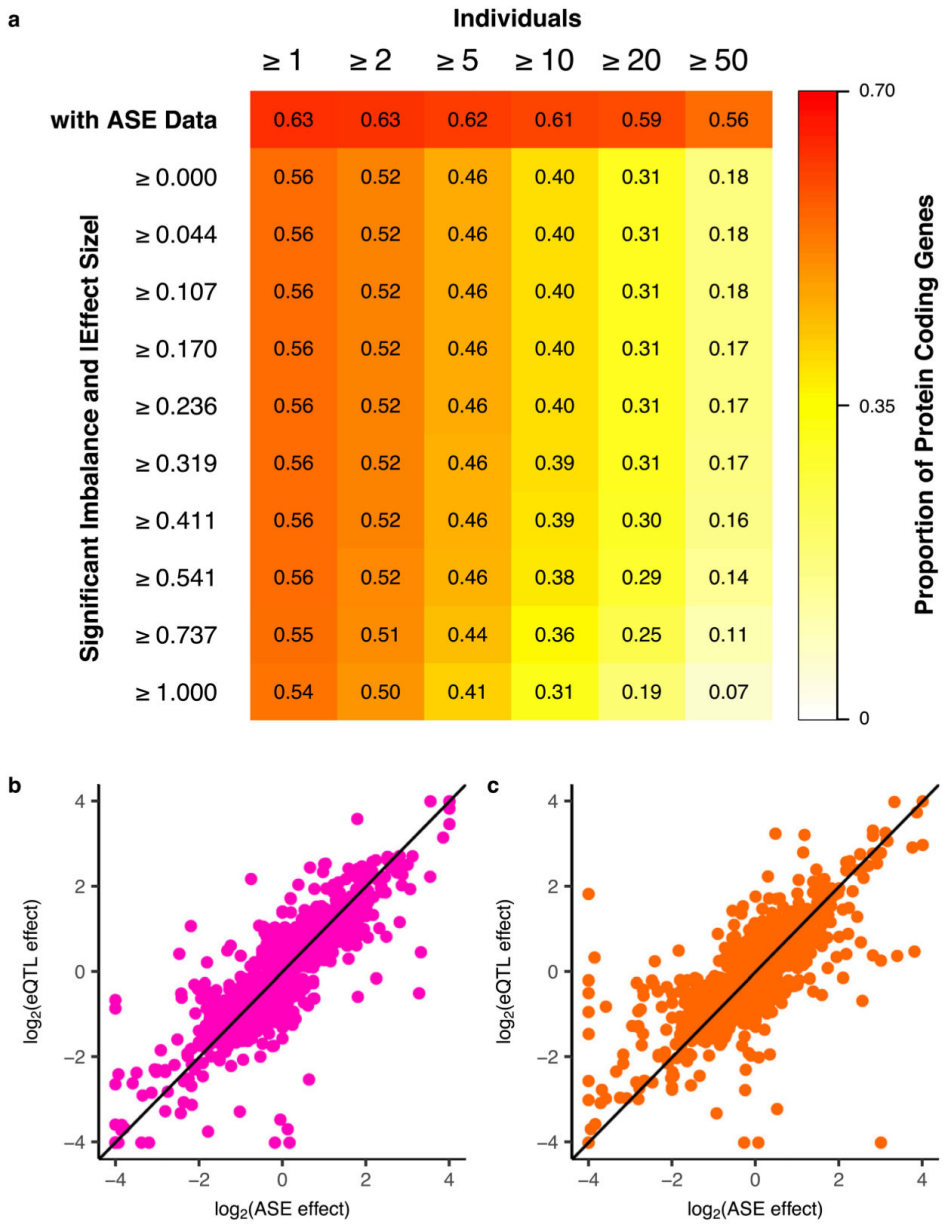
Measuring *cis*-regulatory variation using ASE **a**, Proportion of protein-coding genes with ASE data in at least one tissue as a function of donors (top row) and with significant imbalance (binomial test versus 0.5, 5% FDR) stratified by ASE effect size (|log_2_(hap*_a_* count/ hap*_b_* count)|) deciles. Gene-level measurements of haplotype expression were calculated by aggregating counts per sample across all heterozygous variants with ASE data within the gene using population phasing. The following filters were applied on ASE data: total coverage ≥8 reads, no mapping bias in simulations^76^, UCSC mappability > 50, and no significant (FDR > 1%) evidence that variant is monoallelic in expression data^75^. **b**, log_2_ transformed *cis*-eQTL effect size (*x*-axis) versus log_2_ transformed ASE effect size (*y*-axis) for whole blood (Spearman’s *ρ* =0.82) and **c**, subcutaneous adipose (Spearman’s *ρ* =0.74).

The effect of *cis*-regulatory variation can also be quantified by allele- specific expression (ASE) analyses obtained by measuring the allelic imbalance of RNA-seq reads at transcribed heterozygous sites. A large-scale, multi-tissue resource of ASE estimates complements eQTL mapping by providing access to individual-specific effects, which assists in the interpretation of rare variants, somatic mutations and patterns of imprinting^8,13,14^. We measured ASE at more than 135 million sites across tissues and donors, with a median of over 10,000 genes quantified per donor (Supplementary Information 11 and Supplementary Fig. 16). In total, 63% of all protein-coding genes could be tested for ASE in at least one donor and tissue, with 54% exhibiting significant allelic imbalance (binomial test, 5% FDR, |effect size| ≥ 1, Extended Data Fig. 6a). Overall, 88% of testable genes had significant allelic imbalance in at least one donor (binomial test, 5% FDR), demonstrating an abundance of *cis*-linked regulatory effects. Per donor, a median of 1,963 genes had significant allelic imbalance in at least one tissue, with a median of 570 genes where the donor was not heterozygous for a top eVariant, suggesting more complex or rarer regulatory effects at these loci.

## Tissue-sharing and specificity of eQTLs

**Figure 2:**
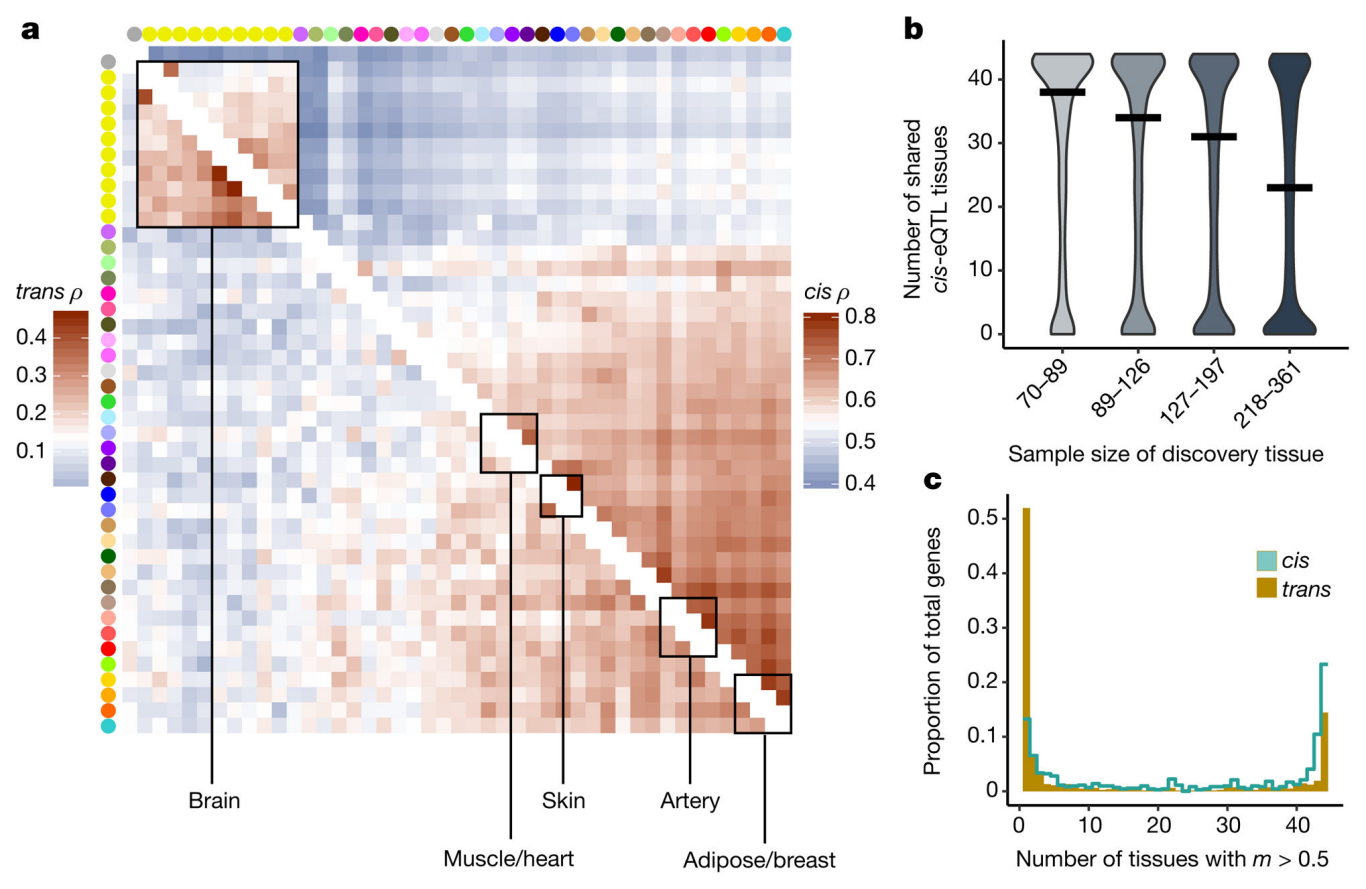
Patterns of tissue sharing of eQTL effects **a**, Similarity (Spearman’s *ρ*) of Meta-Tissue effect sizes between tissues for *cis*- (upper triangle, 5% FDR) and *trans*- (lower triangle, 50% FDR) eQTLs. Tissues (by colours as in Fig. 1a) are ordered by agglomerative hierarchical clustering of the *cis*-eQTL results. **b**, The number of tissues in which a given eQTL is shared as a function of tissue sample size. For each tissue, we estimated the degree of sharing (number of tissues with *m* >0.9) for all eQTLs identified in that tissue at a 5% FDR. Tissues were then binned into quartiles on the basis of sample size. A higher proportion of eQTLs identified in tissues with small sample sizes have shared effects across multiple tissues compared with more deeply sampled tissues. This pattern inverts at higher sample sizes where more of the effects are tissue-specific. The median number of shared tissues is plotted for each quartile as a horizontal black line. **c**, Distribution of the number of tissues having Meta-Tissue *m* > 0.5 for the top variant for each *trans*-eGene at 50% FDR, and FDR-matched, randomly selected *cis*-eGenes (also 50% FDR). *cis*-eGenes were matched for discovery tissue to the *trans*-eGenes.

The extensive and diverse tissue sampling allowed us to develop a global view of how genetic effects vary between tissues of the human body by evaluating the sharing of eQTLs across tissues. We performed a meta-analysis across all 44 tissues for both *cis*- and *trans*-eQTLs to assess eQTL sharing between tissues. To do so, we applied Meta-Tissue^15^, a linear mixed model that allows for heterogeneity in effect sizes across tissues and controls for correlated expression measurements that result from collecting multiple tissues from the same donors. For each eQTL, we estimated the posterior probability that the effect is shared in each tissue (*m* value). For both *cis*- and *trans*-eQTLs, we observed patterns that reflected relationships between related tissues and concordance between *cis* and *trans* in estimates of tissue similarity (Fig. 2a, Supplementary Information 12 and Supplementary Fig. 17). The strongest broad pattern observed was the high correlation among brain tissues (median Spearman’s *ρ* of 0.584 (*cis*) and 0.241 (*trans*)) and among non-brain tissues (median Spearman’s *ρ* of 0.606 (*cis*) and 0.165 (*trans*)), with much lower correlation observed between these two groups (median Spearman’s *ρ* of 0.499 (*cis*) and 0.096 (*trans*)). Within non-brain tissues, we observed strong correlation among closely related tissues, such as arterial tissues (median Spearman’s *ρ* of 0.743 (*cis*) and 0.264 (*trans*)), skeletal muscle and heart tissues (median Spearman’s *ρ* of 0.672 (*cis*) and 0.184 (*trans*)), and skin tissues (Spearman’s *ρ* of 0.804 (*cis*) and 0.365 (*trans*)). Overall, the median pairwise correlation between tissues was 0.547 (*cis*) and 0.138 (*trans*).

**Extended Data Figure 7:**
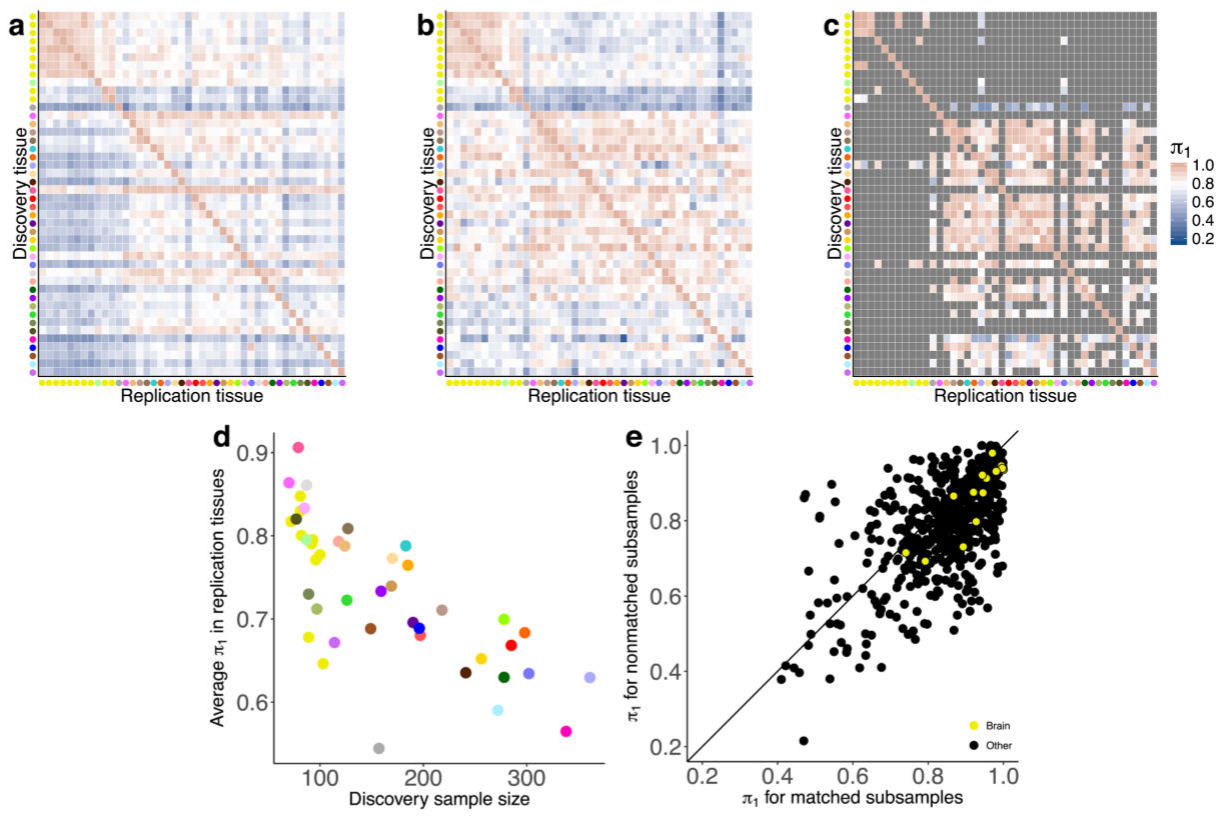
Replication of *cis*-eQTLs between tissues **a**, *π*_1_ statistics for *cis*-eQTLs are reported for all pairwise combinations of discovery (*y*-axis) and replication (*x*-axis) tissues. Higher *π*_1_ values indicate a stronger replication signal. Tissues are grouped using hierarchical clustering on rows and columns separately with a distance metric of 1 − *ρ*, where *ρ* is the Spearman’s correlation coefficient of *π*_1_ values. *π*_1_ is calculated only when the gene is expressed and testable in the replication tissue. **b**, **c**, *π*_1_ replication is reported between tissues subsampled down to 70 non-matched (**b**) and matched (**c**) donors. In **(c)**, grey tiles indicate tissue pairs with fewer than 70 shared donors. **d**, Effect of sample size (*x*-axis) on average *π*_1_ replication (*y*-axis) across all replication tissues. **e**, Scatter plot of *π*_1_ scores among tissue pairs with matched (*x*-axis) and non-matched (*y*-axis) donors.

**Extended Data Figure 8:**
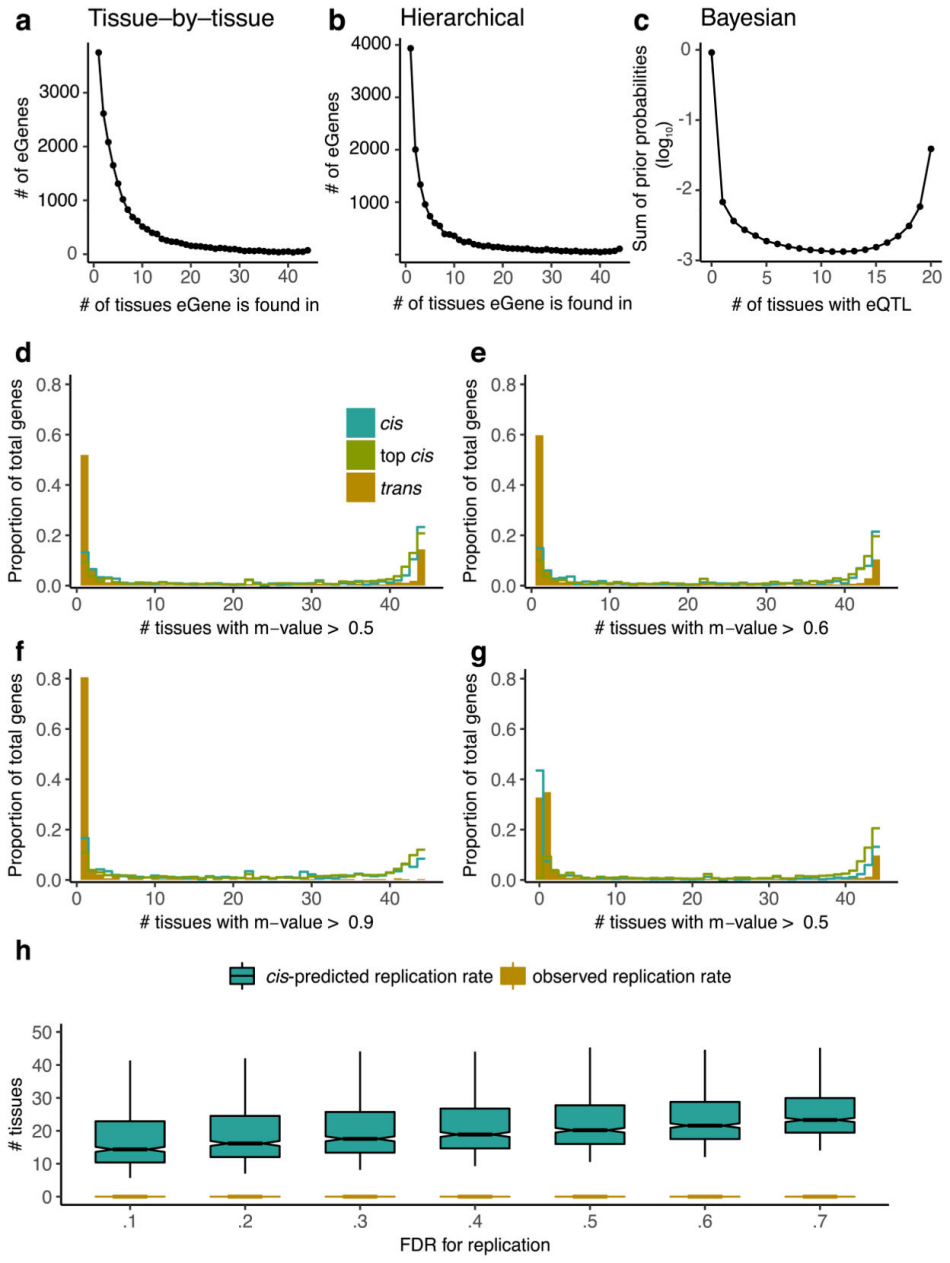
Tissue-specificity of *cis*- and *trans*-eQTLs **a**, Sharing of independently identified *cis*-eGenes across the 44 GTEx tissues (*cis*-eGenes are independently identified in each of the 44 tissues and then binned by the number of tissues in which they appear). **b**, Sharing of *cis*-eGenes across 44 GTEx tissues that were identified using the hierarchical multi-tissue analysis. **c**, The prior probabilities of having significant variant–gene association in different numbers of tissues, calculated using an empirical Bayes model. The prior probabilities are summed up on the basis of the Hamming weights of the corresponding *cis*-eQTL configurations. **d**–**g**, Meta-analysis performed using Meta-Tissue for *trans*-eGenes (50% FDR), randomly selected *cis*-eGenes (50 % FDR), and an equal number of the top *cis*-eGenes by *P* value. Distribution of the number of tissues that have Meta-Tissue *m* values greater than a given threshold (**d**, 0.5; **e**, 0.6; **f**, 0.9) across variant–gene pairs that have an effect (based on *m* value thresholding) in at least one tissue. **g**, The same distribution as **d** except that variant–gene pairs with predicted effect in zero tissues (based on the number of *m* values > 0.5) are included. Meta-Tissue predicts that many *cis*-eGenes (50% FDR) and *trans*-eGenes (50% FDR) will have an effect in zero tissues. The number of zero predictions is largely reduced for the top most significant *cis*-eGenes. **h**, Distribution of observed replication rate between pairs of tissues for *trans*-eQTLs (10% FDR) versus the predicted replication rate for *trans*-eQTLs (10% FDR) based on a negative binomial generalized linear model trained on *cis*-eQTLs (10% FDR0.1). This model directly accounts for effect size and minor allele frequency. Replication rates shown for a range of FDR thresholds in replication tissue. Box plots depict the IQR, whiskers depict 1.5× IQR.

The patterns of sharing were also supported by replication between single-tissue *cis*-eQTLs, estimated by *π*_1_ (the proportion of true positives^16^) among the eQTLs identified in one tissue and then tested for replication in a second tissue (Extended Data Fig. 7a, median *π*_1_ = 0.740). The patterns held even when accounting for variable number of overlapping donors among pairs of tissues in the GTEx study design (Extended Data Fig. 7b–e). *cis*-eQTLs exhibited a distinctly bimodal pattern of tissue sharing—they were likely to be either shared across most of the 44 tissues or specific to a small subset of tissues (Fig. 2b). This bimodality was further supported by three different methods: simple overlap of the single tissue eQTLs, a hierarchical multiple comparison procedure (treeQTL^17^), and an empirical Bayes model (MT-eQTL^18^; Extended Data Fig. 8a–c). Each method also demonstrated that *cis*-eQTLs discovered in tissues with larger sample sizes were more often tissue-specific; however, estimates of tissue-specificity for large sample-size tissues can be influenced by difficulty in replicating small effect-size eQTLs in tissues with fewer samples.

**Extended Data Figure 9:**
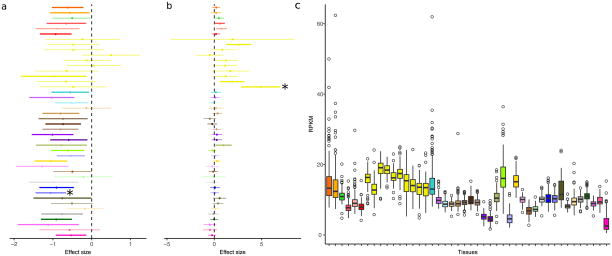
Examples of *trans*-eQTLs shared across tissues **a**, An example of a *trans*-eQTL (rs7683255–*NUDT13*) originally identified in sun-exposed skin (10%, *P* ≤ 1.1 ×10^−10^, indicated by asterisk) that has a global effect across tissues. The lines represent 95% confidence intervals of the effect sizes. A thicker line indicates that this variant–gene pair is called significant at *P* ≤ 0.05 in the corresponding tissue. **b**, An example of a *trans*-eQTL (rs60413914–*RMDN3*) that is genome-wide significant in putamen (basal ganglia) (10% FDR, *P* ≤1.2 ×10^−13^, indicated by asterisk) that has an effect in all five brain tissues tested but shows little effect in other tissues. **c**, Expression levels (RPKM) of *RMDN3* in all donors across 44 tissues. Box plots depict the IQR, whiskers depict 1.5× IQR.

Overall, we observed much greater tissue specificity for *trans*-eQTLs than a set of FDR-matched *cis*-eQTLs (Fig. 2c); this observation was robust to choices of *m* value threshold and selection criteria for matching *cis*-eQTLs (Extended Data Fig. 8d–g). While 3.8% of *trans*-eQTLs were shared across three or more tissues at *m* > 0.9, 25.3% of FDR-matched *cis*-eQTLs were shared. The extensive tissue-specificity of *trans*-eQTLs was also supported by a hierarchical approach for FDR control^17^, where we found no *trans*-eQTLs shared across more than one tissue (Extended Data Table 3). Our estimate of increased tissue specificity for *trans*-eQTLs agreed with the minimal sharing of *trans* effects reported in previous eQTL studies with fewer tissues^4,19^, and greatly exceeds what would be expected on the basis of replication between tissues for *cis*-eQTLs of matched minor allele frequency (MAF) and effect size (Wilcoxon rank sum test; *P* ≤ 2.2×10^−16^ for all choices of replication FDR; Extended Data Fig. 8h). Given the greater tissue-specificity of *trans*-eQTLs, we note that heterogeneity in cellular composition of bulk tissue samples is one important confounder that may reduce power to detect *trans*-eQTLs, or even lead to false positive associations^6^. Despite the high tissue-specificity, we did observe a small number of tissue-shared *trans*-eQTLs, including rs7683255, which was moderately associated in *trans* with *NUDT13* across most tested GTEx tissues with a consistent direction of effect (Extended Data Fig. 9a). We also found examples of *trans*-eQTLs shared across a subset of related tissues, such as an association between rs60413914 and *RMDN3*, a gene with increased expression levels in brain regions as compared to other tissue types, and for which the *trans*-eQTL had moderate effects in all tested brain regions but no strong effect in other tissues (Extended Data Fig. 9b, c).

**Extended Data Figure 10:**
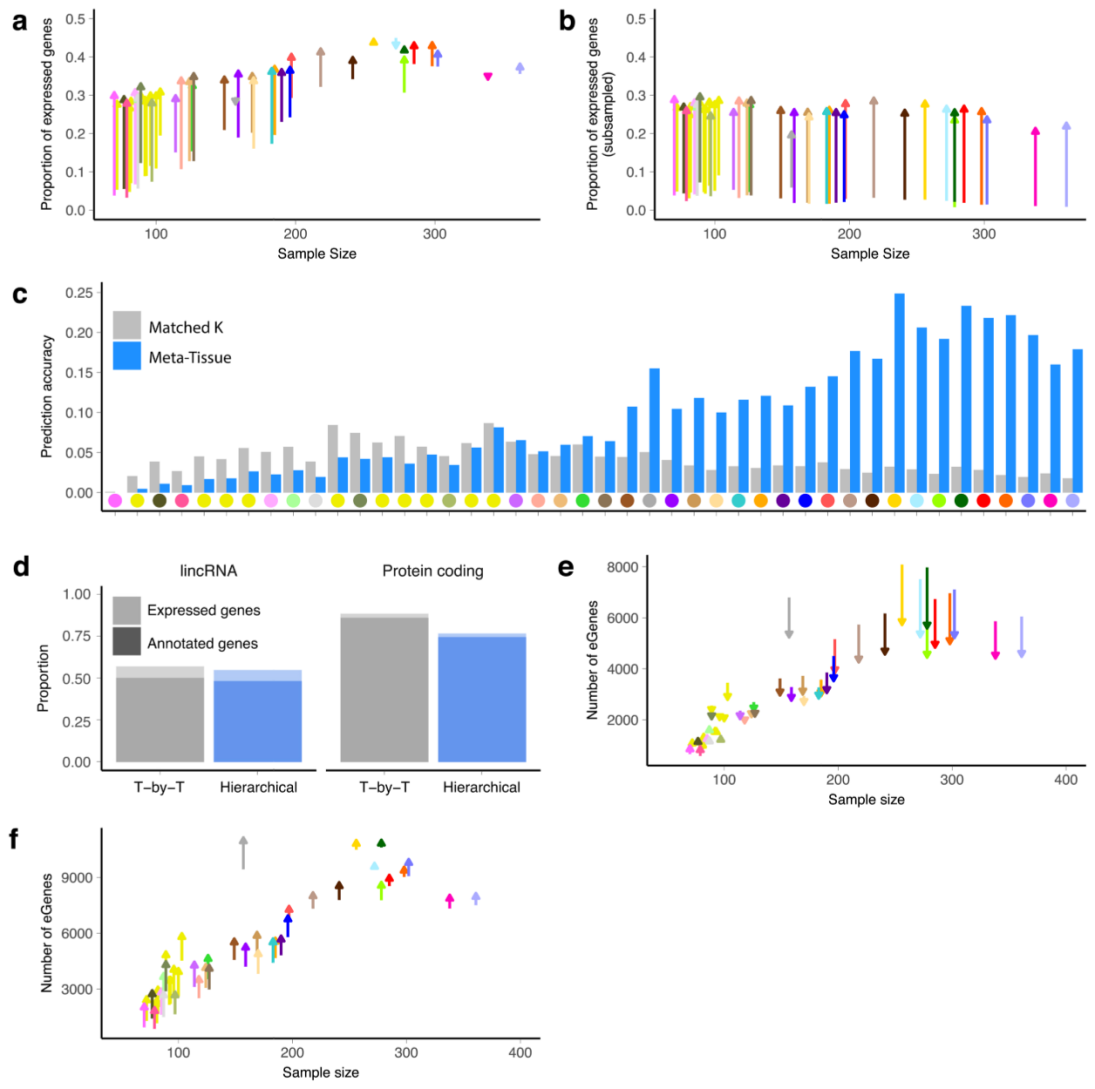
Sharing information across tissues for *cis*-eQTLs **a**, The proportion of expressed genes for which *cis*-eGenes are discovered in single tissues (5% FDR; origin) and the multi-tissue meta-analysis (*m* > 0.9; arrow), stratified by the sample size of individual tissues. In the meta-analysis, *cis*-eQTL discoveries are made using Meta-Tissue to identify tissues where the posterior probability a given *cis*-eQTL effect exists (that is, the tissue’s *m* value for the variant) is >0.9. **b**, The proportion of expressed genes that had a *cis*-eQTL in the subsampled data (*n* = 70) is shown on the *y*-axis, and the actual sample size of the tissue is shown on the *x*-axis. The proportion is shown for the tissue-by-tissue approach (5% FDR; origin) and using Meta-Tissue (*m* >0.9; arrow). **c**, For each of the subsampled tissue data sets (*n* = 70), we identified the additional *K* discoveries that were made using Meta-Tissue but were not significant at the 5% FDR threshold in the tissue-by-tissue analysis; we then identified the *K* most significant *cis*-eQTLs in the tissue-by-tissue analysis with a *q* value greater than 5% representing the additional discoveries we would make by simply relaxing the FDR. We then compared these two sets of *K cis*-eQTLs to the list of significant *cis*-eQTLs found in the full tissue-by-tissue analysis by calculating the proportion of the *K cis*-eQTLs that were significant in the full analysis (*y*-axis). The tissues are ordered along the *x*-axis by increasing sample size in the actual data set. **d**, The proportion of annotated and expressed genes that were found to be eGenes using the tissue-by-tissue approach and the hierarchical selection procedure implemented by TreeQTL. **e**, The number of *cis*-eGene discoveries per tissue (*y*-axis) against sample size (*x*-axis). The number of discoveries for the tissue-by-tissue approach are represented by the origin of each segment, while the number of discoveries from the hierarchical selection procedure are shown as arrows. As with Meta-Tissue, the hierarchical procedure improves *cis*-eGene discovery for tissues with low sample sizes, albeit fewer tissues overall have the benefits of this effect. Furthermore, an outcome of this procedure is that for tissues with high sample sizes, reported numbers of *cis*-eGenes are more conservative than those observed in the tissue-by-tissue analyses or Meta-Tissue. **f**, Improvement of *cis*-eGene discovery by incorporating genomic annotations. For the 26 tissues for which we can relate cell-type specific chromHMM annotations, we identify *cis*-eGenes accounting for the variant-level genomic annotations and corresponding enrichment estimates using the Bayesian FDR control procedure described previously^80^. For each tissue, the number of *cis*-eGenes identified by the Bayesian procedure (arrow) is plotted against the tissue-by-tissue results (origin).

Multi-tissue *cis*-eQTL analyses have been shown to increase power by explicitly modelling sharing patterns across tissues^15,18,20^. We did not observe an improvement in power for *trans*-eQTL discovery, consistent with the limited sharing observed across tissues (Extended Data Table 3). However, we did observe improvements for *cis*-eQTL discovery, particularly among tissues with smaller sample sizes (Extended Data Fig. 10). To ensure that these findings did not depend on the modelling assumptions of Meta-Tissue, we analysed the *P* values for all genes and all tissues with treeQTL, which controls the FDR of eGene discoveries across tissues^17^. This procedure identified 17,411 *cis*-eGenes at 5% FDR, 2,314 fewer eGenes than with the single-tissue analysis. Although this analysis is more conservative overall than the tissue-by-tissue analysis, we observed an increase in the number of eGenes detected in the tissues with the smallest sample sizes, including several brain regions, as well as an increase in the average number of tissues in which an eGene was detected (from 7.8 for single-tissue analysis to 8.3; Extended Data Fig. 10). Additional *cis*-eGenes identified through meta-analysis were more likely to be significant as sample size increased compared to similar numbers of eGenes identified using a less stringent single- tissue FDR (Extended Data Fig. 10). This suggests that one strategy for increasing power in studies of inaccessible or sample-limited cell types would be to analyse them jointly with data from GTEx tissues.

## Functional characterization of *cis*-eQTLs

**Figure 3:**
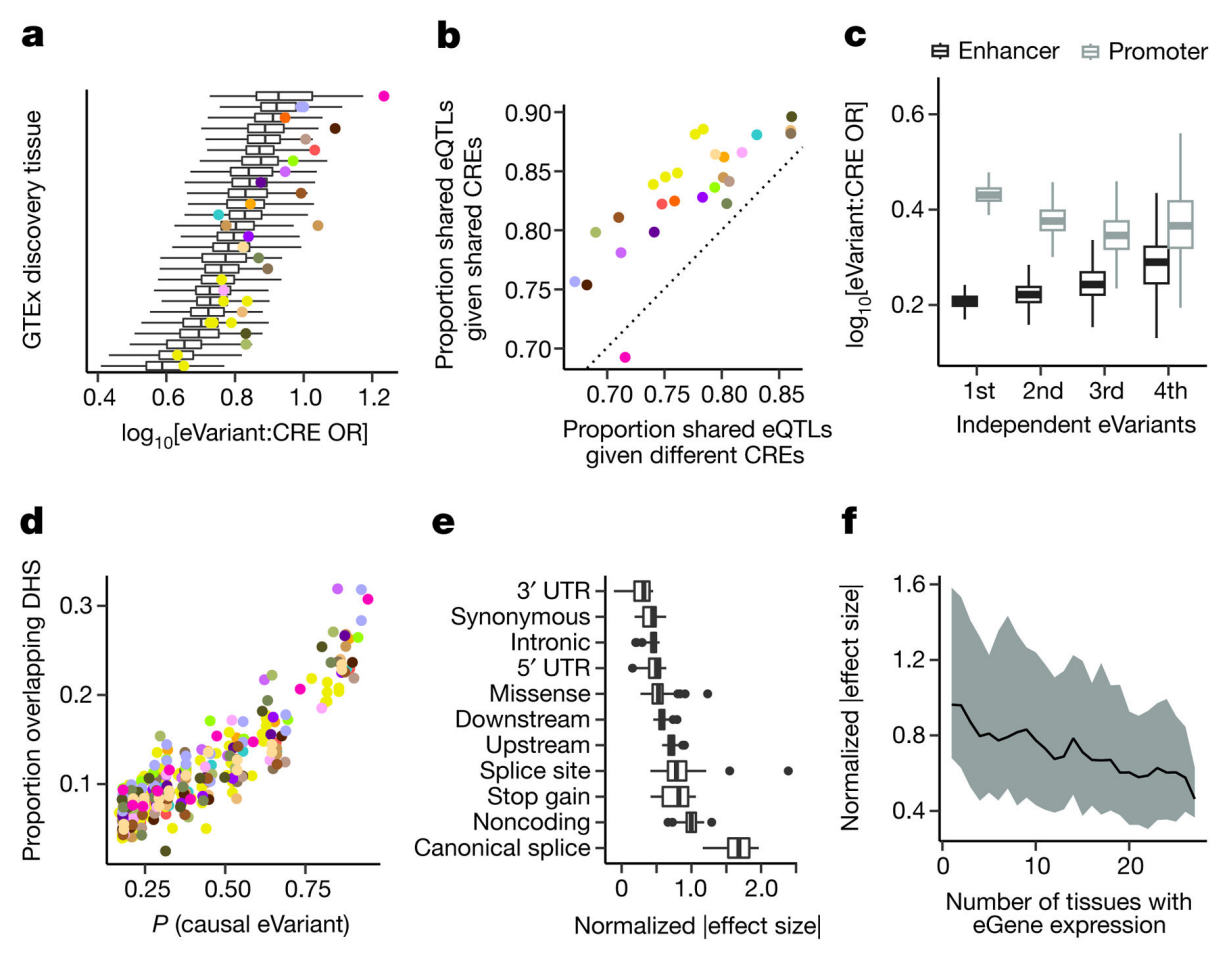
Functional characterization of *cis*-eQTLs **a**, Enrichment (*x*-axis) of eVariants in cis-regulatory elements (CREs) across 128 Roadmap Epigenomics project cell types, for each GTEx discovery tissue (*y*-axis). Enrichment estimated by comparing to random MAF- and distance-matched variants. Stronger enrichment was observed in matched tissues (coloured dots) than in unmatched tissues (box plots). **b**, Proportion of eQTLs shared between two tissues (*m* > 0.9) if the eVariant overlaps the same Roadmap annotation in both tissues (*y*-axis) or different annotations (*x*-axis). Points represent the mean across all tissues, coloured by the discovery tissue. **c**, Enrichment of eVariants (*y*-axis) in tissue-matched enhancers (black) and promoters (grey) for the first four conditionally independent eQTLs discovered for each eGene (*x*-axis). **d**, Proportion of eVariants overlapping tissue-matched DNase I hypersensitive sites (DHS; *y*-axis) as a function of the probability that a variant is causal (*x*-axis), coloured by the eQTL discovery tissue. **e**, Normalized absolute eQTL effect size (*x*-axis) for each eVariant annotation class (*y*-axis). **f**, Median (line) and interquartile range (shading) of normalized absolute eQTL effect size (*y*-axis), as a function of the number of tissues in which the eGene is expressed (*x*-axis). Box plots depict the interquartile range (IQR), whiskers depict 1.5× IQR. OR, odds ratio.

To characterize the biological properties of multi-tissue *cis*-eQTLs, we annotated discovered eVariants using chromatin state predictions from 128 cell types sampled by the Roadmap Epigenomics project^2^. eVariants were enriched in predicted promoter and enhancer states across all Roadmap cell types (Fig. 3a). However, the eVariants exhibited significantly greater enrichment in promoters and enhancers from matched tissues (Wilcoxon rank sum test, *P* ≤ 9.3 ×10^−4^, Extended Data Table 4), illustrating consistent patterns of tissue specificity for *cis*-regulatory elements and eQTLs (Fig. 3a). Furthermore, eQTL activity was significantly more likely to be shared across pairs of tissues when the eVariant overlaps the same chromatin state in both tissues (Wilcoxon rank sum test, *P* ≤ 5.0×10^−5^, Fig. 3b).

Integration of genomic annotations such as chromatin state has been demonstrated to improve power for eQTL discovery^8,21–23^. For 26 GTEx tissues matched with cell-type specific annotations from the Roadmap Epigenomics project, we applied a Bayesian hierarchical model for eQTL discovery by incorporating variant-level genomic annotations^24^ that provided a substantial boost to discovery power. Inclusion of genomic annotations (enhancers, promoters and distance to the TSS) increased the total number of *cis*-eQTL discoveries by an average of 43% (or 1,200 genes) per tissue (Extended Data Fig. 10f), demonstrating the considerable advantage of integrating genomic annotations into eQTL mapping models.

Conditionally independent (secondary) *cis*-eQTL signals were located further from the TSS (median distance 50.1 kb from the TSS, compared to 28.9 kb for primary eQTLs; Wilcoxon rank sum test, *P* ≤ 2.2×10^−16^). However, similar to primary eVariant associations, secondary eVariants were enriched for chromosomal contact with target eGene promoters, as determined through Hi-C, compared to background variant–TSS pairs (Supplementary Information 6). This suggests that, despite their sequence-based distance from the TSS, primary and secondary eVariants are in close physical contact with their target gene promoters via chromatin looping interactions. While primary eVariants were significantly more enriched in promoters than enhancers, secondary associations exhibit increased enhancer enrichment, consistent with their increased distance from the TSS and tissue-restricted activity (Wilcoxon rank sum test, *P* ≤ 2.2×10^−16^, Fig. 3c).

To identify causal variants that are likely to underlie *cis*-eQTLs, we applied two computational fine-mapping strategies^25,26^ (Supplementary Information 13 and Supplementary Figs 11, 18). First, we identified 90% credible sets (that is, the collection of variants with 90% probability of containing all causal variants) for each eGene in each tissue using CAVIAR^25^. Across all tissues, the mean credible set size was 29 variants (per tissue means ranged from 25 to 31). Second, we estimated the probability that each eVariant is a causal variant using CaVEMaN^26^. Across tissues, between 3.5% and 11.7% of top eVariants were predicted to be causal variants (causal probability *P* > 0.8). Consistent with variants with high causal probabilities being functional regulatory variants (as opposed to linkage disequilibrium proxies), 24.3% of eVariants with causal probabilities in the top tenth percentile (0.77 <*P* < 1) lay in open chromatin regions, while only 6.56% of eVariants in the lowest tenth percentile (0.0266 <*P* < 0.189) lay in such regions (Fig. 3d).

**Extended Data Figure 11:**
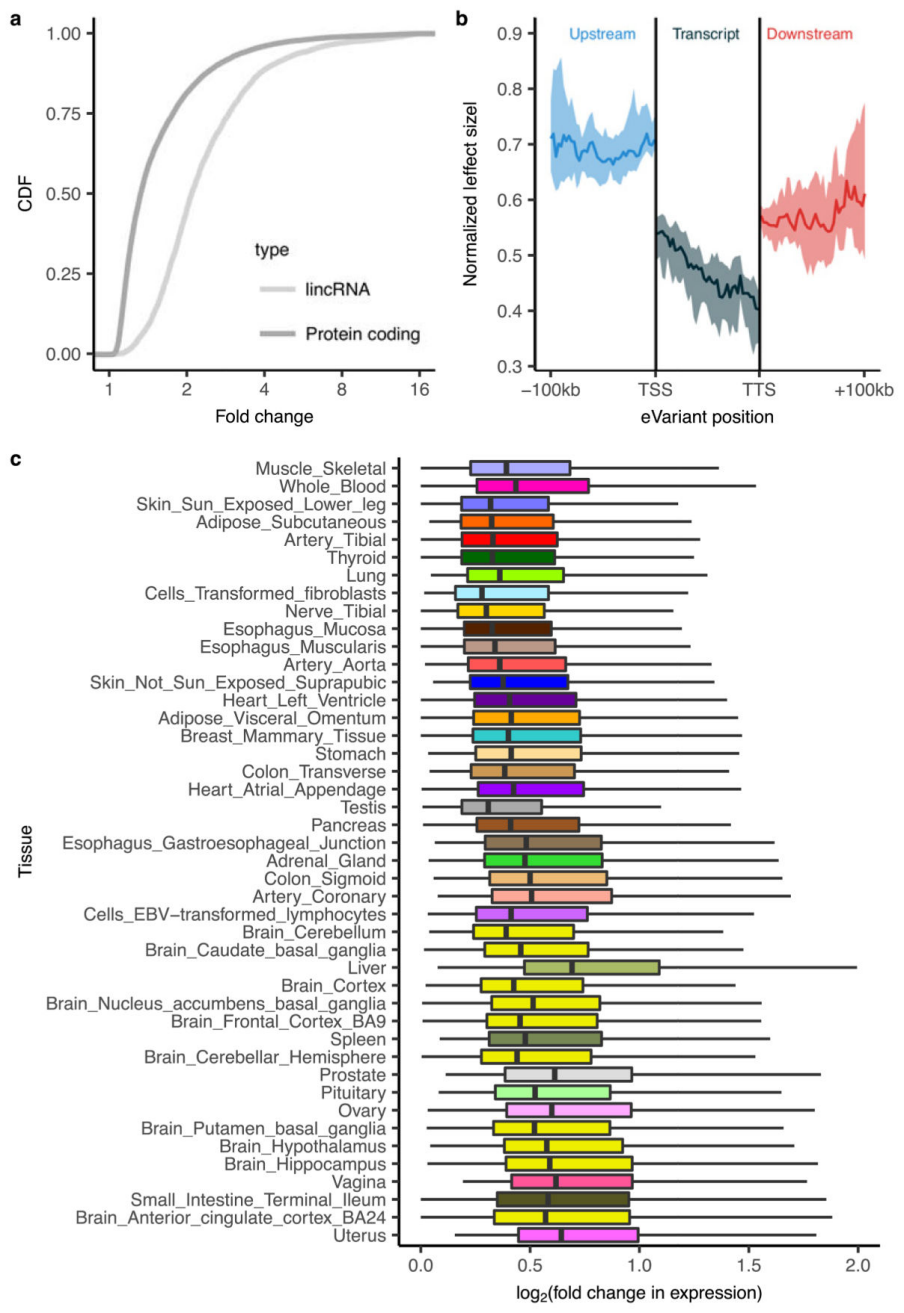
*cis* effect size analyses **a**, For each autosomal protein-coding and lincRNA *cis*-eGene with eVariants discovered independently in at least five tissues, the median effect size was computed across these tissues. The empirical cumulative distribution function (CDF) of these median effect sizes is depicted. **b**, Normalized effect sizes of *cis*-eVariants located upstream of the gene, within the transcript, and downstream of the gene. **c**, *cis*-eQTL effect distributions stratified by discovery tissue. Tissues are sorted from largest sample size (muscle-skeletal, *n* = 361) to the smallest (uterus, *n* = 70). Box plots depict the IQR, whiskers depict 1.5× IQR.

To determine the effect sizes of *cis*-eQTLs, we used allelic fold change, a method that assumes an additive model of eQTL alleles on total gene expression, allowing for interpretation of effect sizes as a fold change between alleles^27^ (Supplementary Information 14). 17.4% of eGenes had *cis*-eQTLs with median effect sizes of at least twofold across tissues (Extended Data Fig. 11a, c). The prevalence of many ≥ twofold effects highlights the large impacts that common regulatory variants can have on gene dosage. *cis*-eVariants at canonical splice sites exhibited the strongest effects, followed by variants in noncoding transcripts, while variants in the 3′ UTR had the weakest effects (Fig. 3e).

Analysis of eQTL effect sizes around the TSS demonstrated that, as a group, upstream variants had the strongest effects, while those within transcripts had the weakest effects (Extended Data Fig. 11b). This suggests that eVariants that are likely to affect transcription have stronger effects on gene expression levels than variants that are likely to affect post-transcriptional regulation of mRNA levels. A notable exception is splice site and stop-gained variants, which make up a small number of total eQTLs but have large effects on expression levels (presumably owing to nonsense-mediated decay). When genes are stratified by the number of tissues in which they are expressed, the average effect size decreases as the number of tissues increases, indicating that genes expressed in greater numbers of tissues are less likely to have eQTLs with large regulatory effects (Spearman’s *ρ* =−0.29, *P* ≤ 2.2×10^−16^; Fig. 3f).

ASE provides an independent measure of a *cis*-eVariant’s effect size. We estimated the effects of the primary eVariant for each eGene by applying allelic fold change to ASE measurements (see Methods). Effect size estimates from both total and allele-specific expression approaches were highly correlated (mean Spearman’s *ρ* =0.82, s.d.= 2%) with an average ratio of eQTL effect sizes to ASE effect sizes of 0.937 (s.d. =6%; Extended Data Fig. 6b, c). This observation suggests that *cis*-eQTLs and ASE capture the same regulatory effects.

## Functional characterization of *trans*-eQTLs

**Extended Data Figure 12:**
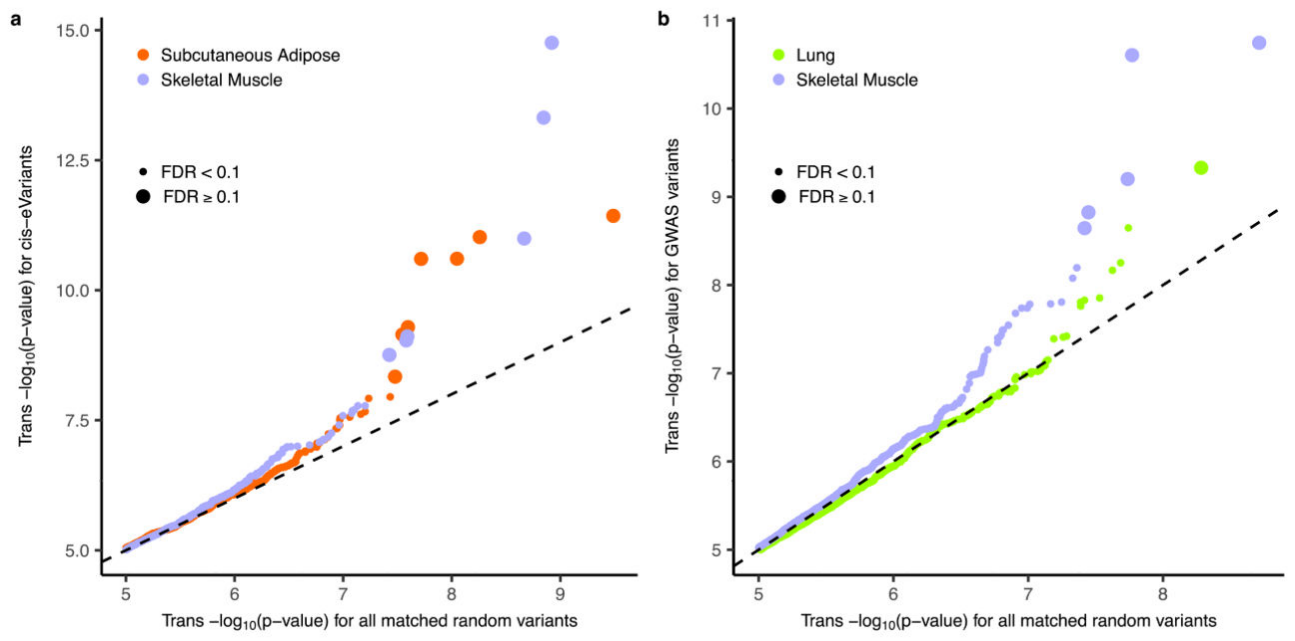
*trans*-eQTL discovery restricted to informed subsets **a**, Quantile–quantile (QQ) plot of *trans*-eQTL *P* values from all variants (*x*-axis) and the subset of *trans*-eQTL *P* values restricted to *cis*-eVariants (*y*-axis), illustrating enrichment of low *trans*-eQTL association *P* values for *cis*-eVariants. Data are plotted separately for skeletal muscle (pale blue) and adipose (red). *trans*-eQTLs with FDR ≤ 10% are shown as large circles, those with FDR > 10% are shown as small circles. **b**, QQ plot of *trans*-eQTL *P* values from all variants (*x*-axis) and the subset of *trans*-eQTL *P* values restricted to GWAS associated variants (*y*-axis), illustrating enrichment of low *trans*-eQTL association *P* values for *cis*-eVariants. Data are plotted separately for skeletal muscle (pale blue) and lung (green). *trans*-eQTLs with FDR ≤ 10% are shown as large circles, those with FDR > 10% are shown as small circles.

To better understand the cellular mechanisms of *trans*-eQTLs, we characterized several of their functional properties. Of the 673 *trans*-eQTLs from the genome-wide analysis, 161 also had a *cis*-association (at a *cis P* value threshold of *P* ≤ 1.0×10^−5^) with 113 unique variants, yielding the set of 296 unique trios of an eVariant, a *cis*-eGene and a *trans*-eGene. This suggests a common mechanism for *trans* regulation in which the eVariant directly regulates expression of a nearby gene whose protein product then affects other genes downstream. Considering this observation, we ran a restricted test for *trans*-associations, limiting variants to the set of significant *cis*-eVariants (Extended Data Fig. 12a). From this, we identified a total of 33 *trans*-eGenes (10% FDR) among this subset of tests, 14 of which were not discovered in the genome-wide analysis (Supplementary Information 10). There were substantially more *trans*-eQTLs at 50% FDR from this *cis*-eVariant restricted test than random variants matched for MAF and distance to TSS and stratified by tissue (Cochran–Mantel–Haenszel test, *P* ≤ 2.2×10^−16^).

**Figure 4:**
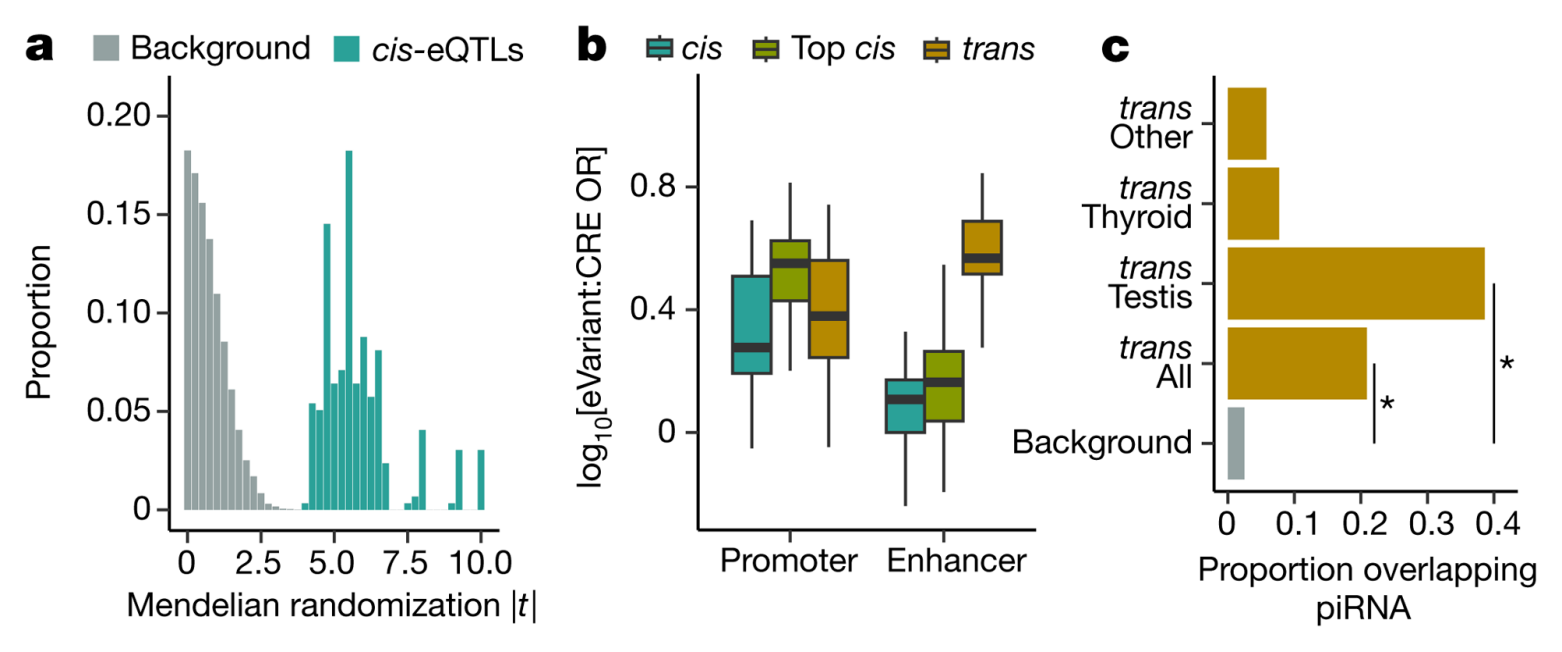
Functional characterization of GTEx *trans*-eVariants **a**, Frequency distribution of Mendelian randomization *t*-statistic, for 296 *cis*–*trans*-eQTLs and matched background variants. **b**, CRE enrichment (*y*-axis) of *trans*-eVariants (10% FDR), *cis*-eVariants (10% FDR, to match *trans*-eVariants), and top most significant *cis*-eVariants. Box plots show promoter and enhancer enrichment (*x*-axis) in matched cell-type CRE annotations compared to MAF- and distance-matched background variants. **c**, Proportion (*x*-axis) of variants overlapping piRNA clusters, including randomly sampled background loci, *trans*-eVariants across all tissues, testis *trans*-eVariants, thyroid *trans*-eVariants, and *trans*-eVariants from all tissues other than testis and thyroid. Asterisks denote significant enrichment (permutation test, *P* ≤1.0 ×10^−4^). Box plots depict the IQR, whiskers depict 1.5× IQR.

We performed Mendelian randomization on the full set of 296 *trans*-eQTLs matched with a unique corresponding *cis*-eGene, measuring the causal impact of the *cis*-eGene on the *trans*-eGene, using the eVariant as the randomized instrumental variable (Supplementary Information 15). For *trans*-eQTLs with a *cis*-eGene, we observed strong evidence for regulation of the *trans*-eGene expression via the *cis*-eGene (Fig. 4a; *P* values ranging from *P* ≤ 3.0 × 10^−5^ to *P* ≤ 2.2 × 10^−16^). *trans*- eVariants with no *cis*-eGene may alter protein function, may reflect false negatives in the *cis* association test, or may arise from unmeasured regulatory mechanisms. Protein-coding loci were not enriched among our *trans*-eVariants (odds ratio 0.94; Fisher’s exact test, *P* ≤ 0.80), suggesting that modification of protein function is not the dominant mechanism for *trans*-eQTL effects.

We investigated whether *trans*-eVariants were each associated with numerous target genes, which may reflect broad effects of regulatory loci, as have been reported in model organisms^5,28^. Disambiguating true broad regulatory effects from artefacts remains an important challenge^29^. In our primary analysis, we applied aggressive correction of potential confounders, controlling for 15–35 probabilistic estimation of expression residuals (PEER) factors^12^ capturing 59–78% of total variance in gene expression levels (Supplementary Information 5). However, PEER and related approaches^30^ may also remove variance in gene expression levels arising from regulatory pathways and broad *trans* effects. Indeed, several loci with numerous associations were found in uncorrected data, but disappeared after controlling for PEER factors (Supplementary Fig. 13). Associations found in uncorrected data are likely to include many false positives for three reasons: 1) the PEER factors were strongly associated with known technical confounders (Extended Data Fig. 1 and Supplementary Figs 8, 9); 2) *trans*-eVariants identified from raw data and lost after correction were enriched for association with technical covariates (Supplementary Fig. 14); and 3) no other parameter setting clearly optimized *trans*-eQTL discovery (Supplementary Fig. 12). Even after PEER correction, we observed evidence of eVariants with multiple targets; at genome-wide significance, four separate loci were associated with more than one *trans*-eGene each (Supplementary Table 14).

We quantified the enrichment of *trans*-eVariants in promoter and enhancer regions using the same tissue-specific annotations from the Roadmap Epigenomics project^1,2^ used for *cis*-eQTL analysis (Extended Data Table 4). *trans*-eVariants (10% FDR) were enriched in cell-type matched enhancers (median Fisher’s exact test, *P* ≤ 2.2 ×10^−3^) but not strongly enriched for promoters (median *P* ≤ 0.22), compared to randomly selected variants matched by distance to nearest TSS, MAF and chromosome (Fig. 4b). *trans*-eVariants were more enriched than *cis*-eVariants at matched FDR (Wilcoxon rank sum test, promoter: *P* ≤ 4.6 ×10^−7^; enhancer: *P* ≤ 2.2 ×10^−16^). Stronger effect sizes are needed to detect *trans*-eVariants at the same FDR, but even comparing to a matched number of the strongest *cis*-eVariants, we observed greater enrichment in enhancer (but not promoter) regions among *trans*-eVariants, consistent with greater tissue-specificity of enhancer activity and *trans*-eVariants^31^ (Fig. 2c).

Given the large number of *trans*-eQTLs detected in testis, we investigated their possible regulatory mechanisms in more detail. Piwi-interacting RNAs (piRNAs) are small 24–31-bp RNAs that bind to piwi-class proteins and silence mobile elements by RNA degradation and DNA methylation. PiRNAs are strongly expressed in testis and may regulate gene expression and play a role in protection against transposable elements in germ line cells^32^. We tested for enrichment of *trans*-eVariants in piRNA clusters identified in testis^33^. We found that 38.6% of testis *trans*-eVariants, corresponding to 12 independent loci (*R*^2^ ≤ 0.2), directly overlapped piRNA clusters, a significant enrichment compared to the 2.5% of the genome covered by these regions (permutation test, *P* ≤ 1.0 × 10^−4^). In aggregate, eVariants from all tissues were enriched in piRNA clusters (permutation test, *P* ≤1.0×10^−4^), but this appeared to be almost entirely driven by testis eQTLs (Fig. 4c). This suggests a testis-specific functional effect of genetic variation in piRNA clusters, consistent with their biological role.

## Replication of eQTLs

**Extended Data Figure 13:**
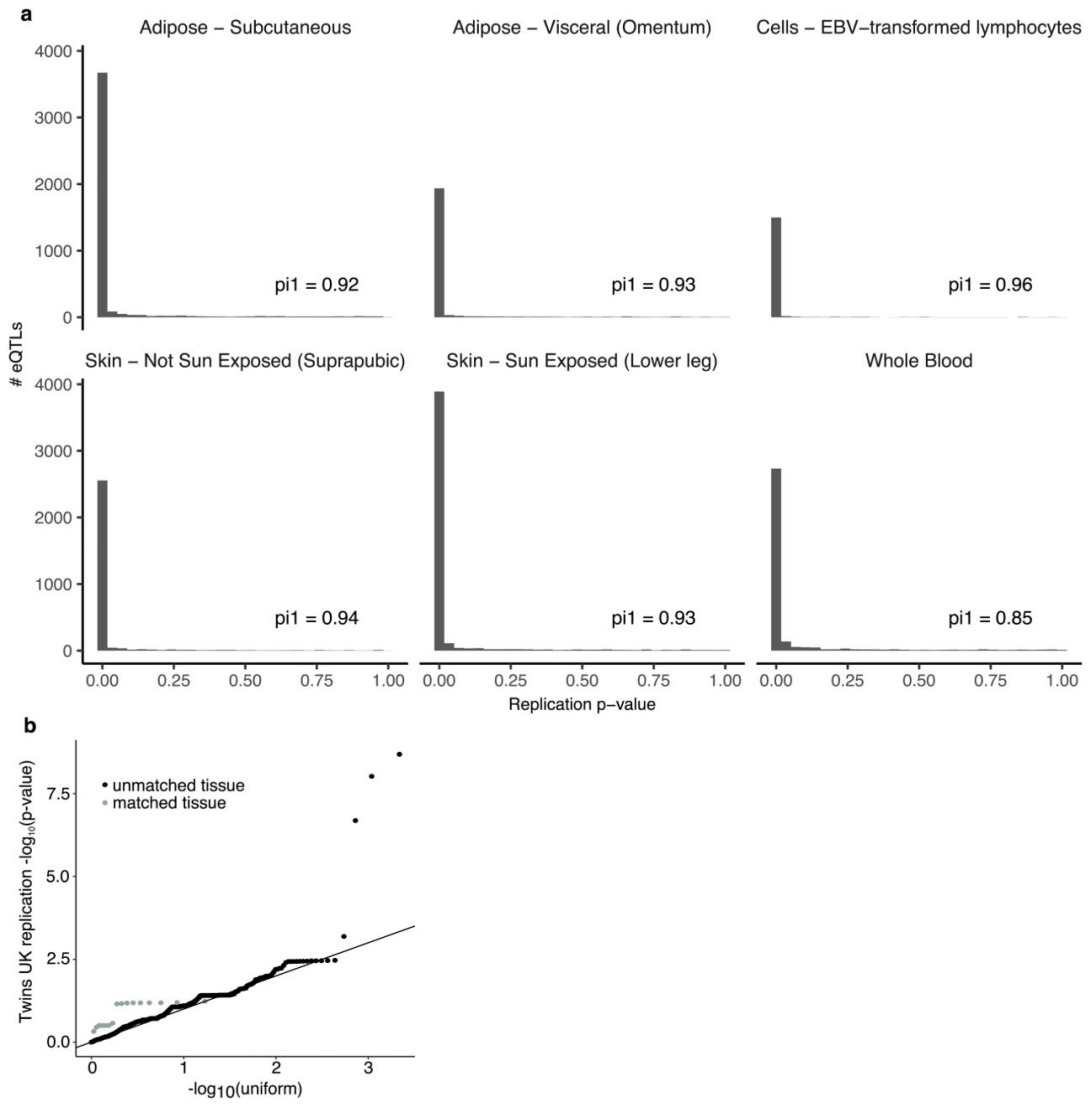
Replication of *cis*-eQTLs in TwinsUK data **a**, All *cis*-eQTLs (5% FDR) from six tissues (adipose, subcutaneous; adipose, visceral omentum; cells, EBV-transformed lymphocytes; skin, not sun-exposed; skin, sun-exposed; and whole blood) were examined for replication in four closely matched tissues (LCLs, skin, whole blood, subcutaneous adipose) from the TwinsUK data. For each tissue pair (in facets), replication *P* value histograms illustrate strong enrichment of small *P* values. *π*_1_ statistics are provided for each tissue pair. **b**, All *trans*-eVariant–eGene pairs (10% FDR) from all tissues were examined for replication in four closely matched tissues (LCLs, skin, whole blood, subcutaneous adipose) from the TwinsUK data. Observed replication *P* values (*y*-axis) are plotted against the expected uniform *P* value distribution under the null hypothesis (*x*-axis). Replication *P* values are plotted separately for matched (grey) and unmatched (black) tissue pairs.

To assess the replicability of the identified *cis*-eQTLs, we compared our results to four matched tissues from the Twins UK project^34^ (Supplementary Information 16). The vast majority of GTEx *cis*-eQTLs replicated at 5% FDR (Extended Data Fig. 13a; 84% in whole blood, 87% in subcutaneous adipose, 94% in lymphoblastoid cell lines (LCLs), and 93% in sun-protected skin). *trans*-eQTLs have not replicated consistently in human studies, compared to *cis*-eQTLs^21,35–37^, owing in part to insufficient statistical power and a limited number of studies with comparable tissues and cohorts, but also reflecting potential false positive associations. We tested *trans*-eQTLs discovered at 10% FDR in GTEx for replication in the TwinsUK data. Five hundred and sixty GTEx *trans*-eQTLs were testable in the four TwinsUK tissues and, of these, three *trans*-eQTLs replicated at 10% FDR (Supplementary Table 15). Despite the small number that individually replicated, in aggregate, the full set of *trans*-eQTLs demonstrated significantly greater replication than expected by chance (Wilcoxon rank sum test on association *P* values compared to uniform; *P* ≤3.05 ×10^−5^ for 16 tests in matched tissues; *P* ≤2.2 ×10^−16^ for 2,176 tests across all four TwinsUK tissues). In addition, aggregate replication of *trans*-eQTLs was significantly stronger for matched tissue types than for unmatched tissue types (Wilcoxon rank sum test; *P* ≤1.54×10^−4^; Extended Data Fig. 13b).

Finally, we replicated two tissue-specific *trans*-eQTLs highlighted in the TwinsUK Multiple Tissue Human Expression Resource (MuTHER) study^38,39^ (*n* = 845 donors in three tissues: subcutaneous adipose, LCLs and skin). First, in sun-exposed skin in GTEx, rs289750 was associated in *cis* with *NLRC5* (association *P* ≤4.7 ×10 ^−16^) and in *trans* with *TAP1* (association *P* ≤9.0 ×10 ^−10^, 4.3% FDR in the *cis*-eQTL restricted *trans*-eQTL discovery set), while the TwinsUK study found rs289749 (located 469 bp away from rs289750; *R*^2^ =0.918) associated in skin samples with *NLRC5* in *cis* (association *P* ≤2.2 ×10 ^−16^) (ref. 38) and *TAP1* in *trans* (tensor association *P* ≤4.3 ×10 ^−7^) (ref. 39). Second, the MuTHER study identified a master regulator in subcutaneous adipose, rs4731702, associated with the maternally expressed *cis* target gene *KLF14*, which encodes the transcription factor Kruppel-like factor^38,40^. *cis*-eQTL rs4731702 showed enriched association with genes that are relevant in metabolic phenotypes, such as cholesterol levels. In the GTEx data, rs4731702 is in strong linkage disequilibrium with two variants, rs13234269 and rs35722851 (*R*^2^ =0.98 and 0.99, respectively), that are *cis*-eQTLs for *KLF14* in subcutaneous adipose (*P* ≤2.2×10^−5^ and *P* ≤4.7×10^−5^, respectively). We evaluated the association of rs13234269 with all expressed genes in GTEx subcutaneous adipose (17,633 genes). Although we found no individually significant *trans*-eGenes, we found an enrichment of association with distal gene expression in subcutaneous adipose tissue (*π*_1_ =0.07, after PEER correction), replicating the results of the MuTHER study.

## Expression QTLs and complex disease associations

Overlaps between genome-wide association study (GWAS) associations and eQTLs have provided important insights into regulatory genes and variants for a wide range of complex traits and diseases^5^. As both the presence and extent of overlap between GWAS and eQTLs can be tissue- specific, the current phase of GTEx overcomes a major limitation in interpretation of disease variants by enabling analysis across a broad range of tissues.

**Extended Data Figure 14:**
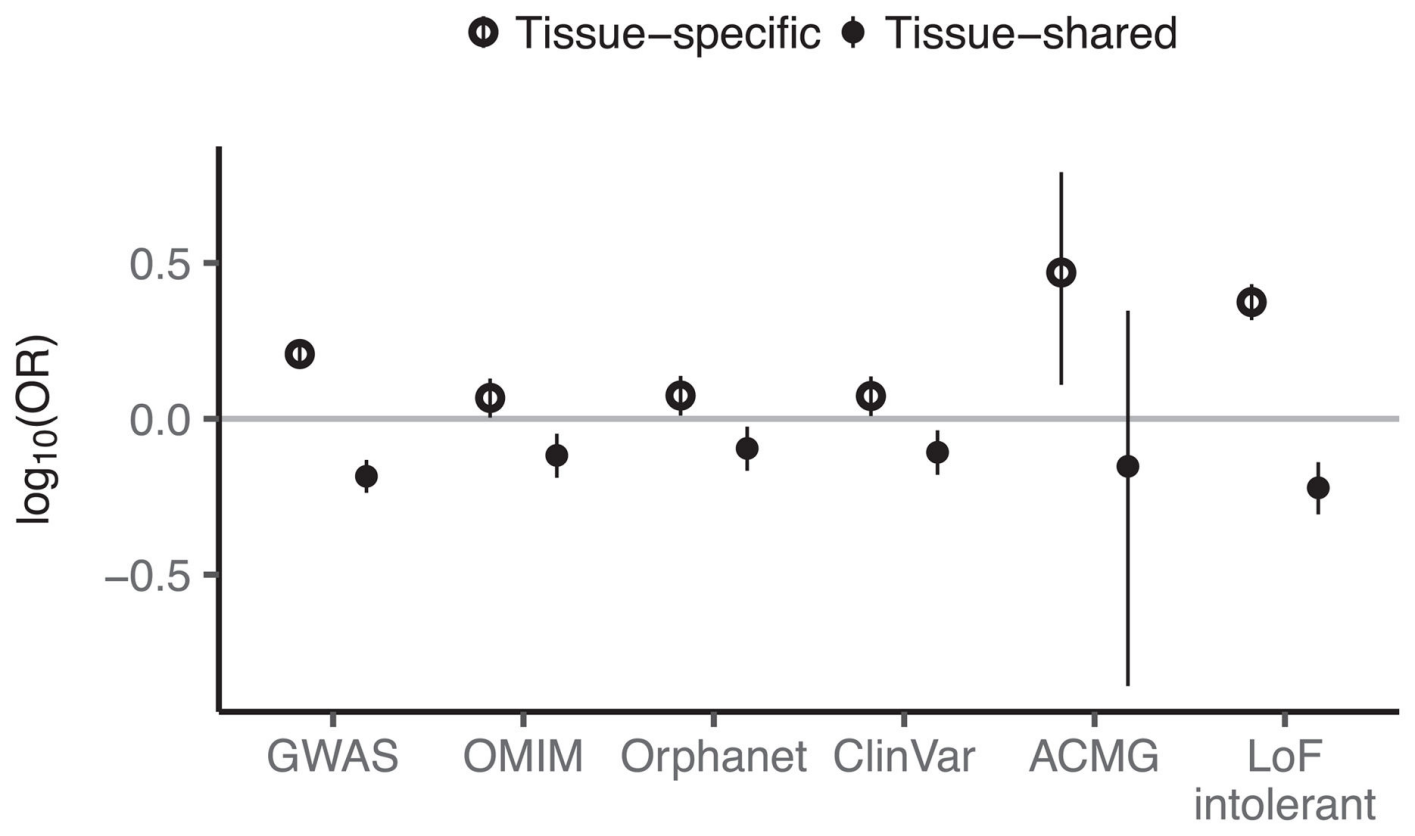
Disease gene enrichment for tissue-specific and shared *cis*-eQTLs Enrichment of shared and tissue-specific *cis*-eGenes in different disease gene data sets. Enrichments and 95% CI in each data set are calculated via Fisher’s exact test, and the odds ratio is plotted after log_10_ transformation.

**Figure 5:**
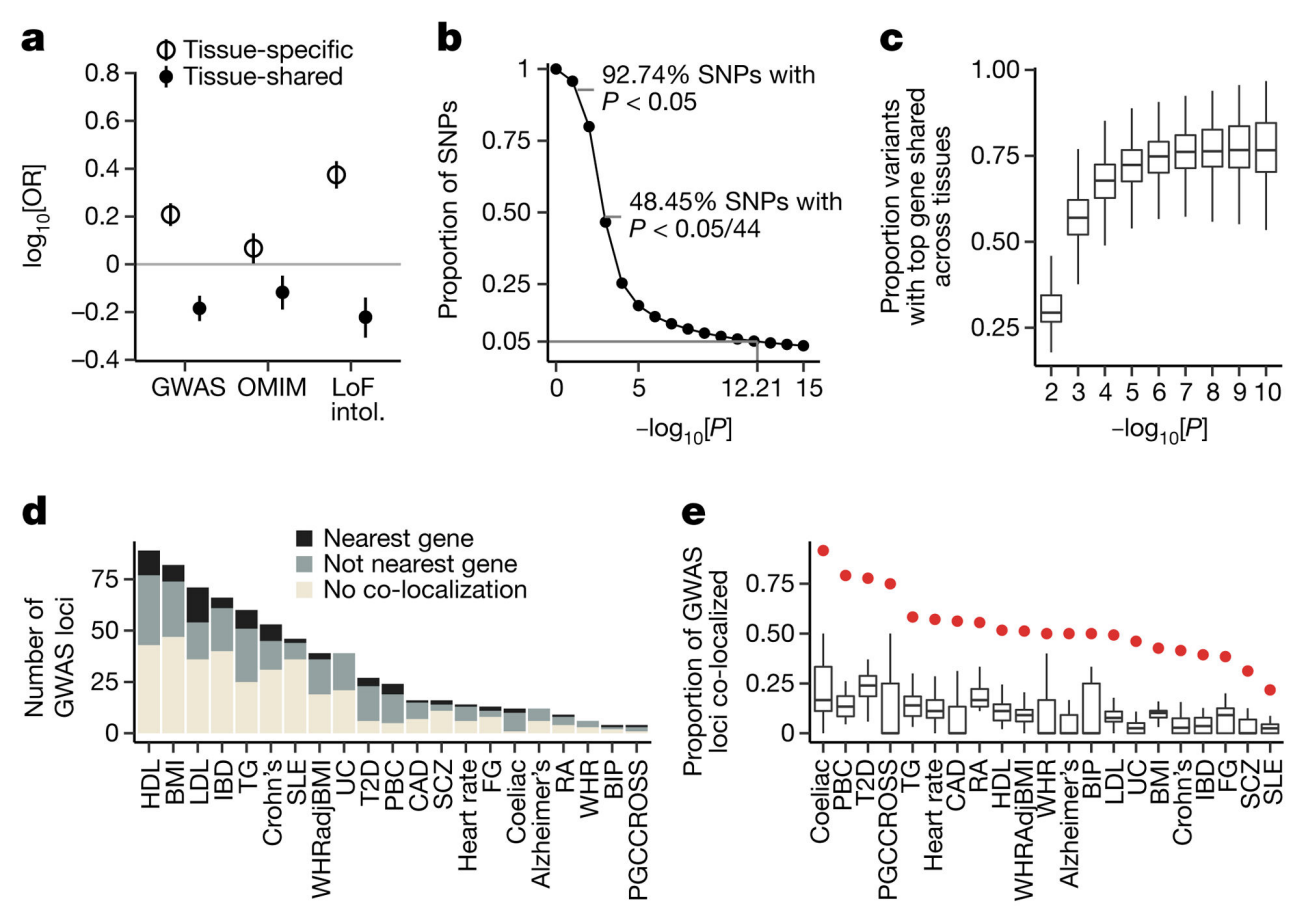
Properties of *cis*-eQTL overlap with complex trait associated loci **a**, Enrichment of tissue-specific and tissue-shared eGenes in disease and loss-of-function mutation intolerant genes. Tissue-specific and shared eGenes were defined as eGenes in the bottom and top 10% of the distribution of proportion of tissues with an eQTL effect. Bars represent 95% confidence intervals. **b**, Proportion of eQTLs (*y*-axis) discovered as a function of *P* cutoffs (*x*-axis). **c**, Proportion of variants (*y*-axis) with top associated protein-coding gene shared between tissues at varying *P* thresholds (*x*-axis). **d**, Number of GWAS loci (*y*-axis) and their co-localization results for each of 21 traits (*x*-axis), coloured by whether the eGene is the closest expressed gene to the lead GWAS variant. **e**, Proportion of GWAS loci (*y*-axis) with a significant co-localization for each of 21 traits (*x*-axis). Box plots depict the proportion explained in each of 44 tissues, red dots depict the proportion explained by the union of all tissues. Box plots depict the IQR, whiskers depict 1.5× IQR.

We observed that the degree of tissue sharing of an eQTL is associated with several indicators of phenotypic impact. Tissue-shared eGenes are depleted from loss-of-function mutation-intolerant genes (as curated by ExAC^41^) (Fig. 5a), consistent with purifying selection removing large-effect regulatory variants that involve many tissues. Tissue-shared eGenes were also less likely than tissue-specific eGenes to be annotated disease genes (Fisher’s exact test, nominal *P* ≤10^−6^ for GWAS, Online Mendelian Inheritance in Man (OMIM), and loss-of-function-intolerant gene sets; Fig. 5a, Extended Data Fig. 14), highlighting the importance of broad tissue sampling for GWAS interpretation.

**Extended Data Figure 15:**
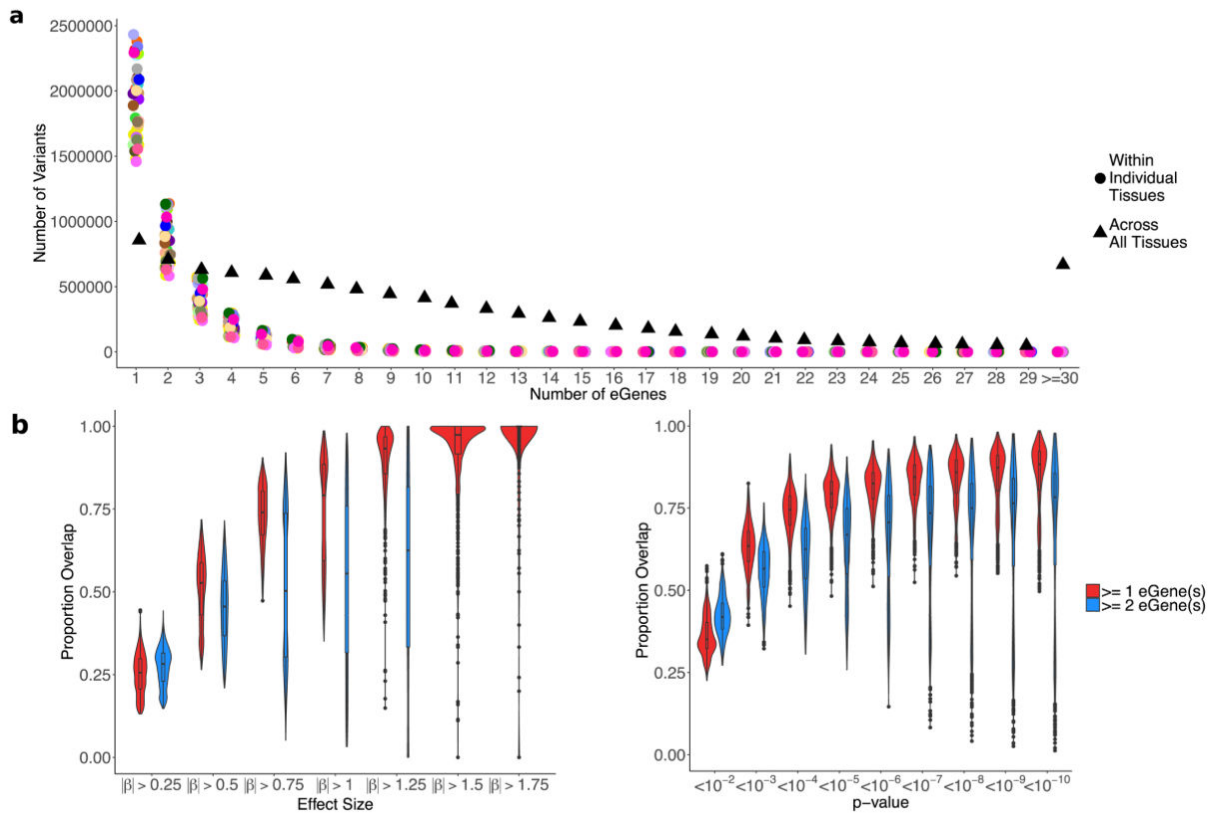
General and replicated per-variant *cis*-eGene associations across tissues **a**, Distribution of the number of unique *cis*-eGenes per variant within each tissue (points) or in the union of variant–eGene associations across all tissues. **b**, Proportion of variants with top-associated gene preserved between tissues for varying effect size thresholds, across all pairwise tissue comparisons. **c**, Proportion of variants with top-associated gene preserved between tissues for varying nominal *P* value thresholds, across all pairwise tissue comparisons. Red distributions include all variants with an associated *cis*-eGene in one of the two compared tissues, and blue distributions require the variant to have at least two associated *cis*-eGenes in each tissue. Distributions for which more than half of the pairwise comparisons (points) are empty are not shown.

This broad tissue sampling affects the use of eQTL data for the interpretation of GWAS variants. We observed that a GWAS variant of interest is likely to be a *cis*-eQTL by chance. Of all common variants assayed within GTEx, 92.7% are nominally associated with the expression of one or more genes in one or more tissues (*P* < 0.05) and nearly 50% are significant when correcting for the number of tissues tested (Fig. 5b). Furthermore, linking an eQTL signal to a specific gene becomes increasingly complicated with abundant eQTL data. Some variants are associated with more than 30 different nearby genes (Extended Data Fig. 15a). Furthermore, even restricting to strong associations (*P* <10^−10^ in each tissue), for over 10% of eVariants, the gene with the strongest association varies between tissues (Fig. 5c; Extended Data Fig. 15b, c). These results reinforce the need for caution when using eQTL data to interpret the function of GWAS variants.

To assess the extent to which GTEx *cis*-eQTLs are responsible for common phenotypic variation, we applied co-localization analysis to examine local linkage disequilibrium and sharing of association signals using GWAS summary statistics across 21 traits^42–44^ (Supplementary Table 16). Among tested loci, 52% of trait-associated variants co- localized with an eQTL in one or more tissues (Fig. 5d, e). Importantly, no single tissue explained the majority of trait-associated loci, but the breadth of GTEx tissue sampling identified more co-localizations than any single tissue alone. Seven per cent and 93% of co-localizations are with lincRNA and protein-coding eGenes, respectively, suggesting that lincRNAs have a limited role in common disease pathogenesis. However, several findings complicate the interpretation of GWAS–eQTL overlaps. First, 26% of GWAS loci co-localize with more than one distinct eGene (that is, half of all co-localized loci). Second, GWAS co-localized eGenes are shared across an average of four tissues. Third, similar to lead eVariants, only 40% of GWAS signals co-localize with their nearest expressed gene, a finding that has important implications for the functional characterization of GWAS results.

Genetic variants associated with complex traits have been suggested to be enriched for *trans*-eQTLs^6,44–47^. Accordingly, we performed *trans*-eQTL mapping, restricting it to variants associated with a complex trait in a GWAS (Extended Data Fig. 12b). In this analysis, across the 44 tissues, we found 29 *trans*-eQTL associations involving 24 unique variants and 25 unique genes (10% FDR; Fig. 4a), each specific to a single tissue. There were more *trans*-eVariants at 50% FDR with association in at least one tissue when testing was restricted to trait-associated variants compared with random variants matched by MAF and distance to TSS (Fisher’s exact test, *P* ≤1.3×10^−3^).

**Extended Data Figure 16:**
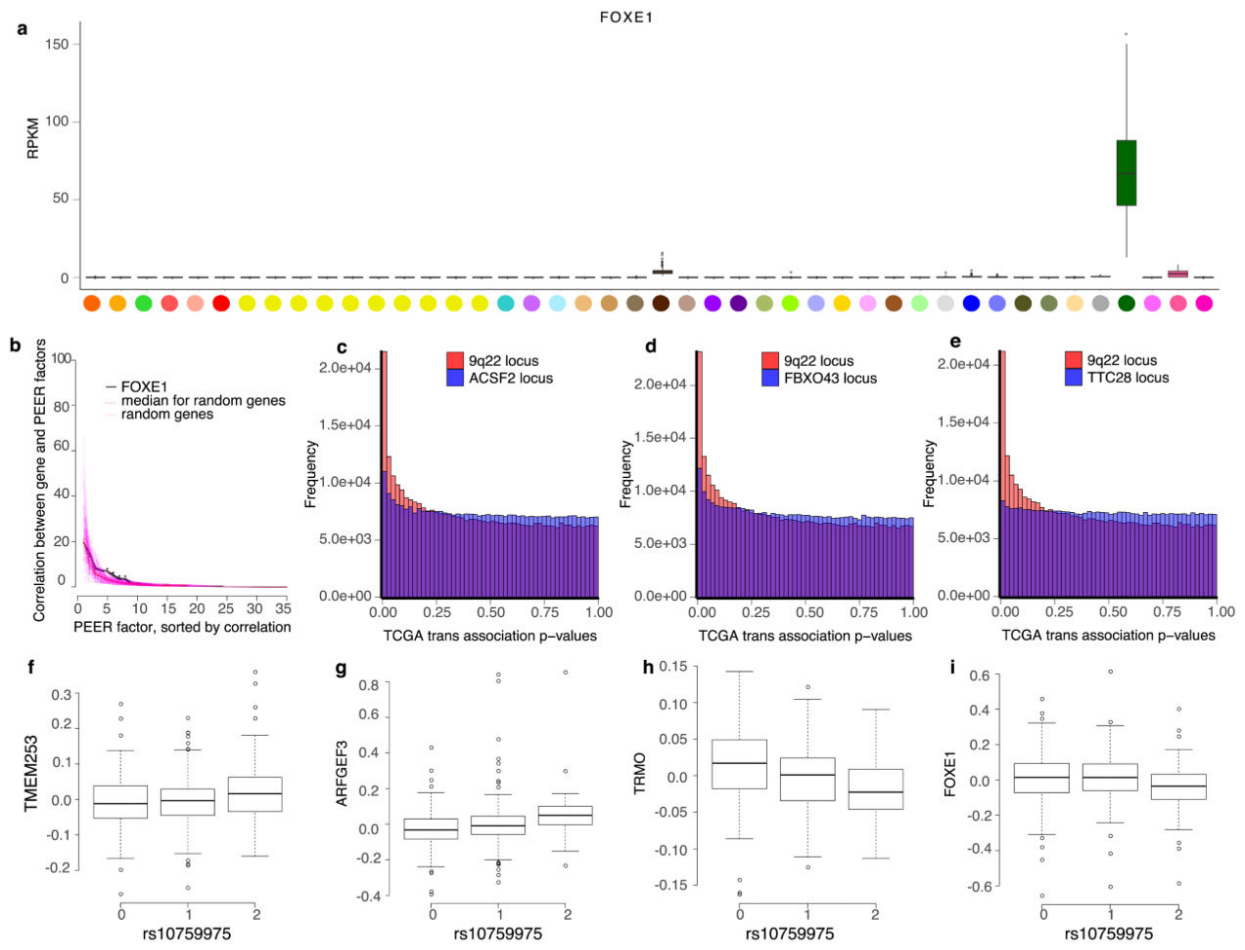
Broad *trans*-regulatory locus 9q22 in thyroid tissue **a**, *FOXE1* expression is thyroid-specific. **b**, Correlation between *FOXE1* expression levels and thyroid PEER factors, compared to 100 random genes. For every gene, absolute correlation was sorted in decreasing order. The correlation of *FOXE1* with the fifth, sixth, seventh and eighth PEER factors was significantly higher than the correlation of random genes at those rank ordered PEER factors (empirical *P* ≤ 0.05). **c**–**e**, Variants in the chr 9q22 locus were enriched for association with genes on other chromosomes in thyroid carcinomas compared to randomly selected variants nearby randomly selected genes. We used variants that were found within 35 kb upstream or downstream of the gene TSS. **f**, rs10759975 is associated with *trans*-eGene *TMEM253*. **g**, rs10759975 is associated with *trans*-eGene *ARFGEF3*. **h**, rs10759975 shows *cis* association with *TRMO*. **i**, rs10759975 is weakly associated *in cis* with *FOXE1*. Box plots depict the IQR, whiskers depict 1.5× IQR.

**Figure 6:**
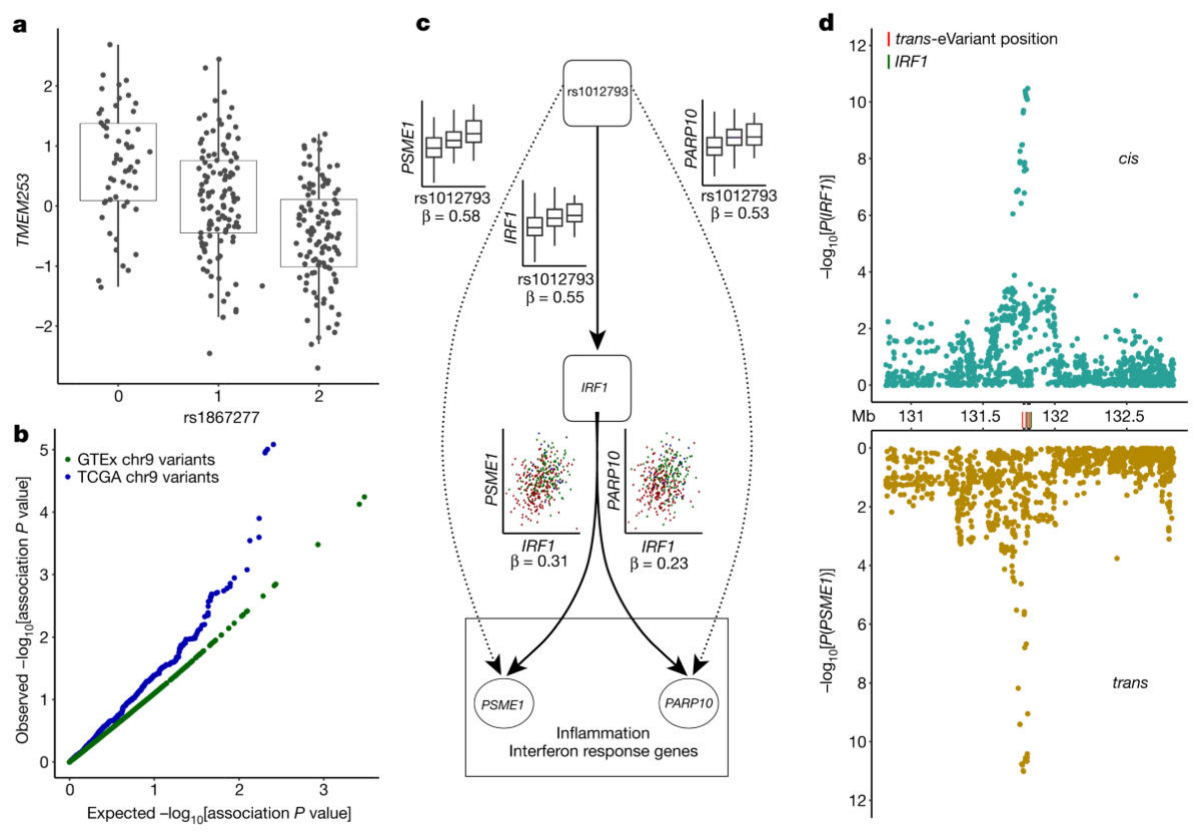
Characterization of complex trait-associated *trans*-eQTLs **a**, Association of rs1867277 with PEER-corrected *TMEM253* expression (*P* ≤ 2.2 ×10^−16^). **b**, Quantile–quantile plot of associations between 19 variants in the 9q22 locus and all genes in GTEx thyroid gene expression levels, compared to 19 random variants from the same chromosome, and associations between 23 variants in the 9q22 locus and all genes in TCGA thyroid tumour expression data, compared to 23 random variants from the same chromosome. **c**, Network depicting *cis* and *trans* regulatory effects of rs1012793 mediated through interferon regulatory factor 1 (IRF1). Rs1012793 affects expression of *IRF1* in *cis* and *PSME1* and *PARP10* in *trans* (box plots). *IRF1* is significantly co-expressed with the *trans*-eGenes. Colours in scatter plots refer to genotype at rs1012793. **d**, *cis* and *trans* association significance of variants within 1 Mb of the *IRF1* TSS in the chromosome 5 locus with *cis*-eGene *IRF1* (blue) and *trans*-eGene *PSME1* (brown), showing concordant signal across the locus. Box plots depict the IQR, whiskers depict 1.5× IQR.

Among trait-associated variants with *trans*-eQTL effects, we found two genome-wide significant *trans*-eVariants at the 9q22 locus (rs7037324 and rs1867277, *R*^2^ = 0.74) with thyroid-specific associations in *trans* with *TMEM253* and *ARFGEF3* (*P* ≤ 2.2 × 10^−16^ for both with rs1867277; Fig. 6a and Extended Data Fig. 16). The 9q22 locus has previously been linked to multiple thyroid-specific diseases including goitre, hypothyroidism and thyroid cancer^48,49^, and loss-of- function mutations in a thyroid-specific transcription factor at this locus, *FOXE1*, manifest as ectopic thyroid tissue or cleft palate in developing mice. However, the mechanism of any *cis*-effects of these *trans*-eVariants remains uncertain from the GTEx data; a post hoc analysis demonstrated that PEER correction removed broad regulatory signals from the 9q22 locus, and particularly from *cis*- and *trans*-eQTL signals for *FOXE1* (Supplementary Information 17). In PEER-corrected data, *cis*- and *trans*-eQTL signals co-localized for another *cis*-eGene in 9q22, *TRMO*, for both *trans*-eGenes^43^ (posterior probability >0.99). Mendelian randomization analysis of the PEER-corrected data supported the idea that *TRMO* regulates *TMEM253* (*P* ≤1.3×10^−9^) and *ARFGEF3* (*P* ≤2.1×10^−11^) based on *trans*-eVariant rs1867277. By contrast, *FOXE1* had weak Mendelian randomization support in the PEER-corrected data. Despite the ambiguity of *cis*-mediation, the locus is one of the strongest *trans*-eQTL signals in GTEx. We further replicated both the broad regulatory effect and specific target genes of this locus in 498 primary thyroid cancer RNA-seq samples from The Cancer Genome Atlas^50^ (TCGA; Fig. 6b, Supplementary Information 18).

**Extended Data Figure 17:**
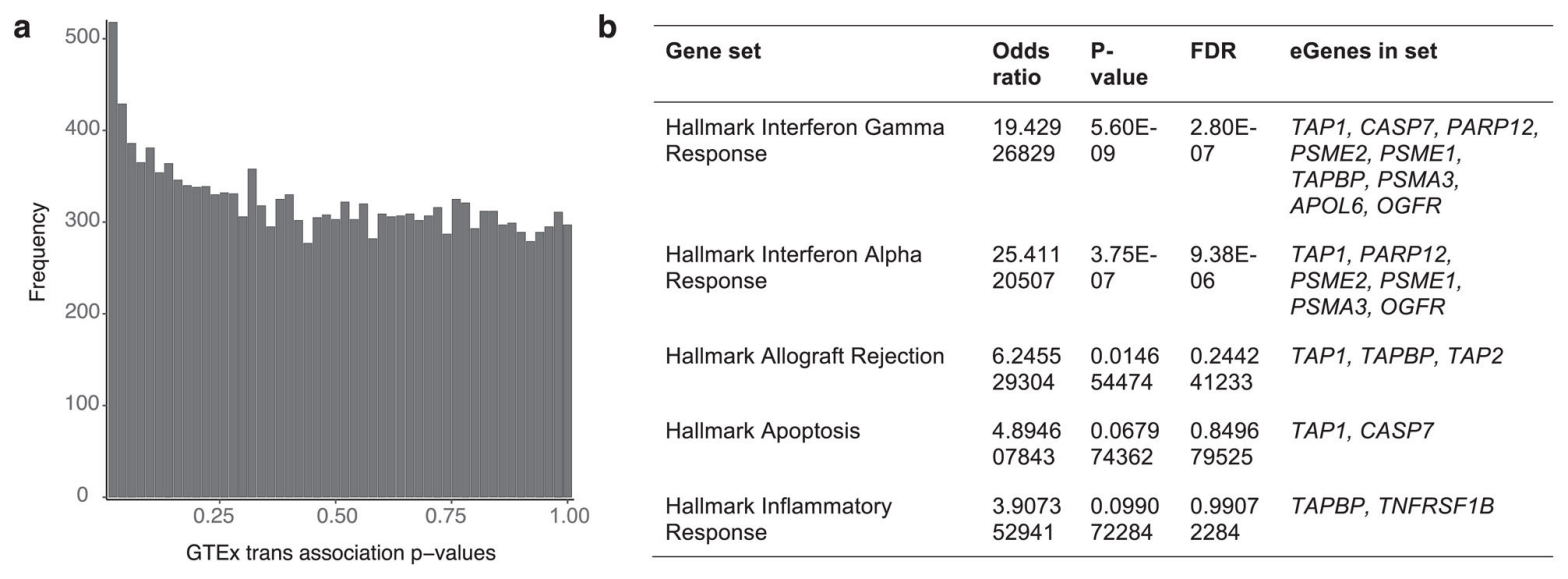
Trait-associated variants in skeletal muscle near interferon regulatory factor *IRF1* **a**, rs1012793 has broad regulatory impact in skeletal muscle. **b**, Gene set enrichment for potential *trans*-eGene targets (identified at *P* ≤ 0.001) of skeletal muscle 5q31 locus.

In a second example, two muscle-specific *trans*-eVariants at the 5q31 locus (rs2706381 and rs1012793; *R*^2^ = 0.84) were associated in *trans* with *PSME1* (*P* ≤1.1×10^−11^) and *PARP10* (*P* ≤7.8×10^−10^), and in *cis* with *IRF1* (*P* ≤2.0×10^−10^; Fig. 6c), a transcription factor that facilitates regulation of the interferon-induced immune response^51,52^. Both variants are associated with circulating fibrinogen levels^53^ and influence muscle injury, Duchenne muscular dystrophy (DMD), multiple sclerosis and rheumatoid arthritis^54,55^, and have been shown to drive fibrosis in DMD, where they promote expression of *IL1B* and *TGFB1*^56^. These variants were moderately associated with numerous genes in skeletal muscle (50 *trans*-eGenes at 20% FDR, assessed only among the three variants; Extended Data Fig. 17a). Additional candidate target genes (at 20% FDR) were enriched in multiple immune pathways from MsigDB^57^ (Extended Data Fig. 17b). Mendelian randomization analysis supported the idea that *IRF1* regulates *PSME1* (*P* ≤3.1×10^−8^) and *PARP10* (*P* ≤1.9×10^−7^) through *cis*-eVariant rs2706381 with a consistent direction of effect (Fig. 6c). Moreover, the *cis*-eQTL signal for *IRF1* co-localized with the *trans*-eQTL signals for both *trans*-eGenes (Fig. 6d; posterior probability >0.99)^43^. Together, these results suggest that *cis*-regulatory loci affecting *IRF1* are regulators of interferon-responsive inflammatory processes involving genes including *PSME1* and *PARP10*, with implications for complex traits specific to muscle tissue.

## Discussion

Since the initial sequencing of the human genome, extensive effort has been devoted to the characterization of genome function and phenotypic consequences of genetic variation. Describing the effects of genetic variation on gene expression levels across tissues is a critical but challenging component of this goal. Here, we describe advances enabled by the GTEx project v6p data, which provide a comprehensive survey of gene expression and the impact of genetic variation on gene expression across diverse human tissues. We report widespread *cis*-eQTLs in 44 tissues and *trans*-eQTLs in 18 tissues. *cis*-acting genetic variants tend to affect either most tissues or a small number of tissues. By contrast, identified *trans*-eQTL effects tend to be tissue-specific and correspondingly show greater enrichment in enhancer regions. By integrating GTEx data with summary statistics from diverse GWAS, we observed that half of complex trait- associated loci co-localize with a GTEx eQTL. GTEx data have already served as a valuable community resource for the identification of the tissue-specific regulatory effects underlying variants associated with human disease phenotypes^58–61^.

Additional papers from the GTEx consortium for the v6p data describe the impact of rare genetic variation on gene expression^62^, methods for analysis of transcriptome data^27^, the discovery and characterization of regulatory networks across tissues^63^, and analyses of diverse regulatory processes such as RNA editing^64^ and X-inactivation^65^. To enable ongoing use of the GTEx data, summary-level expression data and eQTLs across all tissues are available from the GTEx Portal (www.gtexportal.org), while all individual-level raw data have been deposited in dbGaP (accession phs000424.v6.p1).

There are both opportunities and challenges as efforts to characterize genome function grow in scope and scale. The discovery and characterization of eQTLs in these data required careful data modelling to account for confounders and to characterize statistical discovery. We anticipate that complementary analyses with novel methods, enabled by the public availability of these data, may reveal additional insights. Despite the scope of these data, we remain underpowered to detect *trans*-eQTLs. Larger cohorts of individuals with a smaller number of tissues have yielded hundreds of *trans*-eGenes^4,6,8,9^, and we similarly expect *trans*-eQTL discoveries to increase with additional samples in the final phase of GTEx. Furthermore, some genetic effects may manifest only within a specific cell type, rather than an entire heterogeneous tissue. Both computational and experimental methods, such as deconvolution methods and single-cell sequencing as part of the proposed Human Cell Atlas and related projects, promise to improve resolution to identify precise cell type-specific regulatory effects^66^. Future aims of the GTEx project include increased sample size, with *cis*-eQTLs from 53 tissues across 714 donors, now available in the v7 release, and plans to include approximately 1,000 donors in the final data release. Additional plans include the collection of complementary molecular data on subsets of samples, including epigenetic and protein data, with the Enhanced GTEx (eGTEx) project, enabling an increasingly complete picture of epigenetic and regulatory variant diversity across human tissues^67^. We expect that the continued expansion of the GTEx resource, and its integration with other efforts capturing diverse data types, will be an essential asset for the study of gene regulatory mechanisms and how these contribute to human traits and diseases.

## METHODS

No statistical methods were used to predetermine sample size. The experiments were not randomized, and investigators were not blinded to allocation during experiments and outcome assessment.

### Sample procurement

All human donors were deceased. Informed consent was obtained for all donors via next-of-kin consent to permit the collection and banking of de-identified tissue samples for scientific research. The research protocol was reviewed by Chesapeake Research Review Inc., Roswell Park Cancer Institute’s Office of Research Subject Protection, and the institutional review board of the University of Pennsylvania. Complete descriptions of the donor enrolment and consent process, as well as biospecimen procurement, methods, sample fixation, and histopathological review procedures, have been described previously^10,67^. In brief, whole blood was collected from each donor, along with fresh skin samples, for DNA genotyping, RNA expression, and culturing of lymphoblastoid and fibroblast cells, and shipped overnight to the GTEx Laboratory Data Analysis and Coordination Center (LDACC) at the Broad Institute. Two adjacent aliquots were then prepared from each sampled tissue and preserved in PAXgene tissue kits. One of each paired sample was embedded in paraffin (PFPE) for histopathological review and the second was shipped to the LDACC for processing and molecular analysis. Brains were collected from approximately one-third of the donors, and were shipped on ice to the brain bank at the University of Miami, where eleven brain sub-regions were sampled and flash-frozen. These samples were also shipped to the LDACC for processing and analysis.

All DNA genotyping was performed on blood-derived DNA samples, unless these were unavailable, in which case a tissue-derived DNA sample was substituted. RNA was extracted from all tissues and RNA sequencing was performed on all samples with an RNA integrity number (RIN) of 5.7 or higher and with at least 500 ng total RNA. Nucleic acid isolation protocols and sample QC metrics applied are as described^10^ (Supplementary Information 1–5).

### Data production

Non-strand specific, polyA^+^ selected RNA-seq libraries were generated using the Illumina TruSeq protocol. Libraries were sequenced to a median depth of 78 million 76-bp paired-end reads. RNA-seq reads were aligned to the human genome (hg19/GRCh37) using TopHat (v1.4) based on GENCODE v19 annotations. This annotation is available on the GTEx Portal (gencode.v19. genes.v6p_model.patched_contigs.gtf.gz, available at https://www.gtexportal.org/home/datasets). Gene-level expression was estimated as reads per kilobase of transcript per million mapped reads (RPKM) using RNA-SeQC on uniquely mapped, properly paired reads fully contained within exon boundaries and with alignment distances ≤ 6. Samples with fewer than 10 million mapped reads or with outlier expression measurements based on the *D* statistic were removed^10^.

DNA from 450 donors was genotyped using Illumina Human Omni 2.5M and 5M Beadchips. Genotypes were phased and imputed with SHAPEIT2^68^ and IMPUTE2^69^, respectively, using multi-ethnic panel reference from 1000 Genomes Project Phase 3^70^. Variants were excluded from analysis if they: 1) had a call rate < 95%; 2) had minor allele frequencies < 1%; 3) deviated from Hardy–Weinberg equilibrium (*P* <1.0×10^−6^); or 4) had an imputation info score <0.4. The final genotyped and imputed array VCF (file format v4.1) for autosomal variants contains genotype posterior probabilities for each of the three possible genotypes for 11,552,519 variants across 450 GTEx donors. The dosages of the alternative alleles relative to the human reference genome hg19 were used as the genotype measure for subsequent eQTL analysis. In addition to array-based genotyping, 148 and 524 donors were whole-genome and exome sequenced, respectively. Additional details on genotyping, imputation and sequencing can be found in the Supplementary Information.

### *cis*-eQTL mapping

We conducted *cis*-eQTL mapping within the 44 tissues with at least 70 samples each. Only genes with ten or more donors with expression estimates > 0.1 RPKM and an aligned read count of six or more within each tissue were considered significantly expressed and used for *cis*-eQTL mapping. Within each tissue, the distribution of RPKMs in each sample was quantile-transformed using the average empirical distribution observed across all samples. Expression measurements for each gene in each tissue were subsequently transformed to the quantiles of the standard normal distribution. The effects of unobserved confounding variables on gene expression were quantified with PEER^12^, run independently for each tissue. Fifteen PEER factors were identified for tissues with fewer than 150 samples; 30 for tissues with sample sizes between 150 and 250; and 35 for tissues with more than 250 tissues. The covariates that were most consistently associated with PEER factors include factors related to parameters of donor death, ischaemic time, RIN and sequencing quality control metrics. In addition, we have observed that little, if any, genetic signal is present in the PEER factors (Supplementary Information 6).

Within each tissue, *cis*-eQTLs were identified by linear regression, as implemented in FastQTL^71^, adjusting for PEER factors, sex, genotyping platform, and three genotype-based principal components (PCs). We restricted our search to variants within 1 Mb of the TSS of each gene and, in the tissue of analysis, minor allele frequencies ≥0.01 with the minor allele observed in at least 10 samples. Nominal *P* values for each variant–gene pair were estimated using a two-tailed *t*-test. The significance of the most highly associated variant per gene was determined from empirical *P* values, extrapolated from a Beta distribution fitted to adaptive permutations with the setting –permute 1000 10000. These empirical *P* values were subsequently corrected for multiple testing across genes using Storey’s *q* value method^16^. To identify the list of all significant variant–gene pairs associated with eGenes, variants with a nominal *P* value below the gene-level threshold were considered significant and included in the final list of variant–gene pairs.

### *trans*-eQTL mapping

Matrix eQTL^72^ was used to test all autosomal variants (MAF > 0.05) using the same expression filters as *cis*-eQTL mapping, but restricted to variants and genes lying on different chromosomes, in each tissue independently using an additive linear model. For *trans*-eQTL mapping, we tested variants for association with expression of only protein coding or lincRNA genes. We included as covariates the three genotype PCs, genotyping platform, sex, and PEER factors estimated from expression data in Matrix eQTL when performing association testing. The correlation between variant and gene expression levels was evaluated using the estimated *t* statistic from this model, and corresponding FDR was estimated using Benjamini–Hochberg FDR correction^72,73^ separately within each tissue and also using permutation analysis. We performed restricted *trans*-eQTL association tests by filtering the set of variants considered in three ways. First, we filtered the final VCF files using linkage disequilibrium pruning (*R*^2^ > 0.5, plink parameters – indep 50 5 2), removing approximately 90% of variants. Second, from the original VCF file, we performed association mapping using only the most significant GTEx *cis*-eQTL per eGene per tissue. Third, from the original VCF file, we performed association mapping using only variants that had been found to have a trait association in a genome-wide association study^47^ (*P* ≤2.0×10^−5^). The three association mapping analyses and FDR estimation were performed in each tissue separately. For all *trans* association tests, we applied stringent quality control to account for potential false positives due to RNA-seq read mapping errors, repeat elements, and population stratification (Supplementary Information 7).

### Multi-tissue eQTL mapping

We quantified the tissue-specificity and tissue-sharing of *cis*- and *trans*-eQTLs using Meta-Tissue^15^. This tool extends Metasoft^74^, a meta-analysis package, by using a mixed effects model for eQTL sharing that accounts for correlation of expression between tissues driven by overlapping donors. All genotypes and gene expression quantification estimates were adjusted for covariates in accordance to the single tissue analysis as described in the previous sections. For each variant–gene pair, we calculated mixed model effect size estimates in each expressed tissue, thereby adjusting for partial sharing of signal between tissues. These effect size estimates were used in meta-analysis using Metasoft^74^ to assess the tissue-specificity of each variant–gene pair. For each variant–gene pair tested, Meta-Tissue estimates a global *P* value of association and the posterior probability that an effect exists in a tissue (*m* value). For computational feasibility, the Markov chain Monte Carlo (MCMC) method was used to approximate the exact solution.

Hierarchical agglomerative clustering was performed on *trans*-eGenes (50% FDR) and *cis*-eGenes (5% FDR) using distance metric (1 − Spearman’s *ρ*) of Meta-Tissue effect sizes across all observed genes between tissue pairs. To supplement this analysis, we also performed multi-tissue analysis using 1) replication analysis (Extended Data Fig. 7); 2) hierarchical FDR control^17^ for both *cis* and *trans* analysis (Supplementary Information 8); and 3) an empirical Bayes approach^18^.

### Allele-specific expression

For each sample, allele-specific RNA-seq read counts were generated at all heterozygous variants with the GATK ASEReadCounter tool^75^. Only uniquely mapping reads with a base quality ≥10 at the variant were counted, and only those variants with coverage of at least eight reads were reported. Variants that met any of the following criteria were flagged and removed from downstream analyses: 1) UCSC 50-mer mappability of <1; 2) simulation-based evidence of mapping bias^76^; and 3) heterozygous genotype not supported by RNA-seq data across all samples for that donor and no significant (FDR > 1%) evidence that the variant is monoallelic in expression data^75^. Gene level measurements of haplotype expression were calculated by aggregating counts per sample across all heterozygous variants with ASE data within the gene using population phasing. Full ASE data are available through dbGaP.

### Functional enrichment

We annotated discovered eVariants using chromatin state predictions from 128 cell types or cell lines sampled by the Roadmap Epigenomics project^2^. Genome segmentation was performed for each cell type or cell line using a 15-state hidden Markov model (HMM) over 400 bp windows. Several of the learned states are labelled as enhancers, promoters, and repressed regions. For the standard 15-state Roadmap segmentations, regulatory elements are labelled independently for each cell type. For enrichment analyses, we constructed background variants sets that matched eVariants to randomly selected variants based on chromosome, distance to nearest TSS, and MAF.

*trans*-eQTL analysis was restricted to protein-coding genes and to GTEx tissues that are composed of at least one Roadmap Epigenomics cell type (26 tissues), which included 85 eVariants and 23 eGenes (10% FDR). We quantified enrichment of the *trans* variants relative to random variants in both enhancer and promoter elements in the GTEx discovery tissue’s matched Roadmap cell type (Extended Data Table 4). We then performed the same analysis with randomly matched *cis*-eGenes. Matching *cis*-eGenes were selected as follows: for each of the 23 *trans*-eGenes *g*, each having *N_g_* associated eVariants (10% FDR), we randomly selected a *cis*-eGene that also had at least *N_g_* associated variants (10% FDR). We then selected the top *N_g_* variants associated with this gene based on *P* value. We then performed the same analysis using random sets of the strongest *cis*-eGenes, rather than random eGenes. Matching the strongest *cis*-eGenes was performed as follows: for each of the 23 *trans*-eGenes *g*, each having *N_g_* associated eVariants (10% FDR), we randomly selected a *cis*-eGene amongst the ten strongest *cis*-eGenes in that tissue, based on the *P* value of the strongest associated variant that also had at least *N_g_* associated variants (10% FDR). We then selected the top *N_g_* associated variants with this gene based on *P* value. Selecting 23 random *cis*-eGenes a single time yields unstable results, so we ran *cis*-eGene selection and enrichment 70 times with different selections. This was done for both random *cis*-eGenes and random selections amongst the strongest *cis*-eGenes. We rank-ordered the 70 trials for both promoters and enhancers based on average odds ratio enrichment relative to background. We then used the trial that was closest to median rank for plotting both promoter and enhancer enrichment results.

For piRNA enrichment analysis, we obtained a list of 6,250 piRNA clusters that were experimentally determined from RNA sequencing of human testis^34^. When considering all unique *trans*-eVariants identified in all tissues, we identified an enrichment of *trans*-eQTLs overlapping a piRNA cluster (17.8%) compared to the null expectation that *trans*-eVariants are randomly distributed relative to piRNA clusters (2.5%). To further establish the statistical significance of this observation, we generated a null distribution of piRNA-eVariant overlap by permutation. Using bedtools2^77^, we permuted the location of piRNA clusters on the human genome 10,000 times, requiring the piRNA clusters to be excluded from centromeres and sex chromosomes. We also evaluated the proportion of *trans*-eVariants located within 10 kb of a piRNA cluster, and estimated the significance of this enrichment using the same permutation scheme.

### Co-localization of GWAS and eQTL associations

In order to assess the probability that molecular traits as estimated by *cis*- and *trans*-eQTLs and physiological traits as estimated by GWAS share the same causal variant, we applied the coloc R package^43^. For each GWAS, we approximated the number of independent loci by extracting variants with at least genome-wide significance (*P* <5 ×10^−8^) and farther than 1 Mb away from all other variants of higher statistical significance. For each genome-wide significant variant, we extracted the list of all eGenes (*q* < 0.05 for *cis*-eGene) within 1 Mb for coloc analyses. For each eGene, we excluded any variants without either eQTL or GWAS association statistics (effect size estimate, standard error and *P* value). We obtained reference information such as MAF, sample size and case-to-control proportions (in case of binary traits) for each variant whenever available; otherwise, study-wide estimate was used as a proxy. We defined a region or an eGene as having evidence of co-localization when region- or gene-based posterior probability of co-localization $\frac{\text{PP}4}{\text{PP}3+\text{PP}4}>0.9$.

### Data and biospecimen availability

Genotype data from the GTEx v6p release are available in dbGaP (study accession phs000424.v6.p1; https://www.ncbi.nlm.nih.gov/projects/gap/cgi-bin/study.cgi?study_id=phs000424.v6.p1). The VCFs for the imputed array data are available through dgGAP, in phg000520.v2.GTEx MidPoint Imputation.genotype-calls-vcf.c1.GRU.tar (the archive contains a VCF for chromosomes 1–22 and a VCF for chromosome X). Allelic expression data are also available in dbGaP. Expression data (read counts and RPKM) and eQTL input files (normalized expression data and covariates for 44 the tissues) from the GTEx v6p release are available from the GTEx Portal (http://gtexportal.org). Expression QTL results are available from the GTEx Portal. In addition to results tables for the 44 tissues in this study (eGenes, significant variant–gene pairs, and all variant–gene pairs tested), the portal provides multiple interactive visualization and data exploration features for eQTLs, including:

- eQTL box plot: displays variant-gene associations
- Gene eQTL visualizer: displays all significant associations for a gene across tissues and linkage disequilibrium information
- Multi-tissue eQTL plot: displays multi-tissue posterior probabilities from meta-analysis against single-tissue association results
- IGV browser: displays eQTL, across tissues and GWAS catalogue results for a selected genomic region

Residual biospecimens are available to all researchers according to the Genotype-Tissue Expression (GTEx) project biospecimens access policy. The policy and related forms can be found on the GTEx Portal under the Biobank tab.

### Code availability

Software used to process the RNA-seq, genotypes, *cis*-eQTLs, and ASE is available at: https://github.com/broadinstitute/gtex-pipeline.

## Extended Data

**Extended Data Table 1:** *trans*-eVariant and *trans*-eGene discoveries for genome-wide FDr control, and *trans*-eGene discoveries for gene-level FDr control

		Genome wide		Gene-level FDR

Tissue	No. of samples	No. of trans-eGenes	No. of trans-eVariants	No. of trans-eGenes
Muscle – Skeletal	361	9	43	4
Whole Blood	338	1	2	1
Skin – Sun Exposed (Lower leg)	302	6	16	3
Adipose – Subcutaneous	298	2	7	0
Lung	278	2	2	2
Thyroid	278	21	181	3
Cells – Transformed fibroblasts	272	1	10	1
Nerve – Tibial	256	0	0	1
Esophagus – Mucosa	241	3	11	3
Artery – Aorta	197	1	1	1
Skin – Not Sun Exposed (Suprapubic)	196	1	1	2
Stomach	170	0	0	2
Colon – Transverse	169	2	10	2
Testis	157	35	267	16
Pancreas	149	2	12	1
Adrenal Gland	126	1	1	1
Brain – Putamen (Basal ganglia)	82	3	11	2
Vagina	79	4	27	1

Total unique		93	602	46

Each tissue with non-zero values is included as a row; the columns include the number of samples for that tissue, followed by the number of unique *trans*-eGenes and *trans*-eVariants identified in the genome-wide tests, and the number of unique *trans*-eGenes found using gene-level FDR calibration (Supplementary Information 8). Ultimately, the set of 673 *trans*-eQTLs identified in the genome-wide approach yielded 602 unique *trans*-eVariants.

**Extended Data Table 2:** Distal (>5 Mb) intra-chromosomal eQTLs across the GTEx tissues

Tissue	No. of samples	No. of trans-eGenes	No. of trans-eVariants
Whole Blood	338	4	17
Skin – Sun Exposed (Lower leg)	302	2	12
Adipose – Subcutaneous	297	1	2
Lung	278	2	37
Thyroid	278	5	17
Cells – Transformed fibroblasts	272	4	17
Esophagus – Mucosa	241	15	184
Testis	157	4	30
Brain – Putamen (Basal ganglia)	82	1	2
Brain – Hypothalamus	81	1	2

Total unique	7047	33	284

Only tissues with at least one distal intra-chromosomal eQTL are listed.

**Extended Data Table 3:** *trans*-eVariant and *trans*-eGene discoveries with hierarchical FDr control

Tissue	No. of samples	No. of trans-eGenes	No. of trans-eVariants
Whole Blood	338	1	1
Skin – Sun Exposed (Lower leg)	302	2	3
Lung	278	2	2
Thyroid	278	2	2
Esophagus – Mucosa	241	3	3
Artery – Aorta	197	1	1
Skin – Not Sun Exposed (Suprapubic)	196	1	1
Heart – Left Ventricle	190	1	1
Testis	157	4	5
Colon – Sigmoid	124	1	1
Brain – Cortex	96	1	1
Brain – Putamen (Basal ganglia)	82	1	1

Total unique		20	22

Only tissues with non-zero discoveries are shown. The three-level hierarchical procedure (see Methods) performs FDR control across tissues. More specifically, it controls the FDR of eVariants, the average proportion of false variant-gene associations across all eVariants, and a weighted average of false tissue discoveries for the selected variant-gene pairs (weighted by the size of the eVariant and eGene sets). The procedure was applied after linkage disequilibrium pruning.

**Extended Data Table 4:** GTEx tissue mapping with Epigenomics roadmap cell types

GTEx Tissue	Epigenomics Roadmap Cell Type
Adipose – Subcutaneous	Adipose Nuclei (E063)
Adipose – Visceral (Omentum)	Adipose Nuclei (E063)
Adrenal Gland	NA
Artery – Aorta	Aorta (E065)
Artery – Coronary	NA
Artery – Tibial	NA
Brain – Anterior cingulate cortex (BA24)	Brain Cingulate Gyrus (E069)
Brain – Caudate (basal ganglia)	Brain Anterior Caudate (E068)
Brain – Cerebellar Hemisphere	NA
Brain – Cerebellum	NA
Brain – Cortex	Brain Angular Gyrus (E067), Brain Inferior Temporal Lobe (E072), Brain Dorsolateral Prefrontal Cortex (E073)
Brain – Frontal Cortex (BA9)	Brain Inferior Temporal Lobe (E072), Brain – Dorsolateral Prefrontal Cortex (E073)
Brain – Hippocampus	Brain Hippocampus Middle (E071)
Brain – Hypothalamus	NA
Brain – Nucleus accumbens (basal ganglia)	NA
Brain – Putamen (basal ganglia)	NA
Breast – Mammary Tissue	Breast Myoepithelial Primary Cells (E027)
Cells – EBV-transformed lymphocytes	Lymphoblastoid Cells (E116)
Cells – Transformed fibroblasts	NA
Colon – Sigmoid	Sigmoid Colon (E106)
Colon – Transverse	Colonic Mucosa (E075), Colon Smooth Muscle (E076)
Esophagus – Gastroesophageal Junction	Esophagus (E079)
Esophagus – Mucosa	Esophagus (E079)
Esophagus – Muscularis	Esophagus (E079)
Heart – Atrial Appendage	Right Atrium (E104)
Heart – Left Ventricle	Left Ventricle (E095)
Liver	Liver (E066)
Lung	Lung (E096)
Muscle – Skeletal	Skeletal Muscle Male (E107), Skeletal Muscle Female (E108)
Nerve – Tibial	NA
Ovary	Ovary (E097)
Pancreas	Pancreas (E098)
Pituitary	NA
Prostate	NA
Skin – Not Sun Exposed (Suprapubic)	NA
Skin – Sun Exposed (Lower leg)	NA
Small Intestine – Terminal Ileum	Small Intestine (E109)
Spleen	Spleen (E113)
Stomach	Stomach Mucosa (E110), Stomach Smooth Muscle (E111)
Testis	NA
Thyroid	NA
Uterus	NA
Vagina	NA
Whole Blood	Primary mononuclear cells from peripheral blood (E062)

## Supplementary Material

S1.pdf

S2.pdf

S3.xlsx

S4.xlsx

## GTEx Consortium

### Laboratory, Data Analysis & Coordinating Center (LDACC)—Analysis Working Group

François Aguet^1^, Kristin G. Ardlie^1^, Beryl B. Cummings^1,2^, Ellen T. Gelfand^1^, Gad Getz^1,3^, Kane Hadley^1^, Robert E. Handsaker^1,4^, Katherine H. Huang^1^, Seva Kashin^1,4^, Konrad J. Karczewski^1,2^, Monkol Lek^1,2^, Xiao Li^1^, Daniel G. MacArthur^1,2^, Jared L. Nedzel^1^, Duyen T. Nguyen^1^, Michael S. Noble^1^, Ayellet V. Segrè^1^, Casandra A. Trowbridge^1^, Taru Tukiainen^1,2^

### Statistical Methods groups—Analysis Working Group

Nathan S. Abell^5,6^, Brunilda Balliu^6^, Ruth Barshir^7^, Omer Basha^7^, Alexis Battle^8^, Gireesh K. Bogu^9,10^, Andrew Brown^11,12,13^, Christopher D. Brown^14^, Stephane E. Castel^15,16^, Lin S. Chen^17^, Colby Chiang^18^, Donald F. Conrad^19,20^, Nancy J. Cox^21^, Farhan N. Damani^8^, Joe R. Davis^5,6^, Olivier Delaneau^11,12,13^, Emmanouil T. Dermitzakis^11,12,13^, Barbara E. Engelhardt^22^, Eleazar Eskin^23,24^, Pedro G. Ferreira^25,26^, Laure Frésard^5,6^, Eric R. Gamazon^21,27,28^, Diego Garrido-Martín^9,10^, Ariel D.H. Gewirtz^29^, Genna Gliner^30^, Michael J. Gloudemans^5,6,31^, Roderic Guigo^9,10,32^, Ira M. Hall^18,19,33^, Buhm Han^34^, Yuan He^35^, Farhad Hormozdiari^23^, Cedric Howald^11,12,13^, Hae Kyung Im^36^, Brian Jo^29^, Eun Yong Kang^23^, Yungil Kim^8^, Sarah Kim-Hellmuth^15,16^, Tuuli Lappalainen^15,16^, Gen Li^37^, Xin Li^6^, Boxiang Liu^5,6,38^, Serghei Mangul^23^, Mark I. McCarthy^39,40,41^, Ian C. McDowell^42^, Pejman Mohammadi^15,16^, Jean Monlong^9,10,43^, Stephen B. Montgomery^5,6^, Manuel Muñoz-Aguirre^9,10,44^, Anne W. Ndungu^39^, Dan L. Nicolae^36,45,46^, Andrew B. Nobel^47,48^, Meritxell Oliva^36,49^, Halit Ongen^11,12,13^, John J. Palowitch^47^, Nikolaos Panousis^11,12,13^, Panagiotis Papasaikas^9,10^, YoSon Park^14^, Princy Parsana^8^, Anthony J. Payne^39^, Christine B. Peterson^50^, Jie Quan^51^, Ferran Reverter^9,10,52^, Chiara Sabatti^53,54^, Ashis Saha^8^, Michael Sammeth^55^, Alexandra J. Scott^18^, Andrey A. Shabalin^56^, Reza Sodaei^9,10^, Matthew Stephens^45,46^, Barbara E. Stranger^36,49,57^, Benjamin J. Strober^35^, Jae Hoon Sul^58^, Emily K. Tsang^6,31^, Sarah Urbut^46^, Martijn van de Bunt^39,40^, Gao Wang^46^, Xiaoquan Wen^59^, Fred A. Wright^60^, Hualin S. Xi^51^, Esti Yeger-Lotem^7,61^, Zachary Zappala^5,6^, Judith B. Zaugg^62^, Yi-Hui Zhou^60^

### Enhancing GTEx (eGTEx) groups

Joshua M. Akey^29,63^, Daniel Bates^64^, Joanne Chan^5^, Lin S. Chen^17^, Melina Claussnitzer^1,65,66^, Kathryn Demanelis^17^, Morgan Diegel^64^, Jennifer A. Doherty^67^, Andrew P. Feinberg^35,68,69,70^, Marian S. Fernando^36,49^, Jessica Halow^64^, Kasper D. Hansen^68,71,72^, Eric Haugen^64^, Peter F. Hickey^72^, Lei Hou^1,73^, Farzana Jasmine^17^, Ruiqi Jian^5^, Lihua Jiang^5^, Audra Johnson^64^, Rajinder Kaul^64^, Manolis Kellis^1,73^, Muhammad G. Kibriya^17^, Kristen Lee^64^, Jin Billy Li^5^, Qin Li^5^, Xiao Li^5^, Jessica Lin^5,74^, Shin Lin^5,75^, Sandra Linder^5,6^, Caroline Linke^36,49^, Yaping Liu^1,73^, Matthew T. Maurano^76^, Benoit Molinie^1^, Stephen B. Montgomery^5,6^, Jemma Nelson^64^, Fidencio J. Neri^64^, Meritxell Oliva^36,49^, Yongjin Park^1,73^, Brandon L. Pierce^17^, Nicola J. Rinaldi^1,73^, Lindsay F. Rizzardi^68^, Richard Sandstrom^64^, Andrew Skol^36,49,57^, Kevin S. Smith^5,6^, Michael P. Snyder^5^, John Stamatoyannopoulos^64,74,77^, Barbara E. Stranger^36,49,57^, Hua Tang^5^, Emily K. Tsang^6,31^, Li Wang^1^, Meng Wang^5^, Nicholas Van Wittenberghe^1^, Fan Wu^36,49^, Rui Zhang^5^

### NIH Common Fund

Concepcion R. Nierras^78^

### NIH/NCI

Philip A. Branton^79^, Latarsha J. Carithers^79,80^, Ping Guan^79^, Helen M. Moore^79^, Abhi Rao^79^, Jimmie B. Vaught^79^

### NIH/NHGrI

Sarah E. Gould^81^, Nicole C. Lockart^81^, Casey Martin^81^, Jeffery P. Struewing^81^, Simona Volpi^81^

### NIH/NIMH

Anjene M. Addington^82^, Susan E. Koester^82^

### NIH/NIDA

A. Roger Little^83^

### Biospecimen Collection Source Site—NDrI

Lori E. Brigham^84^, Richard Hasz^85^, Marcus Hunter^86^, Christopher Johns^87^, Mark Johnson^88^, Gene Kopen^89^, William F. Leinweber^89^, John T. Lonsdale^89^, Alisa McDonald^89^, Bernadette Mestichelli^89^, Kevin Myer^86^, Brian Roe^86^, Michael Salvatore^89^, Saboor Shad^89^, Jeffrey A. Thomas^89^, Gary Walters^88^, Michael Washington^88^, Joseph Wheeler^87^

### Biospecimen Collection Source Site—rPCI

Jason Bridge^90^, Barbara A. Foster^91^, Bryan M. Gillard^91^, Ellen Karasik^91^, Rachna Kumar^91^, Mark Miklos^90^, Michael T. Moser^91^

### Biospecimen Core resource—VArI

Scott D. Jewell^92^, Robert G. Montroy^92^, Daniel C. Rohrer^92^, Dana R. Valley^92^

### Brain Bank repository—University of Miami Brain Endowment Bank

David A. Davis^93^, Deborah C. Mash^93^

### Leidos Biomedical—Project Management

Anita H. Undale^94^, Anna M. Smith^95^, David E. Tabor^95^, Nancy V. Roche^95^, Jeffrey A. McLean^95^, Negin Vatanian^95^, Karna L. Robinson^95^, Leslie Sobin^95^, Mary E. Barcus^96^, Kimberly M. Valentino^95^, Liqun Qi^95^, Steven Hunter^95^, Pushpa Hariharan^95^, Shilpi Singh^95^, Ki Sung Um^95^, Takunda Matose^95^, Maria M. Tomaszewski^95^

### ELSI Study

Laura K. Barker^97^, Maghboeba Mosavel^98^, Laura A. Siminoff^97^, Heather M. Traino^97^

### Genome Browser Data Integration & Visualization—EBI

Paul Flicek^99^, Thomas Juettemann^99^, Magali Ruffier^99^, Dan Sheppard^99^, Kieron Taylor^99^, Stephen J. Trevanion^99^, Daniel R. Zerbino^99^

### Genome Browser Data Integration & Visualization—UCSC Genomics Institute, University of California Santa Cruz

Brian Craft^100^, Mary Goldman^100^, Maximilian Haeussler^100^, W. James Kent^100^, Christopher M. Lee^100^, Benedict Paten^100^, Kate R. Rosenbloom^100^, John Vivian^100^, Jingchun Zhu^100^
