# Supplementary material for: Genetic effects on gene expression across human tissues
Source: Nature. Author manuscript; Available in PMC 2018 Jan 22. (PMC5776756; doi:10.1038/nature24277)
Supplement: S1.pdf [file NIHMS925370-supplement-S1_pdf.pdf]

doi:10.1038/nature24277

Genetic effects on gene expression across human tissues.

The GTEx Consortium

1 Biospecimen Collection and Processing

1.1 Biospecimen Collection

All biospecimens were collected as described in detail in [1] (see Supplemental Information of that publication). Complete descriptions of the donor enrollment and consent process, as well as biospecimen procurement methods, sample fixation, and histopathological review procedures are described in [2]. In brief, a robust quality management program was established and implemented for data management, Standard Operating Procedure (SOP) development, and auditing of collections. Document control software was used to ensure all biospecimen collection sites used current versions of SOPs, and training was conducted prior to implementation of all new procedures. Supporting quality documents were developed to provide consistency and clarity to the program, and many of those documents, such as the SOPs used and workflows for the project, are available to the public (`http://biospecimens.cancer.gov/resources/sops/default.asp`).

1.2 Molecular Analyte Extraction and QC

Detailed protocols for the extraction of DNA and RNA from blood, cell pellets, and PAXgene-fixed and frozen tissues were described in [1]. The same protocols were used to avoid introduction of batch effects among samples, which were processed continually throughout the project. To control for variable RNA quality [1], RNA sequencing was only performed for samples with a RIN score of 5.7 or higher and with at least 500 ng of total RNA.

2 Genotyping, Imputation and Phasing

2.1 Genotyping arrays and sample quality control

455 donors (296 males and 159 females) were genotyped with Illumina Omni arrays for GTEx Release v6 (dbGaP accession phs000424.v6.p1). These include an initial batch of 183 donors genotyped on Illumina's HumanOmni5-Quad Array (4,276,680 variants), and 272 donors genotyped on Illumina's HumanOmni2.5-Quad Array (2,378,075 variants). From the 455 donors, 5 were removed following sample quality control (QC), yielding 450 post-QC donors for eQTL analyses. Three donors were identified as members of a trio or pair of related donors, respectively; one was identified as having Klinefelter syndrome; and one was identified as having chromosome 17p trisomy (**Supplementary Table 1**; the chr17p trisomy donor was detected and excluded after variant QC and imputation; hence these steps were performed on 451 donors). The original genotype calls for all 455 donors are available in dbGaP (phs000424.v3.p1 for the VCF of Omni 5M samples; phs000424.v6.p1 for the VCF of Omni 2.5M samples).

DNA isolated from blood samples was the primary source of DNA used for genotyping ($>$ 360 ng DNA), performed at the Broad Institute of Harvard and MIT. Genotypes were called using Illumina's GeneTrain calling algorithm (Autocall). The genotyping call rates per donor exceeded 98% for all samples. All genotypes and analyses were aligned to chromosome positions from the human reference genome GRCh37/hg19.

To merge the genotypes from Illumina's Omni 5M and Omni 2.5M arrays, we extracted the genotype calls of an overlapping subset of ~2.2 million variants between the two platforms from all samples, using VCFtools (`http://vcftools.sourceforge.net/`). This enabled imputation of the same set of variants in all samples, with minimal loss of accuracy due to high concordance between hard calls and imputed genotypes (**Supplementary Table 2**).

2.2 Variant quality control (QC) of autosomal genotypes before imputation

Multiple sample and variant QC steps were performed before imputation to ensure inclusion of only high confidence variants for eQTL analyses. **Supplementary Table 1** summarizes the QC steps performed using PLINK [3], together with the number of samples and variants removed or retained at each step. This resulted in a set of 1,883,274 variants genotyped across the 450 donors. Of the 450 donors, 449 had RNA-sequencing data available in release v6p.

2.3 Imputation of autosomal genotypes

To increase resolution for discovering new eQTLs across GTEx tissues, we imputed variants from the 1000 Genomes Project into the quality-controlled Omni 5M+2.5M merged genotype data for 451 GTEx donors. The reference panel used was the 1000 Genomes Phase 1 integrated variant set release from March 2012 (release v3), updated on 24 August 2012 (additional problematic indels removed), and downloaded from the IMPUTE2 website: `https://mathgen.stats.ox.ac.uk/impute/data_download_1000G_phase1_integrated.html`. This version includes variants and indels and is limited to variants with more than one minor allele copy ("macGT1", or "minor allele count greater than 1") across all 1,092 donors.

In addition to the QC filters applied in **Supplementary Table 1**, we filtered out variants with incompatible alleles between the Omni 5M or 2.5M arrays and the 1000 Genomes reference data (4,475 variants), and variants with a frequency difference larger than 0.15 between GTEx and 1000 Genomes samples, computed using samples of European descent that constitute the majority of samples in GTEx (819 variants). Variants whose alleles did not align to the same DNA strand between GTEx samples and 1000 Genomes Project (human reference genome GRCh37/hg19) were removed.

Imputation was performed separately for variants on the autosomal and sex chromosomes using IMPUTE2 [4].

2.3.1 Imputation of autosomes

The imputation of autosomes was performed using the Ricopili pipeline (`https://sites.google.com/a/broadinstitute.org/ricopili/`). Pre-phasing was run on all samples together using SHAPEIT (`https://mathgen.stats.ox.ac.uk/genetics_software/shapeit/shapeit.html`) [5]. Imputation was performed on 3 Mb segments across chromosomes, which were subsequently merged. This yielded 14,390,153 variants across 451 samples before filtering on imputation quality (INFO score) or minor allele frequency (MAF). Imputation quality was high for common variants and lower for low frequency variants, as previously observed [6]. (**Supplementary Fig. 1** and **Table 3**).

The following QC filters were applied to generate a final analysis freeze of the genotyped and imputed array VCF for eQTL analysis: INFO < 0.4, minor allele frequency (MAF) $< 1\%$, Hardy-Weinberg Equilibrium (HWE) $p < 10^{-6}$ (only 9 variants were removed due to HWE filtering). We calculated the missingness rate for best-guessed genotypes and the HWE test using the SNPTEST software [7], using only donors of

European descent. Indels with a length above 51 base pairs were removed (~2,600 variants). About 13% of variants were hard call genotypes and 87% of variants were imputed. About 91% of the total number of variants (11,555,102) were SNPs and 8.9% indels. The REF and ALT alleles in the imputed VCF were verified for alignment to the human reference genome GRCh37/hg19, and the REF and ALT sequences (A,T,G,C) were added for both SNPs and indels.

The final genotyped and imputed array VCF (version 4.1) for autosomal variants contained genotype posterior probabilities for each of the three possible genotypes for 11,552,519 variants across 450 GTEx donors. The dosage of the alternative allele relative to the reference genome was used for eQTL analyses.

2.3.2 Evaluation of imputation accuracy

To assess the imputation accuracy on autosomal chromosomes, we used the 183 GTEx donors from the Pilot phase that were genotyped on Omni 5M, and compared the alternative allele dosages between imputed and genotyped calls, using the Omni 2.5S subset of variants (Omni 5M consists of 2.5M and 2.5S variant sets), for which both direct calls on the Omni 5M array and imputed calls from the merged set of 450 samples were available. Imputation accuracy was assessed for each of the 2.5S variants separately, through correlation analysis between the alternative allele dosage of the post-QC'd imputed calls and the directly genotyped calls across the 183 samples (**Supplementary Fig. 2a**). The imputation accuracy was high for common variants (median R^2 = 0.985-0.989), and lower, as expected, for low frequency variants (median R^2 = 0.804-0.976) **(Supplementary Table 4)**. These results are comparable with imputation accuracy reported in other projects, including the 1000 Genomes Project [6].

2.4 Quality control and imputation of chromosome X genotypes

Array merging and QC for chromosome X variants was performed as described for autosomal variants, with the following exceptions: (i) of the 636 variants that failed the heterozygous haploid test on the sex chromosomes in males (step 13 in **Supplementary Table 1**), heterozygous variants in the pseudoautosomal regions between chromosomes X and Y (PAR1 and PAR2) were restored. Heterozygous variants in the non-pseudoautosomal region of the X chromosome (nonPAR) were excluded; (ii) PAR1, nonPAR and PAR2 genotypes were imputed separately. For imputation purposes only, the PAR1 and PAR2 pseudoautosomal regions were treated as pseudo-diploid, and were distinguished from the nonPAR region that is diploid in females and haploid in males, by encoding the PAR regions with contig number 25 instead of 23. The nonPAR genotypes in males, even though hemizygous, were encoded as homozygous REF or homozygous ALT; (iii) strand alignment errors and missing variants between the GTEx samples and the 1000 Genome Project reference haplotypes were verified with SHAPEIT. The chromosome coordinates of the PAR1, PAR2 and nonPAR regions in chromosome X were taken from the UCSC browser (**Supplementary Table 5**).

Imputation of the X chromosome was performed as described for the autosomes, except for adding the --chrX flag for the imputation of the nonPAR region. The PAR1 and PAR2 regions were treated like autosomes. Imputation of the nonPAR region was performed on 5 Mb segments, with 300 kb overlap. The PAR1 and PAR2 regions were each imputed in a single, separate segment. All segments were then merged into a single VCF, and the three regions were labeled in the INFO field using the ChrX_REGION label.

2.5 Evaluation of imputation accuracy for chromosome X

The distribution of the IMPUTE2 imputation quality score, INFO, stratified by minor allele frequency (MAF) for the three chromosome X regions PAR1, PAR2, and nonPAR, is shown in **Supplementary Fig.**

3. Imputation performance for MAF $>$ 1% variants for the nonPAR region was comparable to that of the autosomes (**Supplementary Tables 3 and 6**). In the PAR regions performance was generally poor (**Supplementary Table 6**), likely due to the smaller number of variants and potentially higher recombination rates than in the nonPAR region in males [8].

Imputation of chromosome X yielded 1,000,339 genotyped and imputed variants. Post-imputation QC steps as described for autosomes (INFO $<$ 0.4, MAF $<$ 1%, HWE p $< 10^{-6}$, alignment to the reference genome) were applied to chromosome X variants, resulting in a filtered set of 406,892 variants (**Supplementary Table 7**). Of these, ˜6% (25,300 variants) were directly genotyped on the array and ˜94% were imputed (381,592 variants), and the majority are located in the nonPAR region (**Supplementary Table 7**). The HWE test was performed using the subset of female samples of European descent. The imputation accuracy of chromosome X variants after applying QC filters (**Supplementary Table 8**) was comparable to that of autosomal variants (**Supplementary Table 3**).

2.6 Population stratification analysis

We computed the principal components (PCs) of the genotyped and imputed variants from the 450 donors using EIGENSTRAT [9] as implemented in Ricopili (`https://sites.google.com/a/broadinstitute.org/ricopili/pca`). This was performed using a genome-wide set of linkage disequilibrium (LD)-pruned variants ($R^2 > 0.2$, plink --indep-pairwise 200 100 0.2) generated from best-guessed genotype calls after imputation (Posterior probability > 0.9). Variant filters were applied, including the exclusion of variants not present in all samples, strand ambiguous variants (AT, CG), variants in the MHC region, and variants with MAF $<$ 5%, HWE p $< 10^{-4}$, and variant missing rate $<$ 2%.

Plots of the first three PCs are shown in **Supplementary Fig. 4**. PCs 1 and 2 match the known ethnicity distribution of the donors, with a majority of samples of European ancestry and a smaller fraction (˜14%) of African ancestry. We did not identify any outlier samples based on ancestry. The distribution of ethnic backgrounds was similar between Omni 5M and 2.5M array samples, except for the few donors of Asian ancestry that were all genotyped on Omni 5M.

The first 20 PCs are available on dbGaP (phs000424.v6.p1). To correct for population stratification in eQTL analyses, the first 3 PCs were used as covariates, as they captured the largest proportions of genotype variance (**Supplementary Fig. 4a,b**). PC 9 is significantly correlated with *genotyping platform* (Omni 2.5M versus 5M array; $r = -0.18$, P ≤ 0.00053; **Supplementary Fig. 4c**), but we explicitly added this covariate to the eQTL model.

2.7 Functional annotation of variants in coding regions

We annotated the genotyped and imputed autosomal and chromosome X VCFs using the Variant Effect Predictor tool (VEP v77, GENCODE v19) from Ensembl (`http://useast.ensembl.org/info/docs/tools/vep/`) with the LOFTEE plugin (version 0.2.1, available at `https://github.com/konradjk/loftee`) to tag loss of function (LoF) variants. The functional annotations were added to variants in coding and intronic regions (sequence ontology terms below "INTRON_VARIANT" severity in the following ordering were filtered out: `http://useast.ensembl.org/info/genome/variation/predicted_data.html?redirect=no#consequences`). Additional LoF annotation was applied to variants that were annotated as "STOP_GAINED", "SPLICE_DONOR_VARIANT", "SPLICE_ACCEPTOR_VARIANT", and "FRAME_SHIFT" and the variants were flagged as low-confidence (LC) if any filters failed, otherwise as high-confidence (HC). The filters are described in the documentation at `https://github.com/konradjk/loftee`, and are annotated in the LoF, LoF_flag,

and LoF_filter fields inside the VEP annotation (CSQ) field. We used PLINK/SEQ to generate predictions of nonsense-mediated decay based on [10, 11].

2.8 Phasing of autosomal genotypes using haploid imputation

To facilitate the interpretation of functional mechanisms underlying regulatory regions, we phased the genotyped and imputed autosomal variants from the Omni arrays across the 451 donors. Since the standard IMPUTE2 variant imputation process only preserves phased information for directly genotyped variants, we performed haploid imputation to retain phasing information for imputed variants, using pre-phased haplotypes from the 1000 Genomes Project (Olivier Delaneau and Bryan Howie, personal communication). In this approach each haplotype of an individual is treated as a separate individual, and imputation is performed on the haplotypes generated during the pre-phasing step of the imputation process.

Haploid imputation was run on the same pre-phased haplotypes generated for the original diploid imputation of the Omni array genotype calls for the 451 donors using SHAPEIT (**see Section S2.3**). The output was diploid imputation probabilities. The main differences of the haploid imputation compared to standard diploid imputation in IMPUTE2 are: (i) The pre-phased genotype file (specified with the -g flag), generated with SHAPEIT on the hard genotype calls, was reformatted into "IMPUTE haploid genotype" format. The haploid genotype format is the same as a standard IMPUTE genotype format (five header columns, then three columns encoding the genotype per haploid haplotype which should never be heterozygous, e.g., a diploid genotype row: variant1 rs1 100 A C 0 1 will be encoded as: variant1 rs1 100 A C 1 0 0 0 0 1); (ii) the flag -haploid_g was added, specifying that the pre-phased genotype file is in haploid genotype format. Haploid imputation was performed on 3 Mb segments on each chromosome with an overlap of 300 kb, which were later merged. The haploid imputation across 451 GTEx donors resulted in 30,069,600 variant sites (variants and indels), before variant quality control filtering, 28,259,400 of which were imputed.

2.8.1 Evaluation of haploid imputation quality used for phasing

The imputation quality of autosomal variants using haploid imputation was comparable to standard diploid imputation (**Supplementary Tables 3 and 9**). To further assess the accuracy of haploid imputation, we computed the concordance between the alternative (ALT) allele dosages of 9,099,125 overlapping imputed variants from diploid imputation (**see Section S2.3**) and haploid imputation (missingness rate < 0.1 in both VCFs). Imputation accuracy was assessed by calculating the Pearson correlation for each variant between ALT dosages from the standard imputation and the haploid imputation across the 450 samples. **Supplementary Fig. 2b** shows the distribution of R^2 for all variants stratified by common (5-50%) and low frequency (1-5%) variants. Imputation concordance was high for common variants (mean R^2 = 0.997; median R^2 = 1 at INFO $>$ 0.4), and marginally lower for low frequency variants (mean $R^2 = 0.963$; median $R^2 = 1$ at INFO $>$ 0.4) (**Supplementary Table 10**).

2.8.2 Post-phasing variant quality control and filtering

To generate a final analysis freeze of the phased array VCF (version 4.1) of both genotyped and imputed variants, we filtered on the following cutoffs, identical to those used for the imputed array VCF described in Section S2.3: INFO $>$ 0.4, MAF $>$ 1%, HWE p $> 10^{-6}$ (402 variants violated HWE and were removed from the final VCF). The HWE test was only performed on samples of European descent, using the SNPTEST software [7]. Indels longer than 51 base pairs were removed. The REF and ALT alleles in the imputed

doi:10.1038/nature24277

VCF were aligned to the human reference genome GRCh37/hg19. The final phased array VCF contained 11,607,846 variants across 450 donors, and was used for allele-specific expression analyses.

3 Whole genome sequencing

3.1 Whole genome sequencing

Whole genomes were sequenced from 148 donors in GTEx. Of these, 68 samples were sequenced on Illumina's HiSeq 2000 and 80 on Illumina's HiSeq X. The mean coverage was 30x. Of the sequenced samples, two samples displayed large chromosomal abnormalities, including one with a chr17p mosaic trisomy and another with a mosaic loss of the Y chromosome (possibly around 75%; based on inspecting chromosome Y coverage of this sample).

3.2 Sequencing on Illumina HiSeq 2000

Libraries of whole genome DNA for 68 GTEx donors were constructed and sequenced on an Illumina HiSeq 2000 at the Broad Institute as 101-bp paired-end reads. Output from Illumina software was processed by the Picard data-processing pipeline to yield BAM files containing well-calibrated, aligned reads. All sample information tracking was performed by automated LIMS messaging.

3.2.1 Library construction

For the GTEx samples, library construction was performed as described in [12]. Initial genomic DNA input into shearing was reduced from 3 µg to 100 ng in 50 µL of solution. For adapter ligation, Illumina paired-end adapters were replaced with palindromic forked adapters with unique 8-base index sequences embedded within the adapter.

3.2.2 Size selection for whole genome shotgun libraries

Size selection was performed using gel electrophoresis, with a target insert size of either 340 bp or 370 bp +/- 10% (both sizes were selected). Multiple gel cuts were taken for GTEx sample libraries that required high sequencing coverage (30x). Size selection was performed using Sage's Pippin Prep. The 340 bp insert size was run on 1 sequencing lane and the 370 bp insert size was run on 2 lanes.

3.2.3 Library preparation for cluster amplification and sequencing

Following sample preparation, libraries were quantified using quantitative PCR (kit purchased from KAPA Biosystems) with probes specific to the ends of the adapters. This assay was automated using Agilent's Bravo liquid handling platform. Based on qPCR quantification, libraries were normalized to 2 nM and then denatured using 0.1 N NaOH using Perkin-Elmer's MultiProbe liquid handling platform. Denatured samples were diluted into strip tubes using the Perkin-Elmer MultiProbe.

3.2.4 Cluster amplification and sequencing

Cluster amplification of denatured templates was performed according to the manufacturer's protocol (Illumina) using HiSeq 2000 v2, or HiSeq v3 cluster chemistry and flow cells. For a subset of samples, after cluster amplification, SYBR Green dye was added to all flow cell lanes, and a portion of each lane was

visualized using a light microscope, in order to confirm target cluster density. Flow cells were sequenced on HiSeq 2000 using HiSeq 2000 v3 Sequencing-by-Synthesis Kits, and then analyzed using RTA v.1.12.4.2.

3.3 Sequencing on Illumina HiSeq X

Libraries of whole genome DNA for 80 GTEx donors were constructed and sequenced on the Illumina HiSeq X at the Broad Institute as 151-bp paired-end reads. Sample information tracking and output from Illumina software was handled in the same way as for the samples sequenced on the Illumina HiSeq 2000.

3.3.1 Library construction and size selection

Initial genomic DNA input into shearing was reduced from 3 μg to 100 ng in 50 μL of solution. In addition, for adapter ligation, Illumina paired end adapters were replaced with palindromic forked adapters with unique 8 base index sequences embedded within the adapter. Size selection was performed using Sage's Pippin Prep, with a target insert size of 370 bp +/- 10%.

3.3.2 Library preparation for cluster amplification and sequencing

Following sample preparation, libraries were quantified using quantitative PCR (kit purchased from KAPA Biosystems) with probes specific to the ends of the adapters. This assay was automated using Agilents Bravo liquid handling platform. Based on qPCR quantification, libraries were normalized to 1 nM. Samples were then combined with HiSeq X Cluster Amp Mix 1, 2, and 3 into single wells on a strip tube using the Hamilton Starlet Liquid Handling system.

3.3.3 Cluster amplification and sequencing

Cluster amplification of the templates was performed according to the manufacturers protocol (Illumina) using the Illumina cBot. Flowcells were sequenced on HiSeq X Sequencing-by-Synthesis Kits, and then analyzed using RTA2.

3.4 Variant calling and functional annotation

Whole genome sequencing data were processed through a pipeline based on Picard (`http://picard.sourceforge.net/`), using base quality score recalibration and local realignment at known indels. We mapped reads to human reference genome GRCh37/hg19 with the BWA-MEM aligner (`http://bio-bwa.sourceforge.net`). Variants and indels were jointly called across all 148 samples using GATK's HaplotypeCaller version 3.1 (`http://www.broadinstitute.org/gatk/gatkdocs/`) together with an additional 900 WGS samples from non-GTEx projects, to increase sensitivity and specificity of variant calls. The non-GTEx samples were subsequently excluded from the final VCF. Default filters were applied to SNP and indel calls using the GATK's Variant Quality Score Recalibration (VQSR) approach. An additional hard filter InbreedingCoeff <= -0.3 was applied to remove sites that VQSR failed to filter. Also, variants that fell in Low Complexity Regions (LCR) were flagged in the FILTER field. The coordinates of the LCR regions used as a filter mask can be found at: `https://github.com/lh3/varcmp/raw/master/scripts/LCR-hs37d5.bed.gz`.

Functional annotation was performed using the Variant Effect Predictor (VEP v80, GENCODE v19) tool from Ensembl with the LOFTEE plugin as described in **Section S2.7**.

4 Whole exome sequencing

Below is a brief description of the whole exome sequencing (WES) protocol and variant calling for 531 samples (524 donors), which is part of GTEx Release v6 (dbGaP accession phs000424.v6.p1), but was not used in analyses presented in this paper.

4.1 Whole exome sequencing

Whole exome sequencing was performed on 531 DNA samples from 524 GTEx donors, using Agilent Sure-Select Human All Exon v2.0, 44Mb baited target for the first 100 samples and Illuminas capture Exome (ICE) for the remainder 431 samples. Seven samples were run in duplicates on Agilent and ICE for quality control purposes. The WES samples include two samples with large chromosomal abnormalities (chr17p mosaic trisomy and mosaic loss of the Y chromosome), and a sample from a self-reported female that underwent transgender surgery at birth (contains Y chromosome).

4.2 Whole exome library construction, hybrid selection and sequencing

Exome sequencing was performed using the Broad's in-solution hybrid selection process [12]. The exome-sequencing pipeline included sample plating, library preparation (2-plexing of samples per hybridization for the Agilent target capture and 8-plexing for the ICE capture), hybrid capture, sequencing (76bp paired-end reads), sample identification QC check, and data storage. Library construction was performed as described in [12], using Agilent target capture, and with the following modifications for ICE: initial genomic DNA input into shearing was reduced from 3 μg to 100 ng in 50 μL of solution. For adapter ligation, Illumina paired end adapters were replaced with palindromic forked adapters with unique 8 base molecular barcode sequences embedded within the adapter to facilitate downstream pooling. The hybrid selection libraries covered $>$80% of targets at 20x with a mean target coverage of $>$80x. Cluster amplification of denatured templates was performed according to the manufacturer's protocol (Illumina) using HiSeq 2000 v2 or HiSeq v3 cluster chemistry and HiSeq 2000 or 2500 flow cells. Flow cells were sequenced on HiSeq 2000 or 2500 using HiSeq 2000 v2 or v3 Sequencing-by-Synthesis Kits, then analyzed using RTA v1.10.15, RTA v.1.12.4.2 or a later version. The WES data was de-multiplexed and each sample's sequence data were aggregated into a single Picard BAM file. Output from Illumina software was processed by the Picard data-processing pipeline to yield BAM files containing well-calibrated, aligned reads.

4.3 Variant calling and functional annotation

Exome sequencing data was processed through a pipeline based on Picard (`http://picard.sourceforge.net/`), using base quality score recalibration and local realignment at known indels. We used the BWA aligner (`http://bio-bwa.sourceforge.net`) for mapping reads to the human reference genome GRCh37/hg19. SNPs and indels were jointly called across all 531 samples using GATK's HaplotypeCaller version 3.1. The genomic intervals used for variant calling were Agilent's exome intervals for the first 180 pilot phase WES samples (the first 100 samples were sequenced with Agilent and the remaining 80 samples were sequenced with ICE), and ICE exome intervals for the subsequent 351 WES samples sequenced with ICE. Default filters were applied to SNP and indel calls using the GATK's VQSR approach. Functional annotation was performed using the Variant Effect Predictor (VEP v77, GENCODE v19) tool from Ensembl with the LOFTEE plugin as described in **Section S2.7**.

doi:10.1038/nature24277

5 RNA Expression

5.1 RNA library preparation and sequencing

RNA sequencing was performed at the Broad Institute using a large-scale, automated variant of the Illumina TruSeq™ RNA sample preparation protocol (`http://www.illumina.com/documents/products/datasheets/datasheet_truseq_sample_prep_kits.pdf`), which was based on poly-A selection of mRNA and was not strand-specific.

Briefly, total RNA was quantified using the Quant-iT™ RiboGreen®RNA Assay Kit and normalized to 5 ng per µL. An aliquot of 200 ng for each sample was transferred into library preparation, which was an automated variant of the Illumina Tru Seq™ RNA sample preparation protocol (Revision A, 2010). This method used oligo dT beads to select mRNA from the total RNA sample followed by heat fragmentation and cDNA synthesis from the RNA template. The resultant cDNA then went through library preparation (end repair, base 'A' addition, adapter ligation, and enrichment) using Broad Institute-designed indexed adapters substituted in for multiplexing. After enrichment, the libraries were quantified with qPCR using the KAPA Library Quantification Kit for Illumina Sequencing Platforms and then pooled equimolarly. The entire process was performed in 96-well plates and all pipetting was performed by either Agilent Bravo or Hamilton Starlet liquid handlers with electronic tracking throughout the process in real-time, including reagent lot numbers, specific automation used, time stamps for each process step, and automatic registration.

Pooled libraries were normalized to 2 nM and denatured using 0.1 N NaOH prior to sequencing. Flow cell cluster amplification and sequencing were performed according to the manufacturer's protocols using either the HiSeq 2000 or HiSeq 2500. Sequencing generated 76bp paired-end reads and an eight-base index barcode read, and was run with a coverage goal of 50M reads (the median achieved was ~82M total reads). Raw sequence data was processed using the Broad Institute's Picard pipeline, which includes demultiplexing and data aggregation steps.

5.2 RNA-seq alignment and quality control

RNA-seq data were aligned to the human reference genome GRCh37/hg19 with Tophat v1.4.1 [13]. Quality control of the samples was performed as described in [1]. Briefly, low-quality samples were identified and removed based on the following alignment metrics: $<$ 10 million mapped reads; read mapping rate < 0.2; intergenic mapping rate > 0.3; base mismatch rate > 0.008 (mismatched bases divided by total aligned bases); rRNA read rate > 0.3. Additionally, outlier samples were identified based on expression profile using a correlation-based statistic and sex incompatibility checks, following methods described in [14]. Among technical replicates (same aliquot sequenced multiple times for QC purposes), the sample with the highest number of reads was retained for inclusion in the analysis freeze set. Finally, samples from donors with cytogenetic anomalies (two donors with Klinefelter syndrome, and one transgender donor detected after sequencing) were excluded from analyses.

5.3 Analysis freeze of tissues and samples for eQTL analyses

After QC, the v6p release contained 8555 RNA-seq samples. Among these, samples were selected based on donor genotype availability and a threshold of at least 70 samples per tissue, resulting in a set of 7,051 samples from 44 tissues across 449 donors used for eQTL analyses. The tissues and samples are summarized in **Supplementary Fig. 5**, which also contains the abbreviations and color scheme used throughout the paper, and the distribution of RNA integrity number, ischemic time, and donor age within each tissue.

doi:10.1038/nature24277

5.4 Gene expression quantification

Gene-level expression quantification was performed using RNA-SeQC [15]. The quantification was based on the GENCODE Release 19 annotation (`http://www.gencodegenes.org/releases/19.html`), collapsed to a single transcript model for each gene, using a custom isoform collapsing procedure, comprising the following steps: 1) exons associated with transcripts annotated as "retained_intron" and "read_through" were excluded; 2) exon intervals overlapping within a gene were merged; 3) the intersections of exon intervals overlapping between genes were excluded; 4) the remaining exon intervals were mapped to their respective gene identifier and stored in GTF format. This annotation is available on the GTEx Portal (`gencode.v19.genes.v6p_model.patched_contigs.gtf.gz`).

Gene-level read counts and RPKM values were produced using the following read-level filters: 1) reads were uniquely mapped (corresponding to a mapping quality of 255 for TopHat BAMs); 2) reads were aligned in proper pairs; 3) the read alignment distance was ≤ 6; 4) reads were fully contained within exon boundaries. Reads overlapping introns were not counted. These filters were applied using the "-strictMode" flag in RNA-SeQC.

To evaluate the global relationships of tissue transcriptomes, we applied MDS clustering for all 44 tissues and for all brain tissues (**Supplementary Fig. 6**).

5.5 Normalization of expression data

Gene expression values for all samples from a given tissue were normalized for eQTL analyses using the following procedure: 1) genes were selected based on expression thresholds of > 0.1 RPKM in at least 10 donors and ≥ 6 reads in at least 10 donors; 2) the distribution of RPKMs in each sample was quantile normalized to the average empirical distribution observed across all samples; 3) expression values for each gene were subsequently transformed to the quantiles of the standard normal distribution.

5.6 Correction for technical confounders

To account for hidden batch effects and other potential confounders in the gene expression data, we used the Probabilistic Estimation of Expression Residuals (PEER) method to estimate a set of latent covariates for gene expression levels for each tissue type [16]. The number of PEER factors was selected to maximize cis-eGene discovery, and this optimization was performed for three sample size bins: tissues with fewer than 150 samples, tissues with ≥ 150 and < 250 samples, and tissues with ≥ 250 samples. Specifically, the eQTL discovery pipeline was run in increments of 5 PEER factors for 12 tissues spread across the sample size bins, using a reduced number of permutations (100 instead of the adaptive 1,000-10,000 used for all other analyses; see **Section S4 below**). Based on these results, and to avoid potential overfitting, 15, 30, and 35 PEER factors were selected, respectively for the three sample size bins (**Supplementary Fig. 7**). We did not have sufficient statistical power or sufficient numbers of trans-eQTLs to tune the number of PEER factors for trans-eQTL analysis without facing potential overfitting to spurious signal. Post-hoc analysis demonstrated no clear trend in number of trans-eQTL discoveries as we varied the number of PEER factors removed (**Supplementary Fig. 8**). Further, failure to remove confounding factors could result in false positive trans-eQTL associations [17]. Therefore, we opted to use the settings determined by the analysis of cis-eQTLs for the trans-eQTL analysis as well. This aggressive correction, explained 59-78% of total variance in gene expression levels (**Supplementary Fig. 9a**), however may lead to false negatives, reducing the signals for broad effect trans-eVariants with many target genes. Indeed, several loci with numerous associations were found in uncorrected data, but disappeared after controlling for PEER factors (**Supplementary Fig. 9b-d**).

However, the trans-eVariants detected before PEER correction were enriched for association with known technical confounders (**Supplementary Fig. 10**). With no ideal method available to optimize latent factor correction for trans-eQTLs, we chose to aggressively remove potential confounders using the same settings as the cis analysis.

PEER factors from each tissue were correlated with known technical and biological covariates recorded for each sample and donor (**Supplementary Fig. 11-12**). The covariates that were most consistently associated with PEER factors include factors related to parameters of donor death, ischemic time, RIN, and sequencing quality control metrics. Nucleic acid isolation and library construction batches and total sequencing depth were also moderately associated. Across tissues, the median percent variance explained (PVE) by RIN of the set of PEER factors used for correction was 0.05, with a maximum PVE of 0.13 in heart – left ventricle. The PVE by these covariates of the expression data after PEER correction was negligible – median $4 \times 10^{-}3$ for RIN. Similarly, after correction, the detected trans-eVariants show little association with known covariates. For example, the two tissues with the most trans-eQTLs, thyroid and testis, show no association between RIN and any trans-eVariant at FDR 50%. In addition, we have observed that minimal genetic signal is present in the PEER factors (**Supplementary Fig. 13**).

To control population effects on the discovery of eQTLs [18], we included the first three genotype PCs, which capture the major population structure among GTEx donors including Caucasian, African American, and Asian ancestry (**Supplementary Fig. 4, and Section S2.5**). Additionally, genotyping platform (Omni 5M or Omni 2.5M) and donor sex were included in the set of covariates in the association analysis.

6 Cis-eQTL mapping

Cis-eQTL mapping was performed using FastQTL [19]. The mapping window was defined as 1 Mb up- and downstream of the transcription start site, and the adaptive permutation mode was used with the setting `--permute 1000 10000`. For each tissue, variants in the VCF were selected based on the following thresholds: the minor allele was observed in at least 10 samples, and the minor allele frequency was $\geq$ 0.01. The beta distribution-extrapolated empirical P-values from FastQTL were used to calculate gene-level q-values [20], and a false discovery rate (FDR) threshold of $\leq$ 0.05 was applied to identify genes with at least one significant eQTL (“eGenes”).

To identify the list of all significant variant-gene pairs associated with eGenes, a genome-wide empirical P-value threshold, p_t, was defined as the empirical P-value of the gene closest to the 0.05 FDR threshold. p_t was then used to calculate a nominal P-value threshold for each gene based on the beta distribution model (from FastQTL) of the minimum P-value distribution $f(p_{\text{min}})$ obtained from the permutations for the gene. Specifically, the nominal threshold was calculated as $F^{-1}(p_t)$, where F^{-1} is the inverse cumulative distribution. For each gene, variants with a nominal P-value below the gene-level threshold were considered significant and included in the final list of variant-gene pairs.

All components of the single tissue cis-eQTL pipeline are available at `https://github.com/broadinstitute/gtex-pipeline`

6.1 Independent cis-eQTL mapping

Multiple independent signals for a given expression phenotype were identified by forward stepwise regression followed by a backwards selection step. The gene-level significance threshold was set to be the maximum beta-adjusted P-value (correcting for multiple-testing across the variants) over all eGenes in a given tissue. At each iteration, we performed a scan for cis-eQTLs using FastQTL, correcting for all previ-

ously discovered variants and all standard GTEx covariates. If the beta adjusted P-value for the lead variant was not significant at the gene-level threshold, the forward stage was complete and the procedure moved on to the backward stage. If this P-value was significant, the lead variant was added to the list of discovered cis-eQTLs as an independent signal and the forward step moves on to the next iteration. The backwards stage consisted of testing each variant separately, controlling for all other discovered variants. To do this, for each eVariant, we scanned for cis-eQTLs controlling for standard covariates and all other eVariants. If no variant was significant at the gene-level threshold the variant in question was dropped, otherwise the lead variant from this scan, which controls for all other signals found in the forward stage, was chosen as the variant that represents the signal best in the full model.

Using this approach, we were able to identify 1.3-fold more cis-eQTLs per tissue (**Supplementary Fig. 14**).

6.1.1 Multi-tissue independent cis-eQTL mapping

For analyses in the functional characterization of cis-eQTLs, we ran a modified version of forward stepwise regression to select an ordered list of independent variants associated with a given gene across all tissues types. In each step k, we identify variants associated with expression of each gene across tissues, and refer to these as the 'tier k variants'. In each tier k, for each tissue, Matrix-eQTL was run independently for each gene that had a variant added to the model at every previous step $1..k-1$ (all genes are assessed in tier 1). In each tier, any significant variants identified in tiers $1..k-1$ are included as covariates. Significant tier k variants were assessed as follows. For each tissue, we obtained gene-level P-values for tier k via eigenMT [21]. Genome-wide significance of multiple independent variants per gene (in each tissue independently) was assessed via Benjamini-Hochberg (FDR < 0.05) for all gene-level P-values tested in tier k combined with all those tested in previous tiers [22]. To identify the cross-tissue tier k variant for a given gene, we selected the variant (out of all variants genome-wide significant for the gene in at least one tissue) with the smallest geometric mean P-value (across tissues). If no variant was genome-wide significant, no cross-tissue tier k variant was selected for that gene, and that gene will be estimated to have $k-1$ total independent cross-tissue variants. If a particular tissue's tier j genome-wide significant variant for a particular gene differed from the cross-tissue tier j variant for the same gene, the P-value of that tissue's tier j genome-wide significant variant was used in the Benjamini-Hochberg procedure. If a particular gene's cross-tissue variant for tier k does not meet genome-wide significance in all tissues in the tier $(k+1)$ step due to increased multiple testing, that gene will be conservatively considered to have $(k-1)$ independent cross-tissue variants.

Normalized H-C contact data were collected from published data [23]. We averaged Hi-C counts between the 5kb window around each variant and the 5kb window around the gene TSS, of primary and secondary eVariants compared to background variant-TSS pairs. Background variant-TSS pairs were matched for MAF and distance to TSS.

7 Trans-eQTL Quality Control

Quality control for trans-eQTLs was performed as follows. Mappability of every k-mer of the reference human genome (hg19) computed by the ENCODE project35 has been downloaded from the UCSC genome browser (accession: wgEncodeEH000318, wgEncodeEH00032)76. We have computed exon- and untranslated region (UTR)-mappability of a gene as the average mappability of all k-mers in exonic regions and UTRs, respectively. We have chosen $k = 75$ for exonic regions, as it was the closest to GTEx read length among all available values of k. However, as UTRs are generally small regions, and 36 is the smallest among

all possible values of k, we have chosen $k = 36$ for UTRs. Finally, mappability of a gene is computed as the weighted average of its exon-mappability and UTR-mappability, weights being proportional to the total length of exonic regions and UTRs, respectively. We excluded from association testing any gene with mappability < 0.8.

The set of genetic variants tested have also been reduced by first filtering out all variants with MAF < 0.05 in donors sampled for the tissue being tested (reducing the variant set to 6,226,121), and then filtering out all variants that are annotated by RepeatMasker to belong to a repeat region `http://www.repeatmasker.org`, release library version 20140131 for hg19. This filtering reduced the number of variants tested by roughly 53.6%, from 6,226,121 variants to 2,889,379. Genotyping in these regions remains subject to potential errors from probe mislocalization due to sequence similarity across the genome. Furthermore, nearby linked variants can still capture signal from these regions for strong associations.

Next, we aligned every 75-mer in exonic regions and 36-mers in UTRs of every gene with mappability below 1.0 to the reference human genome (hg19) using Bowtie (v 1.1.2) [24]. If any of the alignments started within an exon or a UTR of another gene, then that pair of genes are cross-mappable. We excluded from consideration any variant-gene pair where the variant is within 100 Kb of a gene that cross-maps with the potential trans-eQTL target gene.

While controlling for three genotype PCs should capture most broad effects of ancestry, we additionally checked for residual evidence of strong correlation with a larger set of 20 genotype PCs (**Supplementary Table 12**). We observed a modest increase in correlation among trans-eVariants (**Supplementary Fig. 15**). While we opted not to apply further filtering, we have flagged any trans-eVariant with maximum correlation greater than 99% of the levels observed among random variants for use in downstream analyses that may depend on ancestry.

8 Trans-eQTL False Discovery Rate

We assessed FDR in the trans-eQTL hits in two ways. First, we computed, per tissue, the Benjamini-Hochberg FDR using the Matrix eQTL P-values from an all genes by all variants association test (*genome-wide FDR*). Second, we computed the adjusted gene-level FDR by taking, per tissue, the most extreme P-value per gene, multiplying that P-value by 1,000,000 (to account for the number of effective independent tests in a trans-association; borrowed from the effective number of tests assumed in the canonical WTCCC Bonferroni correction of 5×10^{-8} with a threshold of 0.05), and using Benjamini-Hochberg on those adjusted extreme P-values across genes to compute the FDR (*gene-level FDR*). We set a universal FDR threshold of 10% FDR.

Using the genome-wide FDR, we found 94 genes—93 unique genes, with one found in both testis and thyroid—with one or more trans-eQTLs. Using the gene-level FDR, we found a total of 46 trans-eGenes, where 42 overlap with the 93 genes in the genome-wide FDR set, and four additional genes not found in the genome-wide FDR. Because of trans-eVariants impacting multiple eGenes, we found 43 trans-eVariants at gene-level FDR of 0.1. Of the 51 eGenes that were discovered at the genome-wide FDR but not at the gene-level FDR, 17 of those are gene-level FDR ≤ 0.2 and all 51 are gene-level FDR ≤ 0.4; the results from the two approaches are not dramatically different. We report all of these results in Extended Data Table 1. For all downstream analyses, we used the genome-wide FDR set.

9 Intra-chromosomal long-range eQTL mapping

Phased allelic expression data were collected for all LD pruned eQTLs (FDR ≤ 0.1) and only those eQTLs with data in at least ten eVariant homozygotes and heterozygotes were used. To remove cases where strong allelic imbalance was seen in eQTL homozygotes, the top 5% of eQTLs sorted by homozygote allelic imbalance were filtered. To minimize the number of phasing errors that occur at long, chromosome wide distances, we developed a model that predicts the probability of phasing error as a function of the minor allele frequency of both the eVariant and a coding variant where ASE is assessed, as well as the distance between them. We used this model to filter cases where the predicted probability of correct phasing was < 99%. A beta-binomial mixture model was then used to determine if the allelic data supported the presence of a cis-eQTL.

To identify long-range cis-eQTLs, from eQTLs with TSS distance > 5 Mb the top eQTL per gene was selected, and multiple testing correction was performed using the Benjamini-Hochberg FDR method on a per-tissue basis. We next quantified the proportion of eQTLs with significant (nominal P ≤ 0.01) ASE supported evidence of cis-regulation as a function of distance to eGene TSS. Although we attempted to reduce phasing error, we were unable to accurately estimate the remaining error, so we compared the observed proportion of cis-eQTLs to what would be expected under the worst case scenario of phasing error. Performance under the worst case scenario was determined by introducing phasing error between eVariants and ASE data at a rate of 50% to LD pruned eQTLs (FDR ≤ 0.1) within 100 Kb of the TSS, which were assumed to act in cis, and then determining the number of significant (nominal P ≤ 0.01) ASE supported cis-eQTLs that could be identified as a function of eQTL effect size.

10 Trans-eQTL restricted discovery association mapping

To improve statistical power to identify trans-eQTLs, we filtered our discovery genotypes in two ways. First, we restricted our association testing to a restricted subset of variants to control for linkage 109 disequilibrium (LD). To do this, we pruned the set of genotyped and imputed variants to have local genotype $R^2 < 0.5$ by random selection, agnostic to gene expression levels or functional annotation of variants. This LD-pruning led to a set of variants that included approximately 10% of the original variant set. While this may result in false negatives by eliminating some of the strongest associations, it also has the potential to reduce false positives that are not supported by associations with well-correlated variants in the same LD block. Performing association mapping in this reduced set, we found 54 variants affecting 47 genes across 17 tissues (FDR ≤ 0.1). Next, we performed a trans-eQTL association test restricting the variants to the set of cis-eVariants (top variant per cis-eGene) and testing for trans association with all genes on any other chromosome than the variants own. In the cis-eQTL restricted analysis, we found 41 eVariants affecting 33 genes across 17 tissues (FDR ≤ 0.1). All of these results are reported in **Supplementary Table 13**.

11 Allele-specific expression

Allele-specific expression was performed as described in the Online Methods. We investigated several distributional and quality-control properties of GTEx ASE including: the number of genes with tested ASE variants per donor, the number of genes with tested ASE variants per tissue, the number of tissues with tested ASE variants per gene, the number of genes with significant (binomial test versus 0.50, 5% FDR) ASE variants per donor, the number of genes with significant (binomial test versus 0.50, 5% FDR) ASE variants per tissue, the number of tissues with significant (binomial test versus 0.50, 5% FDR) ASE variants

per gene and the distribution of reference ratios (reference allele count / (reference + alternative allele count)) (**Supplementary Fig. 16**).

12 Multi-tissue analysis

12.1 Hierarchical FDR control for cis-eQTLs

We applied a separate hierarchical multiple testing correction method to identify multi-tissue eGenes [25, 26]. First, we constructed a p-value for each eGene across tissues using the Simes combination rule [27] on the tissue-specific beta-approximation P-values provided by FastQTL. Storey's q-value method [28] was then used to identify eGenes that are active in any tissue. To identify the specific tissues in which these eGenes are regulated, we applied the Benjamini and Bogomolov procedure [29], a special case of the more general procedure proposed in [26] at the 0.05 level. This approach not only allowed us to control the FDR for the discovery of eGenes across tissues and the expected average proportion of false tissue discoveries across these eGenes, but also to gain power to detect eGenes in tissues with smaller sample sizes when there is evidence from other tissues supporting their regulation.

12.2 Hierarchical FDR control for trans-eQTLs

We applied a hierarchical FDR control approach to identify significant trans-eVariants across all variants, genes, and tissues as a second assessment of tissue-specificity of trans-eQTLs [25,26]. As input, we considered 306,061 variants from the LD-pruned set that had a nominal trans association $P \leq 1.0 \times 10^{-7}$ with at least one gene. To identify eVariants, the genes to which they are associated, and the tissues in which these associations are present, we performed a hierarchical testing procedure [25, 26]. Specifically, we used a three-level version of the multi-level hypothesis testing method [26] with variants in Level 1, genes in Level 2, and tissues in Level 3 of our hypothesis tree. P-values were defined starting from the Level 3 hypotheses regarding the association of variant i to the expression of gene j in tissue k, where we used the association P-value p_{ijk} calculated by Matrix eQTL. P-values $p_{ij\bullet}$ corresponding to Level 2 the variant $\times$ gene null hypotheses of no association between variant i and gene j in any tissue were then calculated using Simes [27], and p-values $p_{i\bullet\bullet}$ corresponding to the Level 1 null hypotheses of no association between variant i and any gene in any tissue were also calculated using Simes. We then applied the treeBH procedure [26] on $p_{i\bullet\bullet}$ to identify eVariants, the genes these eVariants control, and the tissues in which this regulation is present. This three-step procedure controls the FDR of eVariants, the average expected proportion of false variant-gene associations across eVariants [25], and the expected weighted average of false tissue discoveries for the selected variant-gene pairs (weighted by the size of the eVariant and eGene sets) to the target FDR ≤ 0.1.

12.3 Multi-tissue eQTL sharing

We ran Meta-Tissue on the subsampled cis-eQTL data for each tissue ($n = 70$) and considered an cis-eQTL to be active in a tissue if it had a Meta-Tissue m-value ≥ 0.9 in that tissue. For genes expression in both tissues, we measured the sharing of eQTLs affecting those genes with the Jaccard index:

$$J(t_1, t_2) = \frac{|t_1 \cap t_2|}{|t_1| + |t_2| - |t_1 \cap t_2|}$$

where t_1 and t_2 are the set of eQTLs active in the first and second tissues respectively. We compared the Jaccard index for each pairwise combination with the correlation of expression between those tissues. The

Pearson's product-moment correlation between each pair of tissues was estimated using the median $\log_2$-transformed RPKM values for each gene in each tissue. In general we saw a trend whereby tissues with highly correlated expression shared a greater proportion of multi-tissue eQTLs (**Supplementary Fig. 17**). Pairwise comparisons between brain tissues nicely demonstrated this phenomenon.

13 Fine-mapping analyses

13.1 CAVIAR

CAVIAR (CAusal Variants Identification in Associated Regions) [30] uses LD structure to model the observed marginal test statistics for each eGene as following a multivariate normal distribution (MVN). Applying this model, CAVIAR can define a credible set containing all causal variants with probability ρ. To define these credible sets in each tissue, we used a threshold of ρ = 90%. The mean credible set size was calculated for the top 1000 cis-eQTLs per tissue (**Supplementary Fig. 18a**).

13.2 CaVEMaN

We used CaVEMaN (Causal Variant Evidence Mapping with Non-parametric resampling) to estimate the probability that an eVariant was a causal variant (Brown et al., submitted). We used a non-GTEx reference cis-eQTL data set from subcutaneous adipose tissue, lymphoblastoid cell lines, skin, and whole blood, to simulate causal variants with characteristics matching genuine cis-eQTLs [31] (effect size, residual variance, minor allele frequency, and distance to the TSS). For each simulation, we calculated the proportion of times the simulated causal variant was among the ith most significant eVariants and denoted this proportion as p_i. For each lead eVariant in GTEx, we generated a "single-signal" expression phenotype by controlling for all covariates fitted in the cis-eQTL mapping and all other eVariants for the gene except the eVariant whose signal we wished to preserve. These data were sampled with replacement 10,000 times and cis-eQTL mapping was performed on each resample. The proportion of times a given eVariant was ranked i was calculated, denoted F_i. The CaVEMaN score is then defined as $\sum_{i=1}^{10} p_i \cdot F_i$. To calibrate CaVEMaN scores, across all genes and tissues simulated (removing blood as an outlier) we divided the CaVEMaN scores of the peak variants into twenty quantiles. Within each quantile, we calculated the proportion of times the lead variant was the causal variant and then drew a monotonically increasing smooth spline from the origin, through the 20 quantiles, to the point (1, 1) using the gsl interpolate functions with the steffen method (gsl-2.1, `https://www.gnu.org/software/gsl/`). This function provides our mapping of CaVEMaN score of the lead variant onto the probability it is the causal variant, calibrated using the simulations (**Supplementary Fig. 18b**).

14 Effect-size calculations

14.1 cis-eQTL effect size

cis-eQTL effect size was defined as the ratio between the expression of the haplotype carrying the alternative eVariant allele to the one carrying the reference allele in $\log_2$ scale and was calculated using the method in companion work [32]. In short, the model assumes an additive model of expression in which the total expression of a gene in a given genotype group is the sum of the expression of the two haplotypes: e(genotype) = $2e_r$, $e_r + e_a$, $2e_a$, for reference homozygotes, heterozygotes, and alternate homozygotes,

respectively, where e_r is expression of the haplotype carrying the reference allele and e_a, expression of the haplotype carrying the alternative allele is: $e_a = ke_r$ where $0 < k < \infty$.

cis-eQTL effect size is represented in $\log_2$ scale as $s = \log_2 k$, and is capped at 100-fold to avoid outliers ($|s| < \log_2 100$). Expression counts were retrieved for all top eGenes in all tissues and PEER corrected. Data was log-transformed with one pseudo-count to stabilize the variance. The model was fit using non-linear least squares to derive maximum likelihood estimates of the model parameters k and e_r. A similar maximum likelihood approach with additive effects and multiplicative errors (prior to log transformation) [33] was compared in several tissues to the effect size estimates reported here, exhibiting rank correlation ~ 0.98. Confidence intervals for the effect sizes were derived using bias corrected and accelerated (BCa) bootstrap with 100 samples.

For all analyses in a given tissue only the top eVariant per eGene was used. Only those eQTLs whose 95% confidence interval of the effect size estimate did not overlap zero were used for downstream analysis. To control for differences in power due to eVariant allele frequency, the effect of MAF on eQTL effect size was estimated using LOWESS regression (Matlab function `malowess: span=0.2, robust=true`), and was subtracted from the effect sizes on a per tissue basis.

14.2 ASE effect size

For each sample, haplotypic expression at all eGenes was calculated by summing counts from all phased, heterozygous variants. For a given cis-eQTL variant, assume x_i is the number of RNA-seq reads aligned to one haplotype, and y_i is the total number of reads aligned to either haplotype in the ith individual. Regulatory effect size of the cis-eQTL was calculated as median log-ratio: $s(x, y) = median[\log_2(x_i) \log_2(y_i - x_i)]$. Effect sizes were calculated for cis-eQTLs for which 10 or more donors with $y_i \geq 10$, and the effect sizes were constrained to be less than 100 fold ($|s(x, y)| < \log_2 100$). Confidence intervals for the effect sizes were derived using BCa bootstrap with 100 samples .

15 Mendelian randomization (MR) for shared cis-eQTLs and trans-eQTLs

For every trans-eQTL (genome-wide FDR ≤ 0.1) and for every cis-eQTL (P $\leq 10^{-5}$), we identified the variants in the intersection of cis-eQTL RSIDs and trans-eQTL RSIDs. Doing this, we found 296 joint cis-eQTLs and trans-eQTLs. In order to measure the regulatory causal effect of cis-eGenes to trans-eGenes for our set of 296 cis-trans-eQTLs, we used an instrumental variable (IV) analysis, using the Mendelian randomization method implemented by McDowell et al. [34]. After correcting the expression values for the same covariates as the main analysis, we calculated the Wald test statistic, t_{MR}, as follows:

$$t_{MR} = \frac{\beta_{MR}^2}{var(\beta_{MR})} \quad (1)$$

$$\beta_{MR} = \frac{\hat{\beta}_{y,z}}{\hat{\beta}_{x,z}} \quad (2)$$

$$var(\beta_{MR}) = \sigma^2 (x^T P_z x)^{-1} \quad (3)$$

$$\sigma^2 = \frac{(y - x\beta_{MR})^T (y - x\beta_{MR})}{n - \nu} \quad (4)$$

$$P_z = z(z^T z)^{-1} z^T, \quad (5)$$

$$(6)$$

where z is our instrumental variable (eVariant), x is our cis-eGene expression levels, y is our trans-eGene expression levels, n is the number of samples, and $\hat{\beta}_{y,z}$ and $\hat{\beta}_{x,z}$ each represent the trans-eQTL and cis-eQTL effect sizes, respectively. The degrees of freedom, ν was set to 3. We then computed the p-value corresponding to the Wald test statistic.

In addition to our 296 cis-trans-eQTLs, we also performed this MR test for the thyroid and skeletal muscle examples in the main text - for thyroid, rs1867277 was the IV, *TRMO* and *FOXE1* were cis-eGenes, and *TMEM253* and *ARFGEF3* were trans-eGenes. For skeletal muscle, rs2706381 was the IV, *IRF1* was the cis-eGene, and *PSME1* and *ARTD10* were the trans-eGenes. The null distribution was generated by taking 100 permutations of the trans-eGene value and calculating the Wald test statistic (**Fig. 4a**).

16 Replication of cis- and trans-eQTLs

To assess replication of cis- and trans-eQTLs, we examined P-values for matched variant-gene pairs in the TwinsUK data [35]. For the cis-eQTLs, the two GTEx adipose tissues were compared to the TwinsUK subcutaneous adipose, the two GTEx skin tissues against the TwinsUK sun-protected skin tissue, and GTEx LCL and whole blood tissues against these same tissues in the TwinsUK dataset. For the trans-eQTLs, all GTEx hits were compared to the matched gene-variant pair in all four TwinsUK tissues.TwinsUK RNA-seq data were mapped to the GRCh37 reference genome [36] using GEM version 1.7.1 [37], and genes were quantified to RPKM values using the GENCODE 19 annotation [38].

To assess cis-eQTL replication, genes with $> 50\%$ zero RPKM values across donors were removed from further analysis. RPKM values were scaled and centered, and then 50 principal components were regressed out (25 for blood due to the smaller sample size). Data were then mapped to a normal distribution. The `lmer` R package [39] was used to calculate a P-value for association between gene expression and genetic variant, controlling for family structure using random effects.

To assess trans-eQTL replication, RPKM values were transformed to the quantiles of the normal distribution. The `lmer` R package [39] was used to calculate a P-value for association between gene expression and genetic variant, controlling for family structure, primer index and date of sequencing using random effects, and GC content and insert size of the sequencing sample, age and BMI of the donor as fixed effects.

Finally, we evaluated replication of two trans-eQTL associations from LCLs identified in the complex trait-associated variant-restricted analysis. We tested these trans-eQTLs in the GEUVADIS data ($N = 462$) [40], but did not find signal of association for either eQTL (P ≤ 0.93, rs3125734; P ≤ 0.64, rs10520789).

17 Thyroid-specific TF *FOXE1* candidate master regulator

In the trans-eQTLs discovered in thyroid tissue samples, post-hoc analysis demonstrated that PEER correction dampened broad regulatory effects of the 9q22 locus. To explore the drivers of this broad distal regulation, we examined two nearby genes, *TRMO* and *FOXE1*, a different regulatory effect was observed, where association was dampened for *FOXE1* (P $\leq 2.0 \times 10^{-2}$ and P ≤ 0.54, before and after PEER correction, rs1867277) and induced for *TRMO* (P $\leq 6.7 \times 10^{-7}$ and P $\leq 8.3 \times 10^{-13}$, before and after PEER correction, rs1867277). Furthermore, using *FOXE1* as the cis-target gene, co-localization posterior probability [41] of the cis-eQTL with the trans-eQTL changed from 0.044 and 0.28 before PEER correction to 0.055 and 0.055 after PEER correction for *TMEM253* and *ARFGEF3*, respectively. On the other hand, co-localization posterior probability was > 0.99 for both *TMEM253* and *ARFGEF3* when *TRMO* was considered the cis-eGene after PEER correction, while it was > 0.97 and > 0.99, respectively, before PEER correction.

18 TCGA thyroid RNA-seq analysis

To replicate trans-eVariants in thyroid, we used Thyroid Carcinoma (THCA) RNA-seq and genotype array data from The Cancer Genome Atlas (TCGA). Filtering out tumor normal and metastatic samples, we restricted our analysis to 498 primary tumor samples. Next, after log transforming RNA-seq RSEM measurements, we ensured that expression of each gene follows a Gaussian distribution by projecting each gene expression levels to the quantiles of a standard normal. To account for noise and confounding factors in RNA-seq measurements, we corrected the data by 35 PEER factors. Using a linear model while adjusting for 35 PEER factors with MatrixeQTL, we tested the effect of each variant on chr 9 position 100600000 - 100670000 on expression levels of all trans genes. We used the Benjamini-Hochberg method to correct for multiple hypotheses testing (assessed only among 24 variants tested). Genes with FDR ≤ 0.1 were called as trans-eGenes.

19 Author Contributions

19.1 Lead Analysts

- F.A. developed the cis-eQTL pipeline. Generated the cis-eQTLs. Generated the expression data used throughout the manuscript. Contributed to study design. Performed PEER optimization analysis. Performed GWA with PEER factors in Supplement. Performed covariate analyses. Performed multi-tissue cis-eQTL analyses based on METASOFT. Wrote Sections 1, 2 (with A.V.S.), 5, 6 of the supplement; generated multiple supplementary figures. Contributed data used in Fig. 1 and corresponding EDFs. Contributed text to the manuscript.

- A.Br. developed and ran the multiple eQTL mapping procedure and produced summaries presented in EDFs. Developed and ran the CaVEMaN fine-mapping approach and produced summaries presented in manuscript and EDFs. Performed replication of cis and trans-hits in TwinsUK study. Produced methods description for these analyses and text for results of multiple eQTL mapping and CaVEMaN.

- S.E.C. developed the pipeline for generating ASE data and assisted the LDACC with implementing it. Performed quality control of ASE data. Performed analysis of ASE data described in main text section on ASE. With P.M., generated eQTL and ASE effect size estimates. Performed analysis of eQTL effect sizes described in paragraph of main text on functional characterization of cis-eQTLs, and shown in Fig. 3d, 3f, and EDFs. With P.M. developed model for estimating cis-effects of eQTLs using ASE data. Applied model to intrachromosomal trans-eQTLs, described in main text in section on eQTLs across human tissues and EDFs. Wrote manuscript text, figure legends, supplementary methods for sections and analyses mentioned above.

- J.R.D generated Fig. 1 and Fig. 5b. Generated multiple EDFs. Performed subsampling analyses for cis-eQTL calling. Generated the map of GTEx tissues to Roadmap Epigenomic tissues. Contributed analysis defining eGenes and non-eGenes across all tissues. Helped to organize the EDFs into a single document. Contributed writing for the main text section on eQTLs across human tissues. Reviewed and edited manuscript, supplement and EDFs.

- B.J. designed and performed the main trans-eQTL analysis with B.E.E. and A.B. Wrote the trans-eQTL portions of main manuscript and supplement.

- Y.H. contributed to the development of the pipeline for trans-eQTL analysis, including normalization and filtering for ambiguous mapped genes. Contributed to cis-eQTL analysis with overlapping & non-overlapping samples from different tissues. Investigated the functional roles for eGene and eVariants in downstream trans-eQTL analysis.

- P.M. developed cis-effect size model and calculated the eQTL and ASE effect sizes presented in Figure 3d,e,f, several EDFs and the GTEx portal. Helped S.E.C. with downstream analysis. Developed the Bayesian model used for finding cis-signal in long range intrachromosomal eQTLs in trans-eQTL analysis reported in the main text and EDFs and contributed to the rest of the analysis which was done by S.E.C. Performed analysis to address the effect of PEER factor inclusions on the eQTL/ASE effect size concordance as requested in the initial review. Contributed to designing the ASE data generation and the eQTL data preprocessing pipelines with S.E.C and F.A. Contributed to text and other material concerning the above-mentioned analyses.

- P.P. Conducted trans-eQTL thyroid replication and analysis with TCGA thyroid cancer data. Conducted analysis to generate Fig. 6. Performed gene set enrichment of muscle trans-eGenes. Analyzed association of PEER factors and known confounders. Analyzed association of trans-eVariants lost after PEER correction with known sample covariates. Investigated role of eVariants and eGenes in thyroid and muscle and wrote related sections with A.B. in the main manuscript. Generated figure 4C from piRNA results. Helped with putting together Fig. 4 and miscellaneous EDFs.

- Y.P. developed and applied pipelines for Meta-Tissue analysis to assess tissue-specificity and sharing of all cis-eQTLs, which resulted in panel(s) of Figure 2, Figure 3 and corresponding EDFs within the section ”Tissue-sharing and specificity of eQTLs”. Developed and applied pipelines for colocalization analysis to assess relationship between eQTLs and 23 GWAS complex traits, which resulted in panel(s) of Figure 5 and section ”The relationship of eQTLs to complex disease associations”. Contributed to reviews, discussions and edits of various analyses and manuscript writing overall.

- A.V.S. merged the OMNI 5M and 2.5M array genotypes. Assessed imputation vs hard calls. Developed and applied variant and sample quality control pipeline to the genotyping data before and after imputation (imputation performed by S.R.). Conducted analyses presented in supplement. Conducted quality control of WES and WGS data. Wrote the Supplementary Information 2-4: Genotyping and imputation, Whole genome sequencing, and Whole exome sequencing. Supervised the generation of METASOFT results and their comparison to FastQTL results. Helped design the GWAS section. Contributed text to the manuscript.

- B.J.S performed multi-tissue step-wise cis-eqtl mapping and analysis. Performed cross-tissue (Meta-Tissue and replication rate modeling) analysis for trans-eqtls. Ran cis-regulatory enrichment analysis for trans-eqtls.

- Z.Z. collected and analyzed data necessary to generate EDFs describing the tissue-specificity of cis-eQTLs. Analyzed cis-eQTL sharing analysis run in Meta-Tissue, used to generate Figure 2B and corresponding EDFs. Analyzed fine-mapping data (CAVIAR and CAVEMAN) to produce EDFs and manuscript text. Compared gene expression and eQTL sharing. Helped write the manuscript and supplement.

19.2 LDACC Authors

- B.B.C, E.T.G., A.G.,G.G., K.H., K.H.H., M.L., J.L.N., D.Y.N, M.S.N., T.T., D.G.M. and G.G. contributed to study design, data generation and quality control.
- Xiao L. evaluated imputation accuracy of autosome and chrX. Performed phasing of autosomal genotypes using haploid imputation, performed METASOFT analyses, performed initial quality control of WGS and WES data, Wrote or contributed to writing Supplementary Information for sections and analyses described above.
- T.J.S. performed quality control of the RNA-seq data; developed the ASE pipeline with input from S.E.C.; coordinated data sharing and release.

19.3 NIH Program Management Authors

- A.A., P.G., S.K., R.L., N.C.L., H.M.M., A.R., J.P.S. and S.V. contributed to study design and program management.

19.4 Biospecimen Collection Authors

- L.E.B., R.H., M.H., C.J., M.J., G.K., W.F.L., J.T.L., A.M., B.M., K.M., B.R., M.S., S.S., J.A.T., G.W., M.W., J.W., J.B., B.A.F., B.M.G., E.K., R.K., M.M., M.T.M., S.D.J., R.G.M., D.C.R., D.V., D.A.D and D.C.M. contributed to study design and sample collection.

19.5 Pathology Authors

- L.S., M.E.B and P.A.B contributed to study design and histology examination of samples.

19.6 eQTL Manuscript Working Group Authors

- N.S.A. generated significance and effect size analyses in EDFs and Fig. 5c. Helped compile and format paper and EDFs.
- B.B. performed analysis for the characterization of the properties of eGenes and non-eGenes. Generated corresponding EDFs. Helped with compiling the EDF section and paper editing.
- L.F. performed Gene Ontology Enrichment analysis on non-eGenes. Generated corresponding EDFs. Helped with paper editing.
- E.R.G., A.G., A.Z.H., E,Y,K, I.C.M, H.O., H.Z., Y-H. Z., O.D., C.D.Bu., N.J.C., R.G., M.K., M.I.M., F.A.W, K.G.A and E.T.D. contributed to study design and analysis.
- G.Gl. performed colocalization analysis on trans-eQTL and cis-eQTL for the Thyroid and Muscle Tissue.
- M.J.G. performed π_1 replication analysis across tissues, and effect size comparisons for tissue-shared and tissue-specific eQTLs. Helped with compiling paper and EDFs.
- B.H. and J.H.S. contributed to the development of METASOFT, Meta-Tissue and PM-PLOTS and application of these methods to the GTEx data.

- F.H. contributed to the development of CAVIAR and eCAVIAR and the application of CAVIAR and eCAVIAR to the GTEx data.
- X.L. contributed to shared/tissue-specific eQTL analysis and eQTL to complex disease analysis.
- B.L. generated Fig. 5b: number of variants passing nominal p-value and Bonferroni corrected p-value thresholds. Generated METASOFT and Meta-Tissue data for subsampled data: comparing tissue-by-tissue and joint eQTL calling. Generated multi-dimensional scaling plot for expression across tissues and across brain tissues.
- D. G-M. led analyses of genetic effects on transcript abundance in the GTEx AWG.
- J.P. contributed to the design, implementation, and validation of cis-eQTL effect size estimation model. Ran effect-size estimations for full-genome eQTLs.
- C.B.P. developed and applied hierarchical multiple testing procedure (TreeQTL) to cis and trans data; wrote text included in main manuscript and supplement describing the procedure and results; provided data for and/or helped construct EDFs.
- G.Q. contributed to data generation and analysis (LD maps).
- S.R. quality-controlled and imputed genotype data.
- A.S. contributed to data correction pipeline design and implementation; generated average mappability of genes; generated list of cross-mappable gene pairs; helped generate trans-eQTL using matrix-eQTL; contributed to analyze trans-eQTLs.
- A.A.S. contributed to the design, implementation, and validation of cis-eQTL effect size estimation model and empirical Bayes method for multi-tissue eQTL analysis.
- T.C.S. contributed to preliminary colocalization analyses.
- N.A.T. performed initial tissue subsampling and eGene analysis to determine the effect of sample size and tissue number for maximally informative tissues.
- E.K.T. contributed to the design of replication analyses between tissues controlling for the overlap of donors between tissues.
- D.F.C. performed the trans-QTL piRNA enrichment analysis, wrote text and generated the data for Figure 4c.
- E.E. contributed to the development of METASOFT, Meta-Tissue, PM-PLOTS and CAVIAR for multi-tissue eQTL analysis and fine mapping.
- G.L. contributed to the development of an empirical Bayes method for high-tissue eQTL analysis and application to the GTEx data.
- A.B.N. contributed to the design, implementation, and validation of cis-eQTL effect size estimation model and empirical Bayes method for multi-tissue eQTL analysis.

- C.S. developed hierarchical multiple testing procedure (TreeQTL) that was applied to both cis and trans data; wrote text included in main manuscript and supplement describing the procedure and results; contributed to writing the manuscript.
- B.E.S. developed cis-eQTL interaction study design and pipeline, comparison to main effect eQTLs. Contributed to study design and interpretation.
- X.W. contributed to the integrative cis-eQTL analysis incorporating genomic annotations. Contributed to the enrichment analysis of cis-eQTLs in GWAS findings.
- T.L. contributed to and supervised the ASE and effect size analyses and corresponding manuscript sections. Supervised the ASE data creation, pipeline design, QC, and dissemination (data releases, UCSC browser tracks). Contributed to the study design and manuscript reviews and edits.

19.7 Corresponding Authors

- A.B., C.D.B., B.E.E., S.B.M. designed eQTL study, coordinated analyses, and wrote manuscript.

doi:10.1038/nature24277

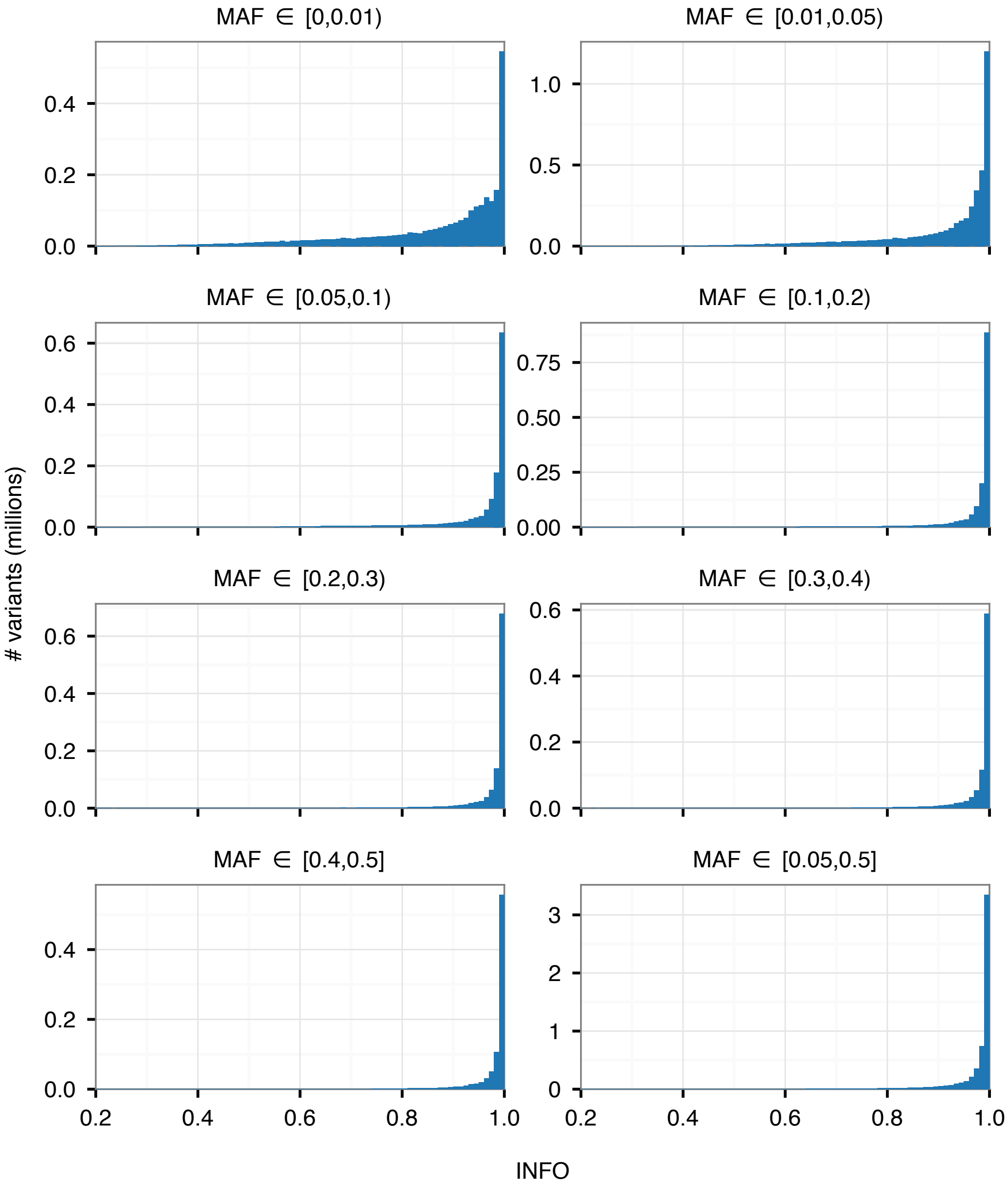

Supplementary Figure 1. Distribution of imputation quality score of autosomes, stratified by minor allele frequency (MAF) for the autosomal imputed variants (variants and indels). The histograms were plotted using all 14,390,153 variants obtained after imputation of 451 samples, before filtering. Bin size: 0.01. INFO: imputation quality score of IMPUTE2.

doi:10.1038/nature24277

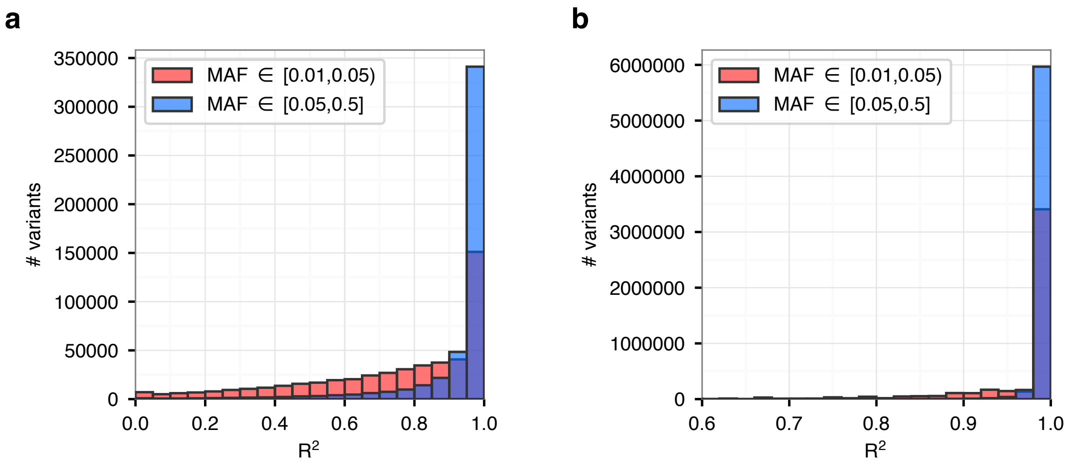

Supplementary Figure 2. Evaluation of imputation accuracy. (a) Comparison of alternative allele dosages between imputed and hard genotype calls across 183 pilot phase samples. R^2 was computed across samples for each variant in the 2.5S set present on Illumina's Omni 5M and absent from the 2.5M array. (b) Comparison of haploid imputed (phased) calls to the standard diploid imputed calls. R^2 was computed between the ALT dosage of the standard imputed variants (continuous variable between 0 and 2) and haploid imputed variants, for each variant across the 450 samples. The R^2 distributions are shown for common variants (blue) and low frequency variants (red). MAF: minor allele frequency.

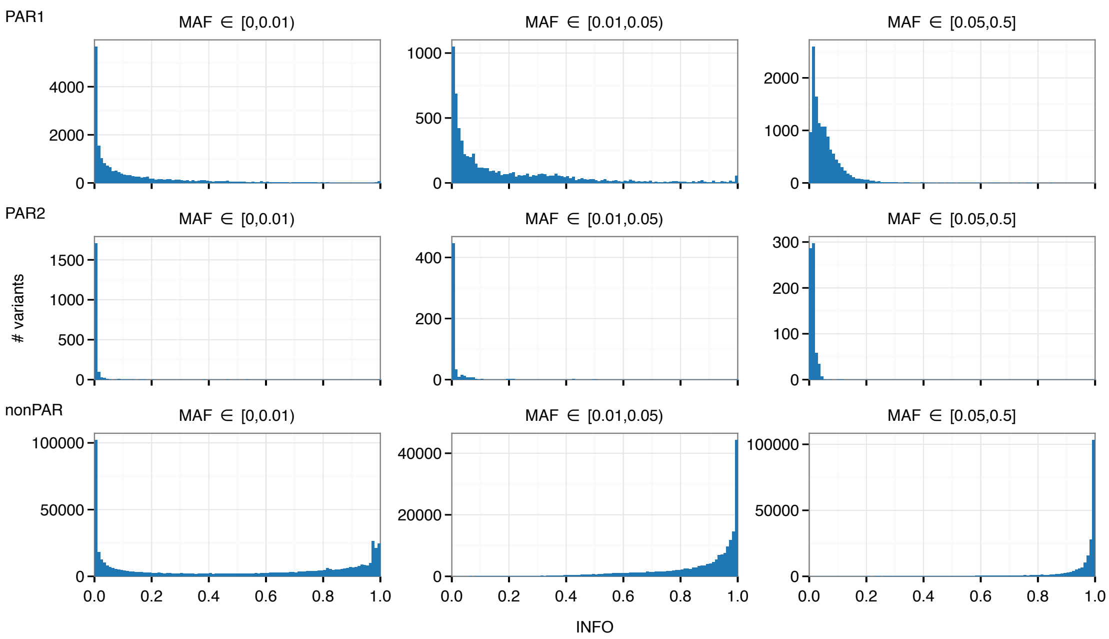

Supplementary Figure 3. Distribution of the IMPUTE2 imputation quality score, INFO, stratified by minor allele frequency (MAF) for the imputed variants on chromosome X for the PAR1, PAR2 and nonPAR regions. The histograms were plotted using all variants obtained after imputation of 451 samples, before QC filtering. Bin size: 0.01.

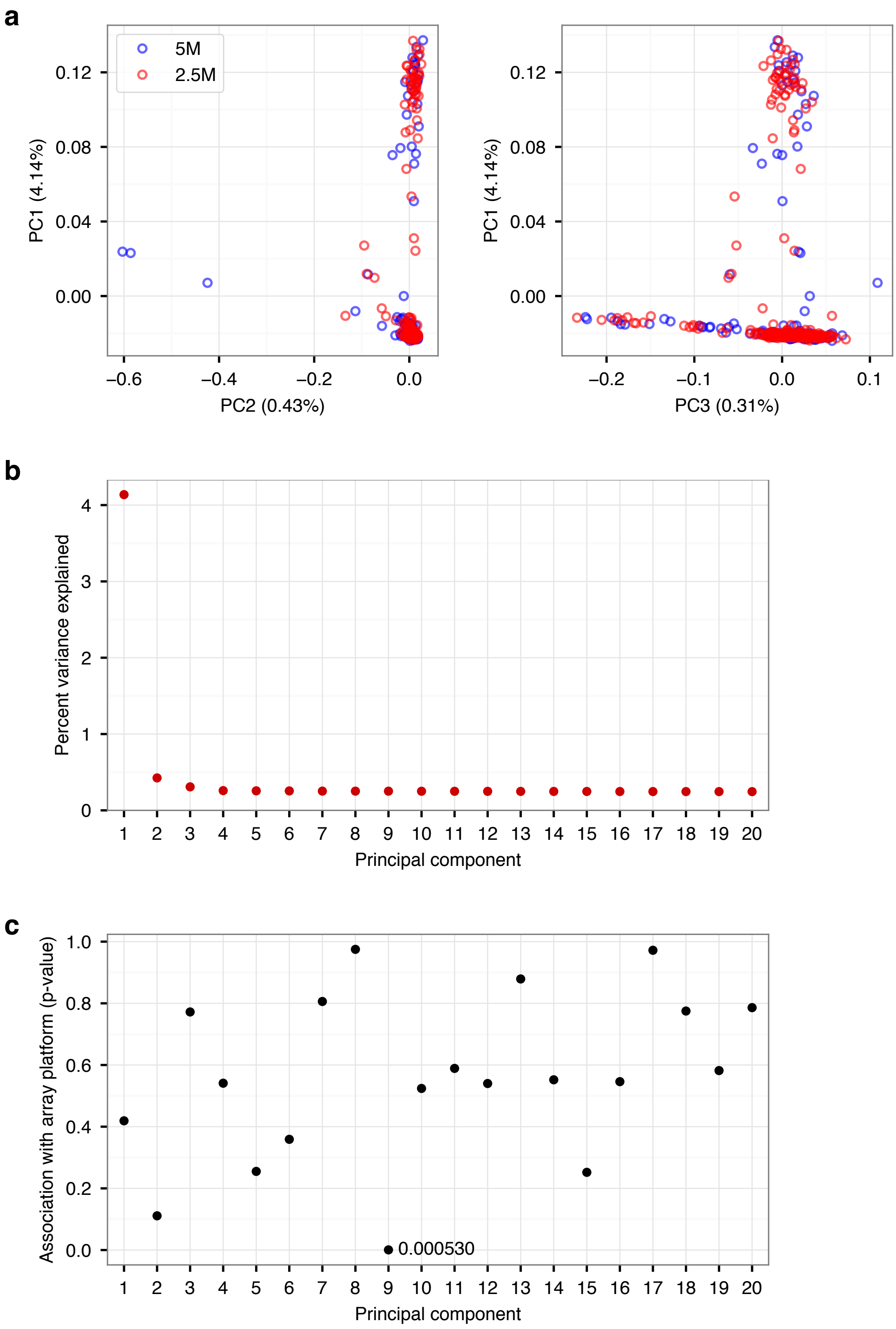

Supplementary Figure 4. Principal component analysis of 451 GTEx samples using a pruned set of variants from the Omni 2.5M set of variants. (a) First three genotype principal components, stratified by array platform (183 samples on Omni 5M array; 168 samples on Omni 2.5M array). (b) Variance explained by first 20 genotype principal components (Eigenvalues). (c) Association of genotype principal components with array platform (Omni 5M vs. 2.5M). Principal components were computed using a pruned set of variants.

doi:10.1038/nature24277

Tissue Site Detail	Abbreviation	Color	Samples from genotyped donors
Adipose - Subcutaneous	ADPSBQ	#FF6600	298
Adipose - Visceral (Omentum)	ADPVSC	#FFAA00	185
Adrenal Gland	ADRNLG	#33DD33	126
Artery - Aorta	ARTAORT	#FF5555	197
Artery - Coronary	ARTCRN	#FFAA99	118
Artery - Tibial	ARTTBL	#FF0000	285
Brain - Anterior cingulate cortex (BA24)	BRNACC	#EEEE00	72
Brain - Caudate (basal ganglia)	BRNCDT	#EEEE00	100
Brain - Cerebellar Hemisphere [Frozen]	BRNCHB	#EEEE00	89
Brain - Cerebellum [PAXgene]	BRNCHA	#EEEE00	103
Brain - Cortex [PAXgene]	BRNCTXA	#EEEE00	96
Brain - Frontal Cortex (BA9) [Frozen]	BRNCTXB	#EEEE00	92
Brain - Hippocampus	BRNHPP	#EEEE00	81
Brain - Hypothalamus	BRNHPT	#EEEE00	81
Brain - Nucleus accumbens (basal ganglia)	BRNNCC	#EEEE00	93
Brain - Putamen (basal ganglia)	BRNPTM	#EEEE00	82
Breast - Mammary Tissue	BREAST	#33CCCC	183
Cells - EBV-transformed lymphocytes	LCL	#CC66FF	114
Cells - Transformed fibroblasts	FIBRBLS	#AAEEFF	272
Colon - Sigmoid	CLNSGM	#EEBB77	124
Colon - Transverse	CLNTRN	#CC9955	169
Esophagus - Gastroesophageal Junction	ESPGEJ	#8B7355	127
Esophagus - Mucosa	ESPMCS	#552200	241
Esophagus - Muscularis	ESPMSL	#BB9988	218
Heart - Atrial Appendage	HRTAA	#9900FF	159
Heart - Left Ventricle	HRTLV	#660099	190
Liver	LIVER	#AABB66	97
Lung	LUNG	#99FF00	278
Muscle - Skeletal	MSCLSK	#AAAAFF	361
Nerve - Tibial	NERVET	#FFD700	256
Ovary	OVARY	#FFAAFF	85
Pancreas	PNCREAS	#995522	149
Pituitary	PTTARY	#AAFF99	87
Prostate	PRSTTE	#DDDDDD	87
Skin - Not Sun Exposed (Suprapubic)	SKINNS	#0000FF	196
Skin - Sun Exposed (Lower leg)	SKINS	#7777FF	302
Small Intestine - Terminal Ileum	SNTTRM	#555522	77
Spleen	SPLEEN	#778855	89
Stomach	STMACH	#FFDD99	170
Testis	TESTIS	#AAAAAA	157
Thyroid	THYROID	#006600	278
Uterus	UTERUS	#FF66FF	70
Vagina	VAGINA	#FF5599	79
Whole Blood	WHLBLD	#FF00BB	338

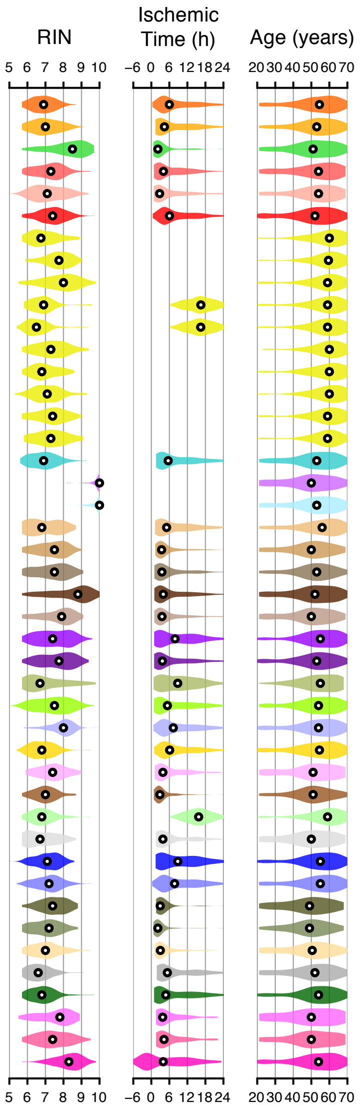

Supplementary Figure 5. Summary of the 44 tissues and 7,051 samples used for eQTL analyses from the GTEx v6p release. Frontal Cortex and Cerebellar Hemisphere were sampled in duplicate: each was sampled on site during initial tissue collection (BRNCHA and BRNCTXA), and again after the brain was received by the brain bank (BRNCHB and BRNCTXB). Two cell types were included: an EBV-transformed lymphoblastoid cell line from blood (LCL) and cultured primary fibroblasts from fresh skin (FIBRBLS). RIN: RNA integrity number. RIN, ischemic time, and donor age distributions for each tissue are shown as density plots, with the median indicated in black.

doi:10.1038/nature24277

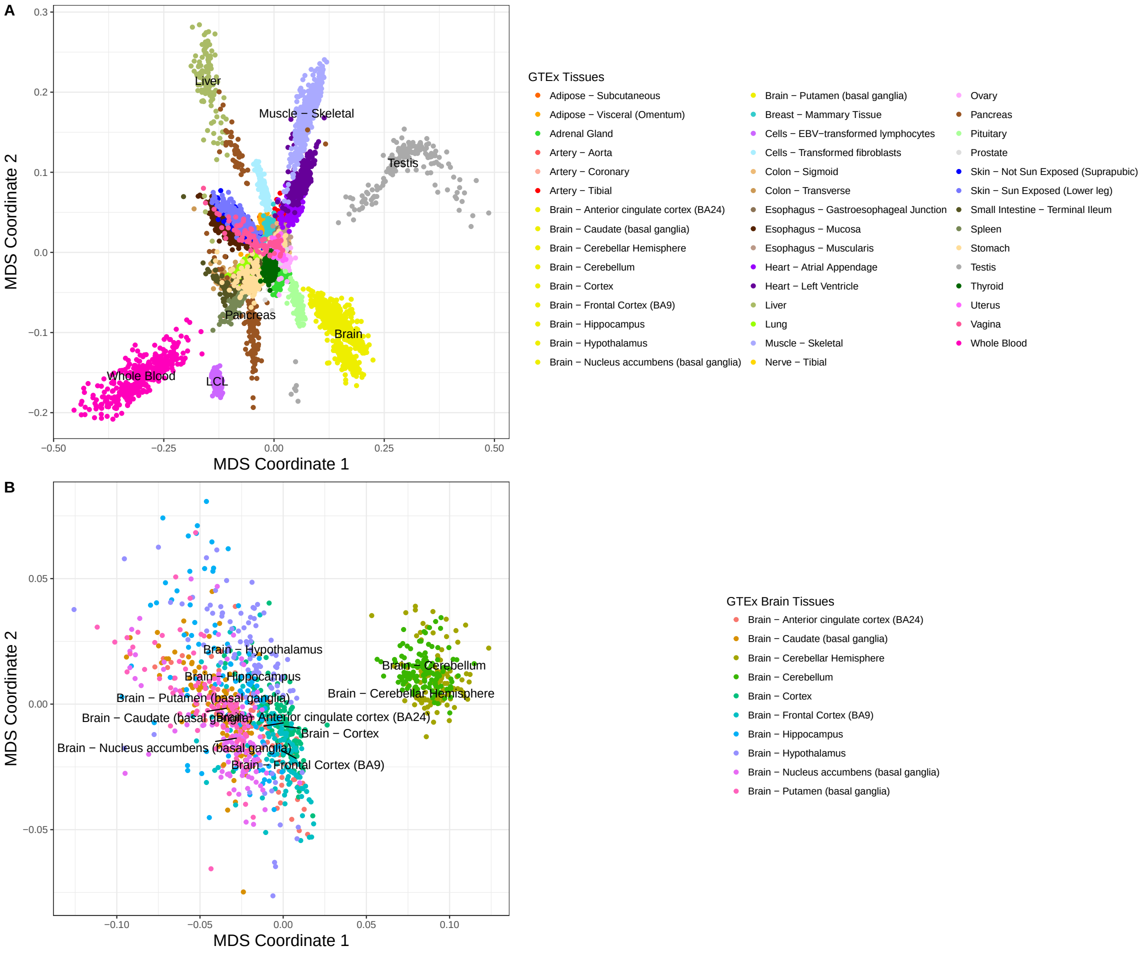

Supplementary Figure 6. MDS plot of tissue transcriptomes. (a) Clustering of all 44 tissues and (b) all brain tissues highlights tissue identities (includes the two duplicate tissues).

doi:10.1038/nature24277

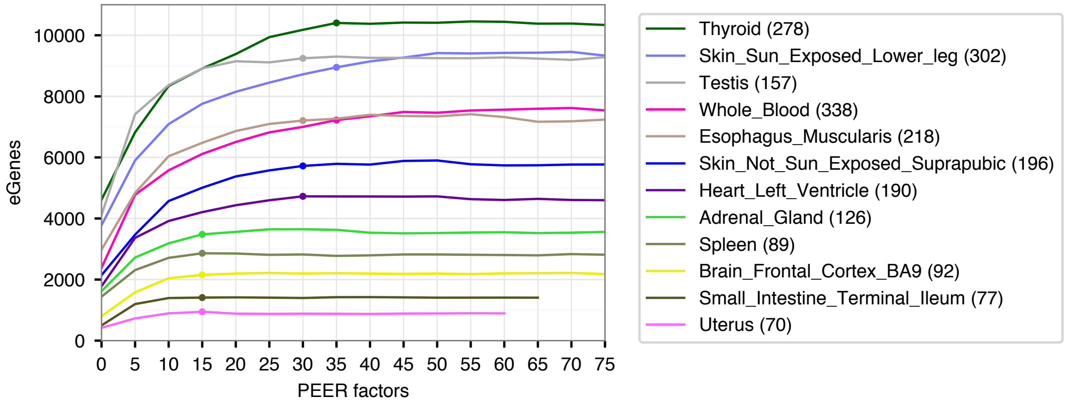

Supplementary Figure 7. Identification of the optimal number of PEER factors for hidden covariate correction during cis-eQTL analyses. The number of PEER factors was chosen to maximize cis-eGene discovery, and this optimization was performed for three sample size bins: tissues with $<$ 150 samples, tissues with $\geq$150 and $<$250 samples, and tissues with $\geq$250 samples available. The cis-eQTL discovery pipeline was run with increments of 5 PEER factors for the 12 tissues shown, using a reduced number of permutations (100 instead of the adaptive 1,000-10,000 used for all other analyses). Based on these results and to avoid potential overfitting, 15, 30, and 35 PEER factors were selected, respectively.

doi:10.1038/nature24277

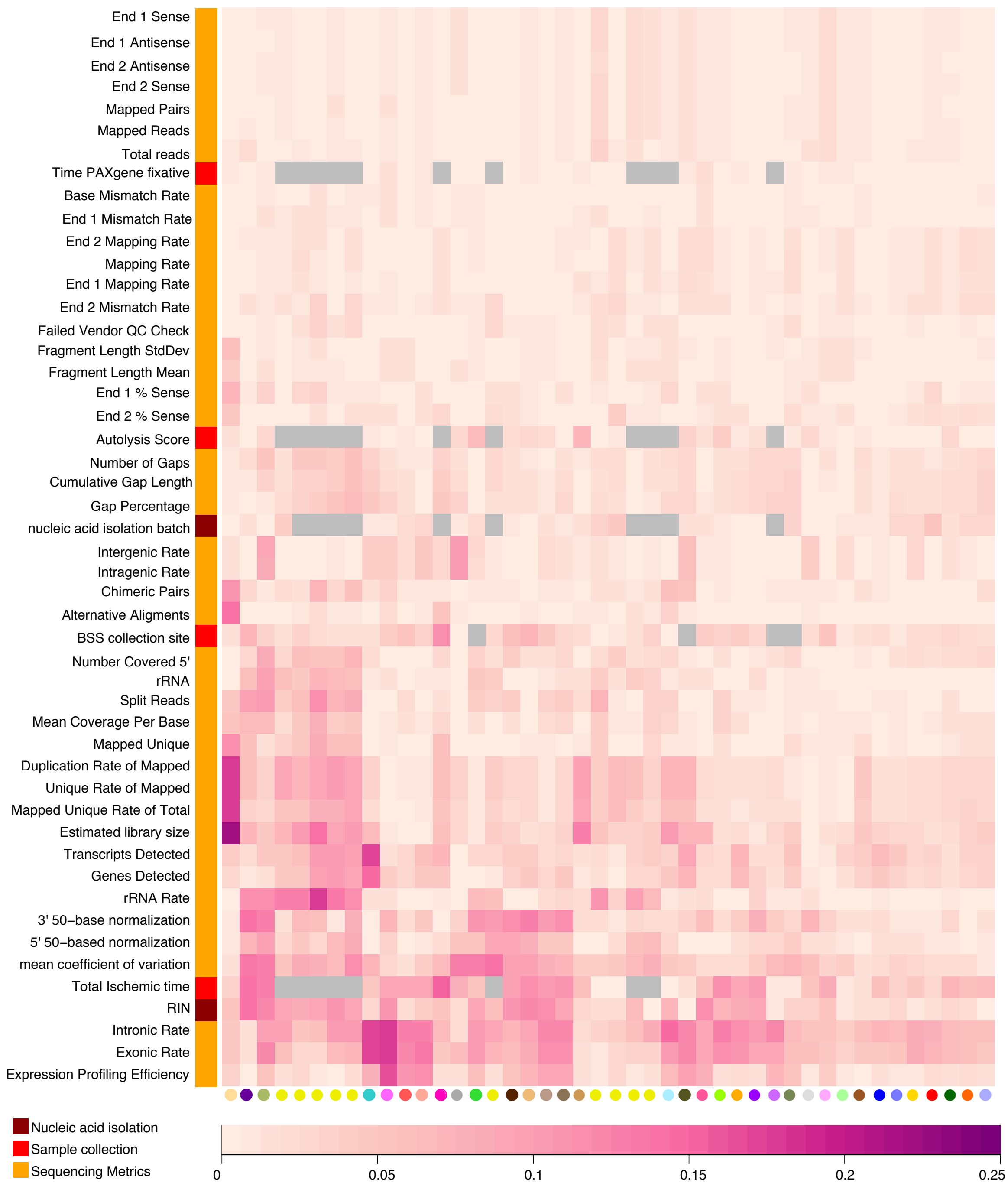

Supplementary Figure 8. Sample covariates associated with PEER factors in each tissue. For each tissue, adjusted (R^2) reflecting the proportion of variance explained by each sample-specific covariate, for the entire PEER component removed from the expression data. Each cell reflects variance explained for a tissue/covariate pair, color scale at bottom. Grey cells represent pairs with insufficient data for estimation.

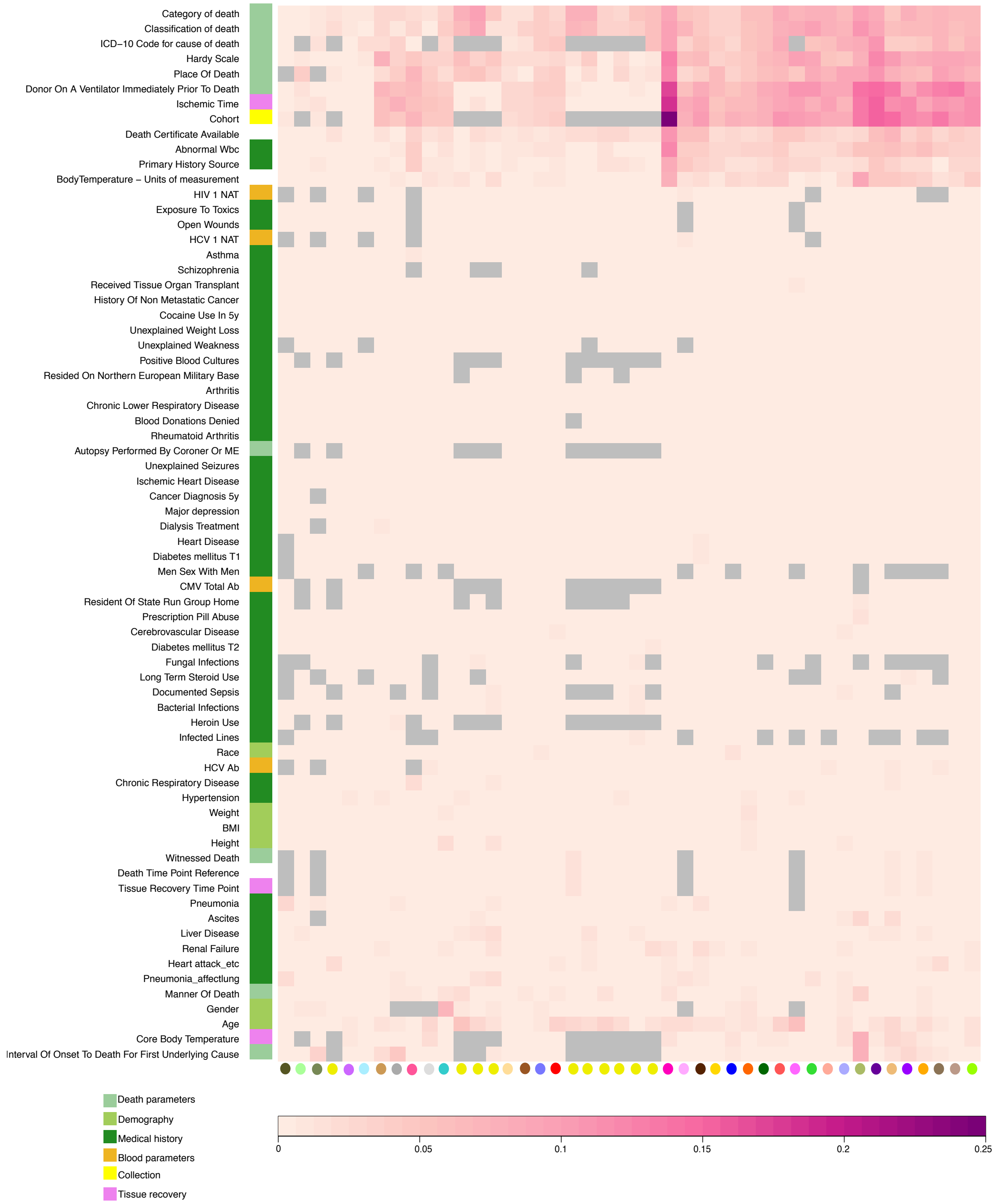

Supplementary Figure 9. Donor covariates associated with PEER factors in each tissue. For each tissue, adjusted (R^2) reflecting the proportion of variance explained by each donor-specific covariate, for the entire PEER component removed from the expression data. Each cell reflects variance explained for a tissue/covariate pair, color scale at bottom. Grey cells represent pairs with insufficient data for estimation.

a

Tissue	Significant genome-wide assoc. with PEERs (FDR≤0.05)
Adipose - Subcutaneous	0
Adipose - Visceral (Omentum)	0
Adrenal Gland	0
Artery - Aorta	0
Artery - Coronary	0
Artery - Tibial	0
Brain - Anterior cingulate cortex (BA24)	0
Brain - Caudate (basal ganglia)	0
Brain - Cerebellar Hemisphere	0
Brain - Cerebellum	0
Brain - Cortex	0
Brain - Frontal Cortex (BA9)	0
Brain - Hippocampus	0
Brain - Hypothalamus	0
Brain - Nucleus accumbens (basal ganglia)	0
Brain - Putamen (basal ganglia)	0
Breast - Mammary Tissue	197
Cells - EBV-transformed lymphocytes	0
Cells - Transformed fibroblasts	2
Colon - Sigmoid	0
Colon - Transverse	0
Esophagus - Gastroesophageal Junction	0
Esophagus - Mucosa	0
Esophagus - Muscularis	0
Heart - Atrial Appendage	0
Heart - Left Ventricle	0
Liver	0
Lung	0
Muscle - Skeletal	0
Nerve - Tibial	4
Ovary	0
Pancreas	0
Pituitary	0
Prostate	0
Skin - Not Sun Exposed (Suprapubic)	0
Skin - Sun Exposed (Lower leg)	1
Small Intestine - Terminal Ileum	0
Spleen	0
Stomach	0
Testis	0
Thyroid	0
Uterus	0
Vagina	0
Whole Blood	0

b

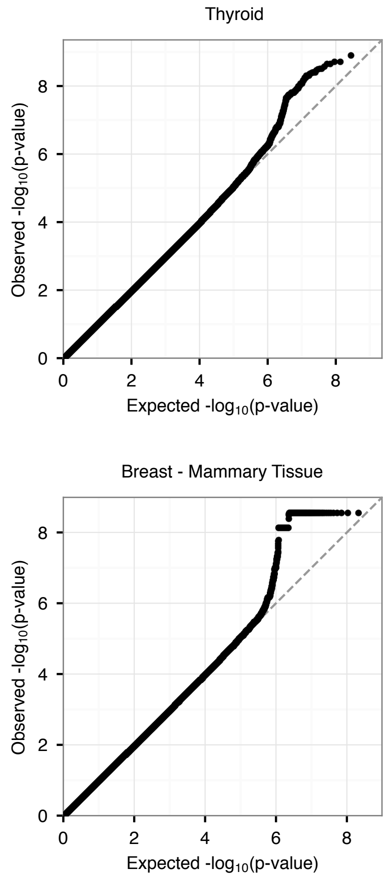

c

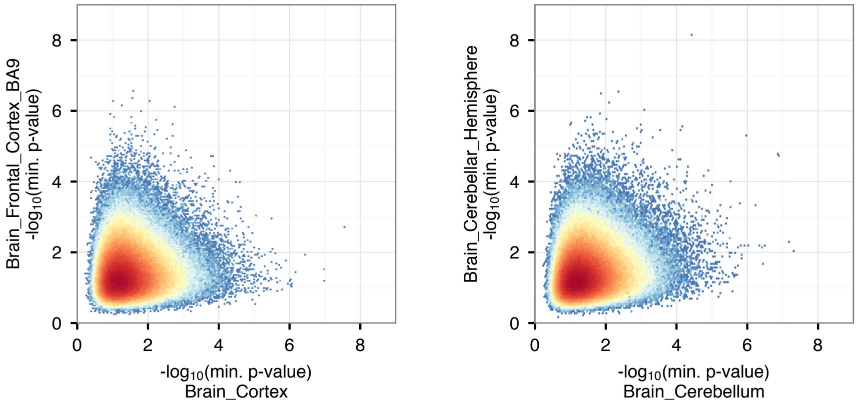

Supplementary Figure 10. Genome-wide association with PEER factors. (a) Number of significant associations of genetic variants genome-wide with PEER factors, across all factors (FDR $\geq$ 0.05; Benjamini-Hochberg). Associations were calculated for each factor using PLINK, with the options "--linear hide-covar --adjust --keep-allele-order --maf 0.01 --min-ac 10 --geno 0.05", and genomic-control corrected P-values were used. The top three genotype PCs, sex, and genotyping platform were included as covariates. (b) Quantile-quantile plots for Thyroid and Breast – Mammary tissues, computed across all PEER factors. (c) Comparison of association P-values between replicate tissues (PAXgene vs. frozen).

doi:10.1038/nature24277

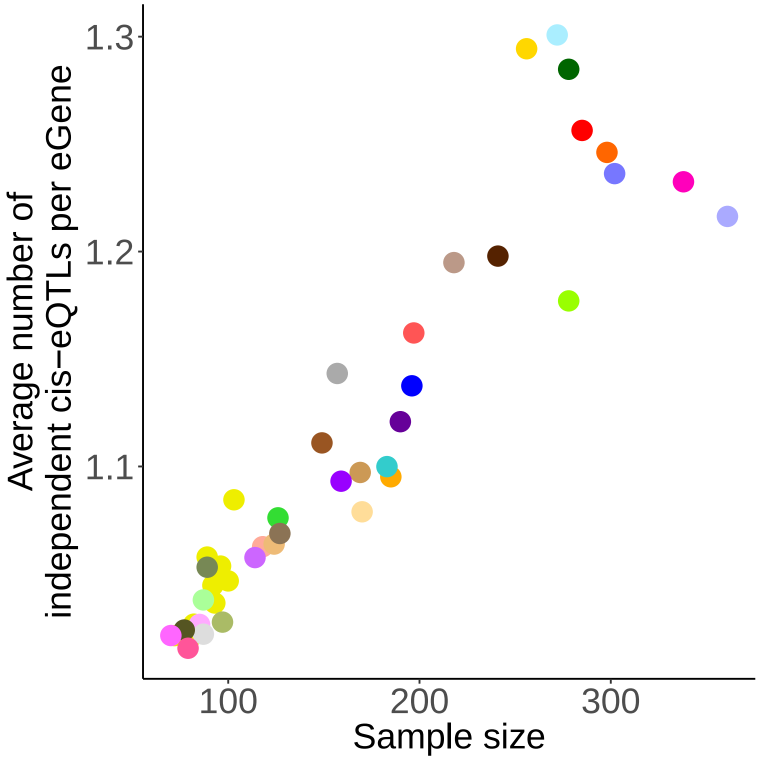

Supplementary Figure 11. Conditionally independent cis-eQTL discovery as a function of sample size. Mirroring cis-eGene discovery, independent cis-eQTL discovery increases as a function of sample size, recovering up to 1.3-fold more cis-eQTLs per tissue.

doi:10.1038/nature24277

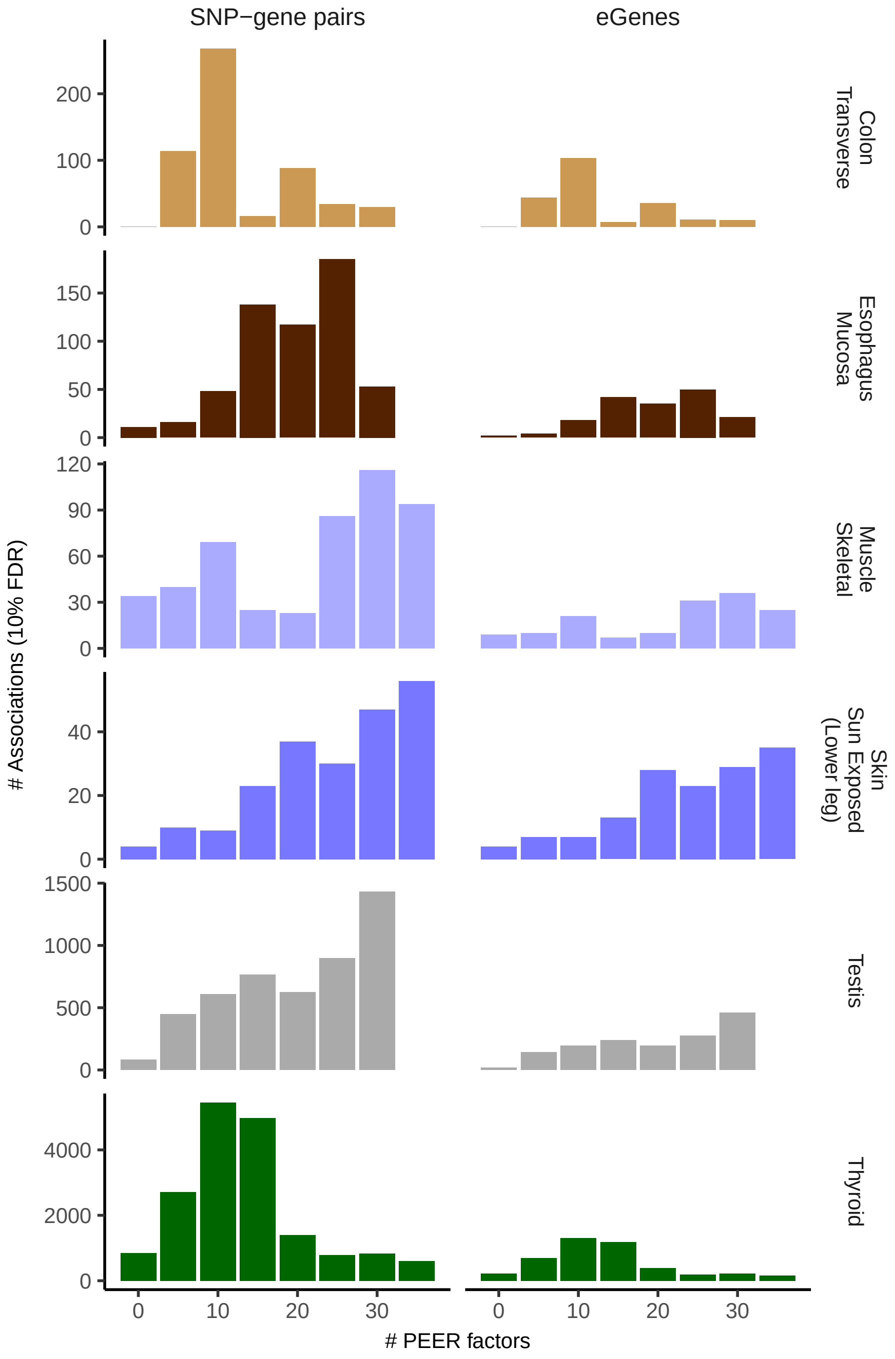

Supplementary Figure 12. Number of trans-eQTLs and trans-eGenes as a function of PEER factors for six tissues. The number of trans-eQTLs and trans-eGenes are plotted as a function of PEER factors (increments of 5), for the following tissues: **(a)** esophagus mucosa, **(b)** skeletal muscle, **(c)** transverse colon, **(d)** thyroid, **(e)** sun exposed skin, and **(f)** testis. Different tissues show very different, and often not smoothly varying or monotonically increasing, numbers of trans-eQTLs identified with different numbers of PEER factors removed, as opposed to cis-eQTLs, which show a more consistent pattern. Along with the fact that we have insufficient statistical power or number of trans-eQTLs to tune the number of PEER factors directly without facing potential over-fitting to spurious signal, this figure shows the challenges of controlling for unobserved confounders in trans-eQTL study and supports our reasoning for not tuning the number of PEER factors directly for trans-eQTLs.

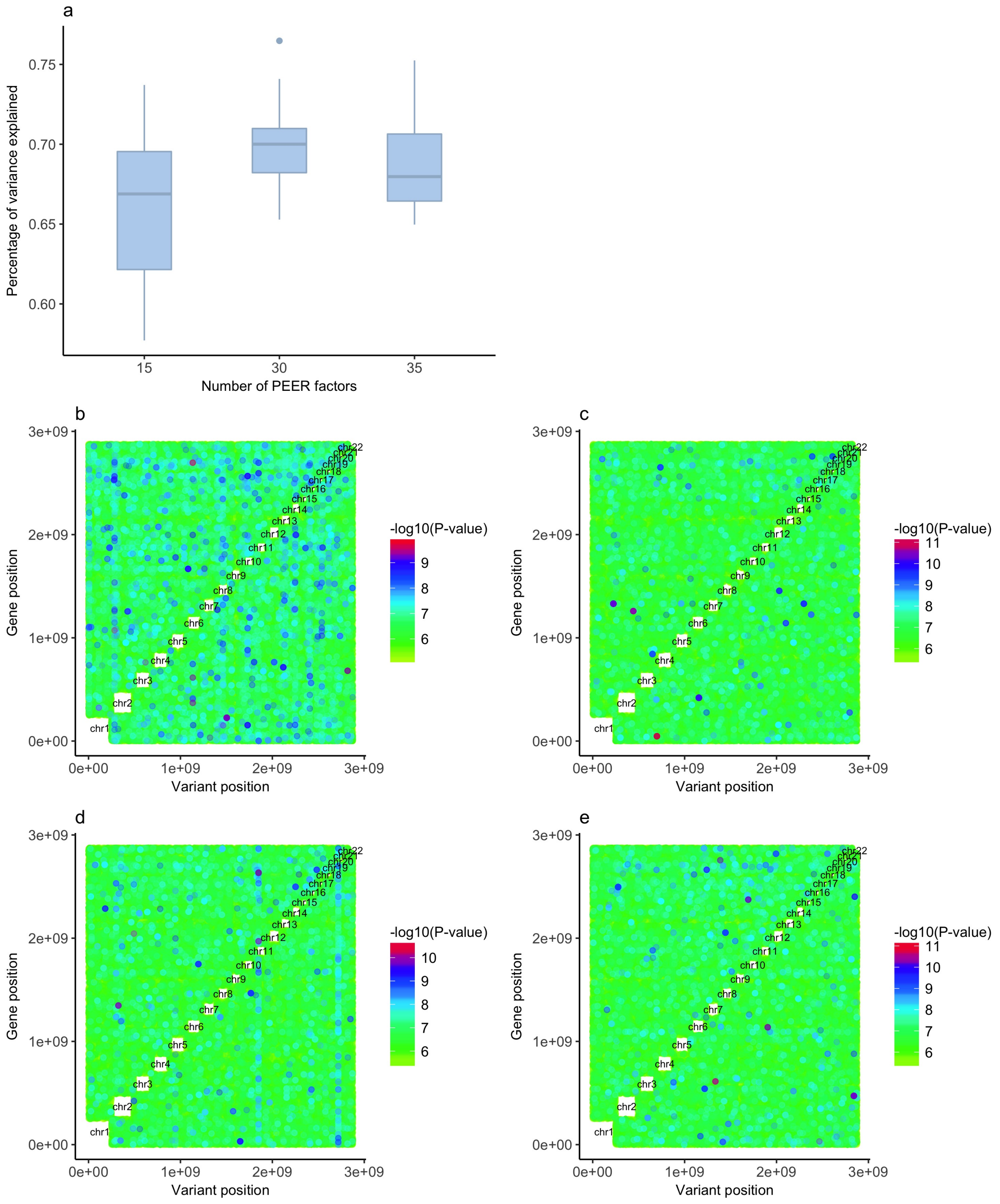
a
Percentage of variance explained
Number of PEER factors
15
30
35
b
c
d
e
Gene position
Variant position
-log10(P-value)
chr1
chr2
chr3
chr4
chr5
chr6
chr7
chr8
chr9
chr10
chr11
chr12
chr13
chr14
chr15
chr16
chr17
chr18
chr19
chr20
chr21
chr22

Supplementary Figure 13. PEER correction removes broad expression trends affecting trans-eQTL discovery. **(a)** Distribution of the variance explained (across all genes) by the PEER factors used as covariates in trans-eQTL association testing in each of the 44 tissues, grouped by the number of PEER factors used for each tissue (only 15, 30, or 35 PEER factors were used as covariates in eQTL testing depending on tissue sample size; see Online Methods). **(b-c)** Location and strength ($-\log_{10}$(P-value)) of trans associations in vagina, quantified based on expression data (b) before and (c) after PEER correction. Each point is a trans-eQTL variant-gene pair where the x-axis is the variant location and the y-axis is the gene TSS. Data points are colored according to $-\log_{10}$(P-value) of association testing. Vertical bands reflect loci with numerous targets in the data before PEER factor correction. These vertical bands disappear post PEER correction due to the stringent condition of applying the same amount of PEER correction as the cis-eQTL mapping. The same plot is replicated for stomach, **(d)** before and **(e)** after PEER correction. Box plots depict the IQR, whiskers depict 1.5 x IQR.

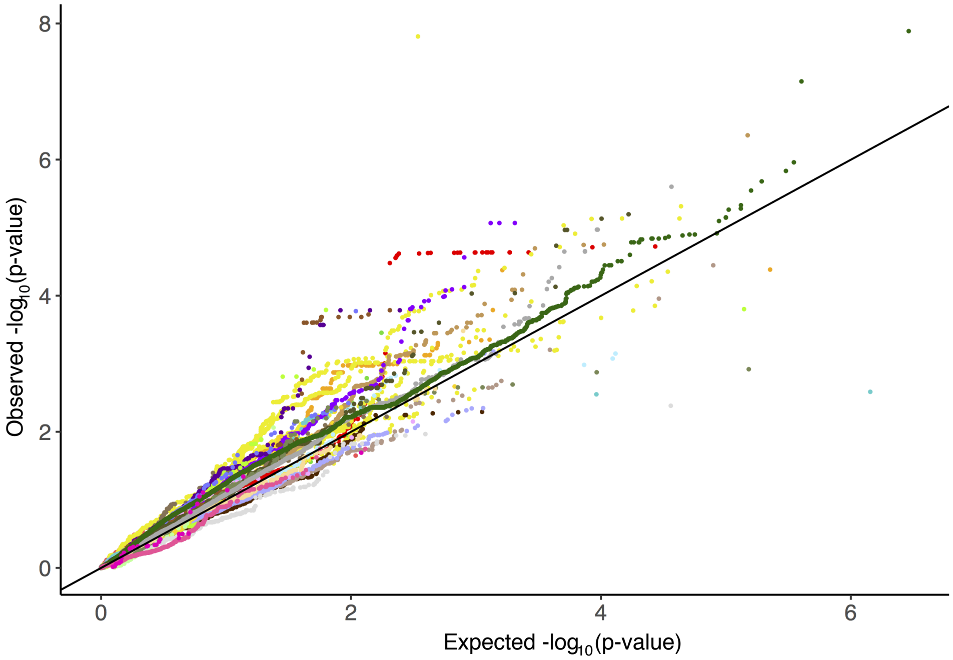

Supplementary Figure 14. Trans-eVariants lost after PEER correction are enriched for association with known covariates. Trans-eVariants that were detected in raw expression data but lost after PEER correction were tested for association with known sample covariates using a linear model. This quantile-quantile plot shows $-\log_{10}$(P-values) of trans-eVariants lost after PEER correction as compared to matched random variants, with each tissue shown as a distinct color. Combined across tissues, the association $-\log_{10}$(P-values) are significantly larger than random (Wilcoxon rank sum test; P $\leq 2.2 \times 10^{-16}$).

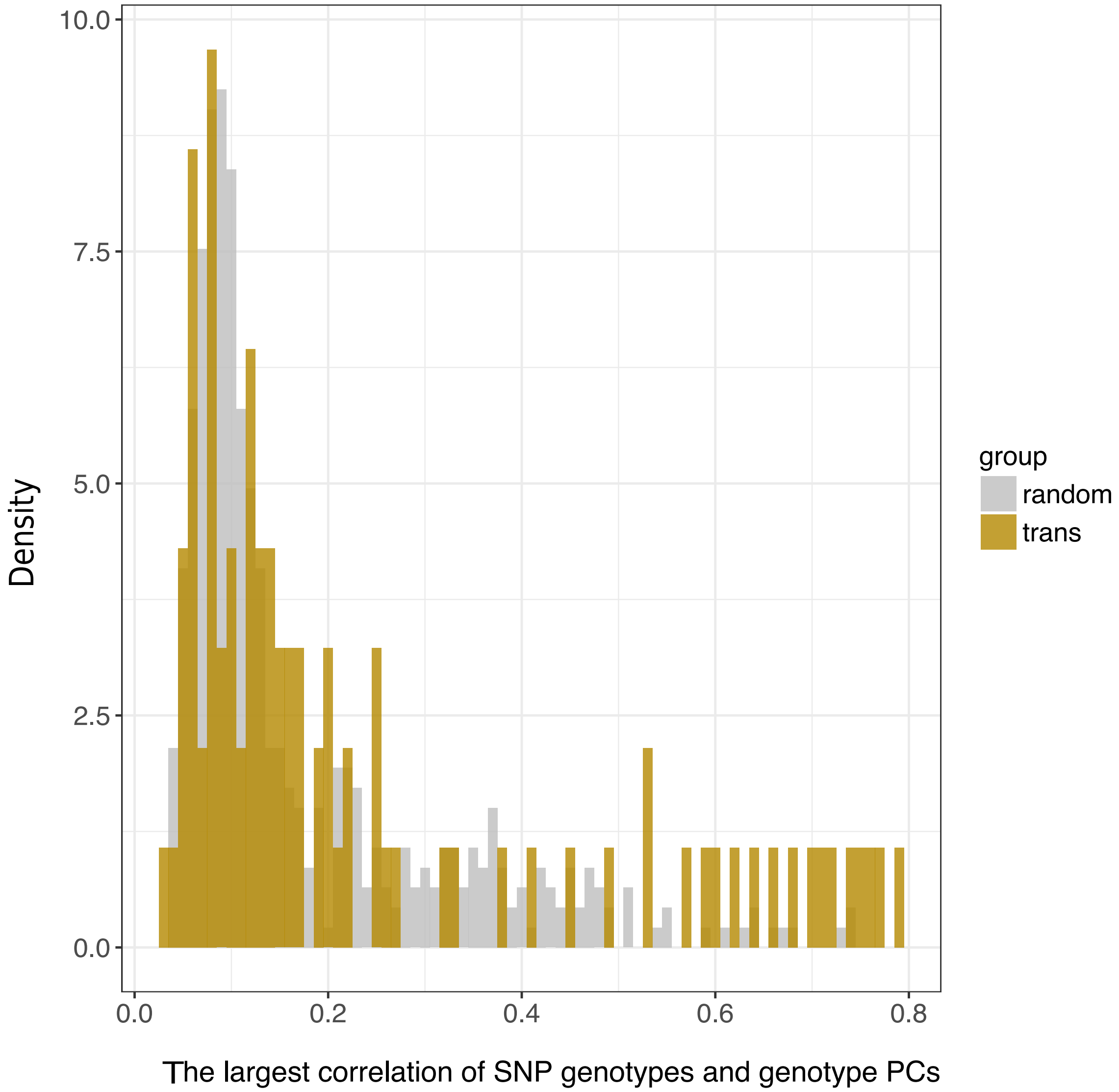

Supplementary Figure 15. Correlation between trans-eVariants and genotype principal components. Distribution of the largest correlation between the top 20 genotype principal components and both the 93 trans-eVariants (single top variant per eGene) and 465 randomly selected variants matched for MAF. The two distributions are significantly different, with the trans-eVariants being enriched for higher correlation with genotype PCs (Wilcoxon rank sum test, $P \leq 0.029$) .

doi:10.1038/nature24277

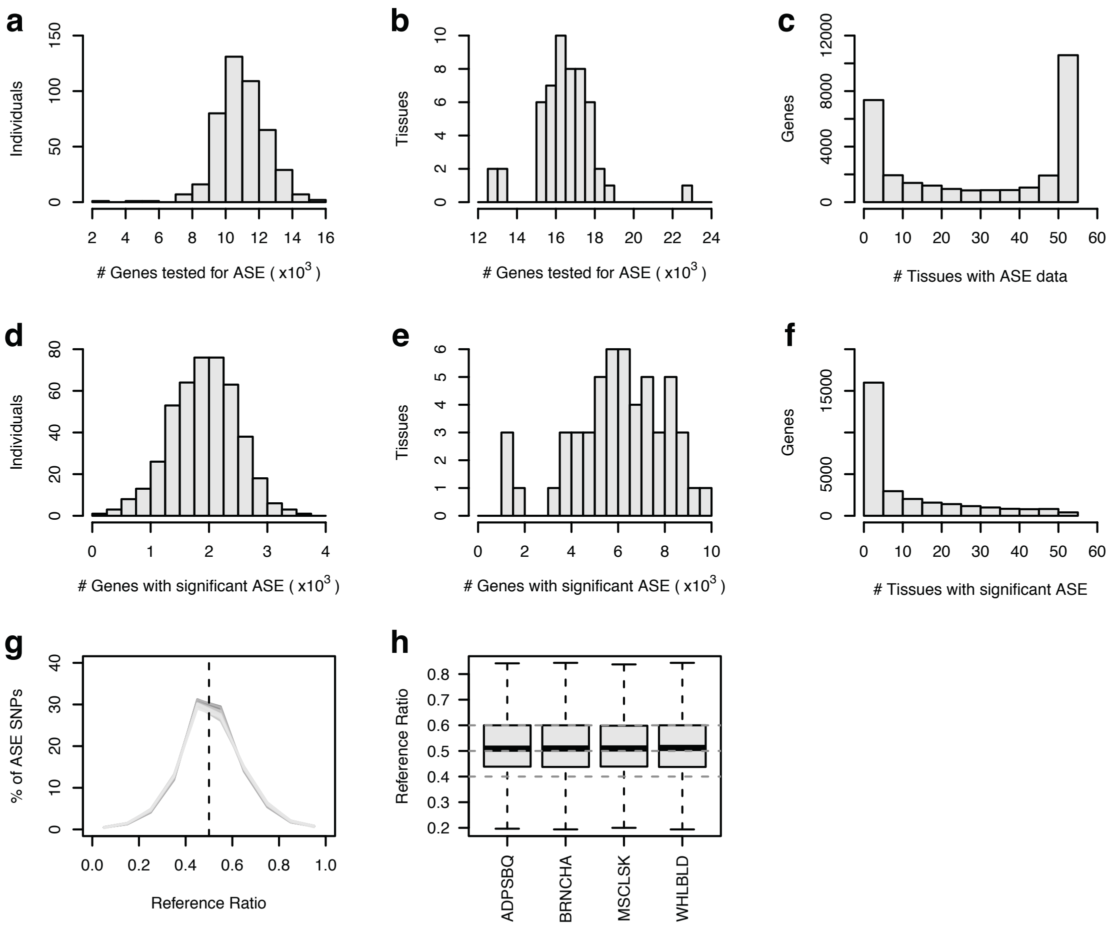

Supplementary Figure 16. Global ASE statistics. (a) Histogram of the number of genes with tested ASE variants per donor. (b) Histogram of the number of genes with tested ASE variants per tissue. (c) Histogram of the number of tissues with tested ASE variants per gene. (d) Histogram of the number of genes with significant (binomial test versus 0.50, 5% FDR) ASE variants per donor. (e) Histogram of the number of genes with significant (binomial test versus 0.50, 5% FDR) ASE variants per tissue. (f) Histogram of the number of tissues with significant (binomial test versus 0.50, 5% FDR) ASE variants per gene. (g) Distribution of reference ratios (reference allele count / (reference + alternative allele count)) at ASE variants across all tissues. (h) Boxplot of reference ratios at ASE variants for four tissues (APSBQ = adipose – subcutaneous, BRNCHA = brain – cerebellum, MSCLSK = muscle – skeletal, WHLBD = whole blood). Gene level measurements of haplotype expression were calculated by aggregating counts per sample across all heterozygous variants with ASE data within the gene using population phasing. The following filters were applied on ASE data: total coverage $\geq$ 8 reads, no mapping bias in simulations [42], UCSC mappability $>$ 50, and no significant ($P < 0.01$) evidence that variant is monoallelic in expression data [43]. Box plots depict the IQR, whiskers depict 1.5 x IQR.

doi:10.1038/nature24277

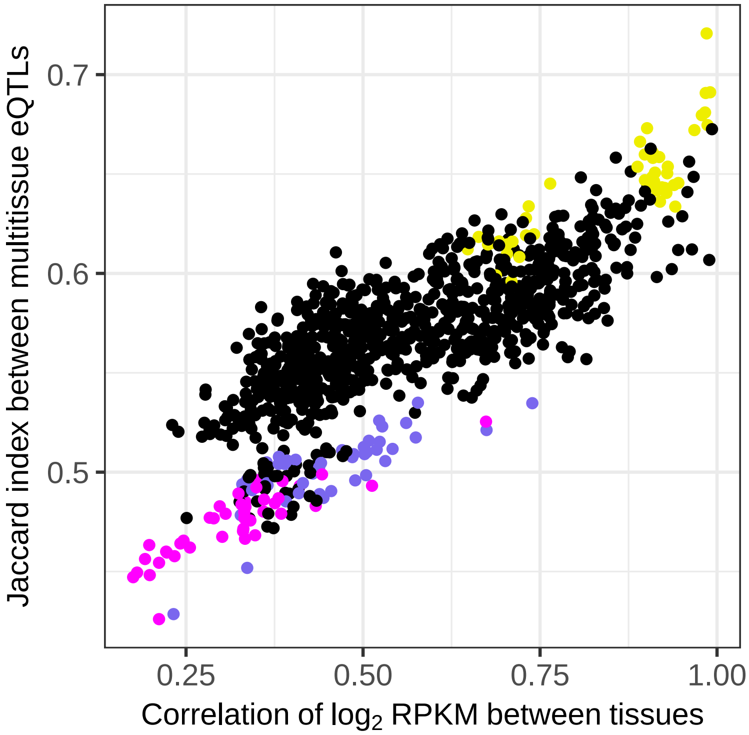

Supplementary Figure 17. Tissues with similar expression patterns are more likely to share cis-eQTLs. For each gene, we calculated the median RPKM across the 70 donors used in the subsampling analyses in each tissue. Pearson r^2 values were then calculated in a pairwise fashion between tissues on $\log_2$-transformed median RPKM values, using only genes expressed in both tissues with a significant multitissue eQTL identified by the subsampled Meta-Tissue analysis. We then measured the sharing of multitissue eQTLs between each pair of tissues using the Jaccard index, where each eQTL was active in a tissue if it had an m-value > 0.9. Each point represents a comparison between two of the 44 tissues: comparisons between brain tissues are shown in yellow; comparisons with skeletal muscle are shown in purple; comparisons with whole blood are shown in magenta.

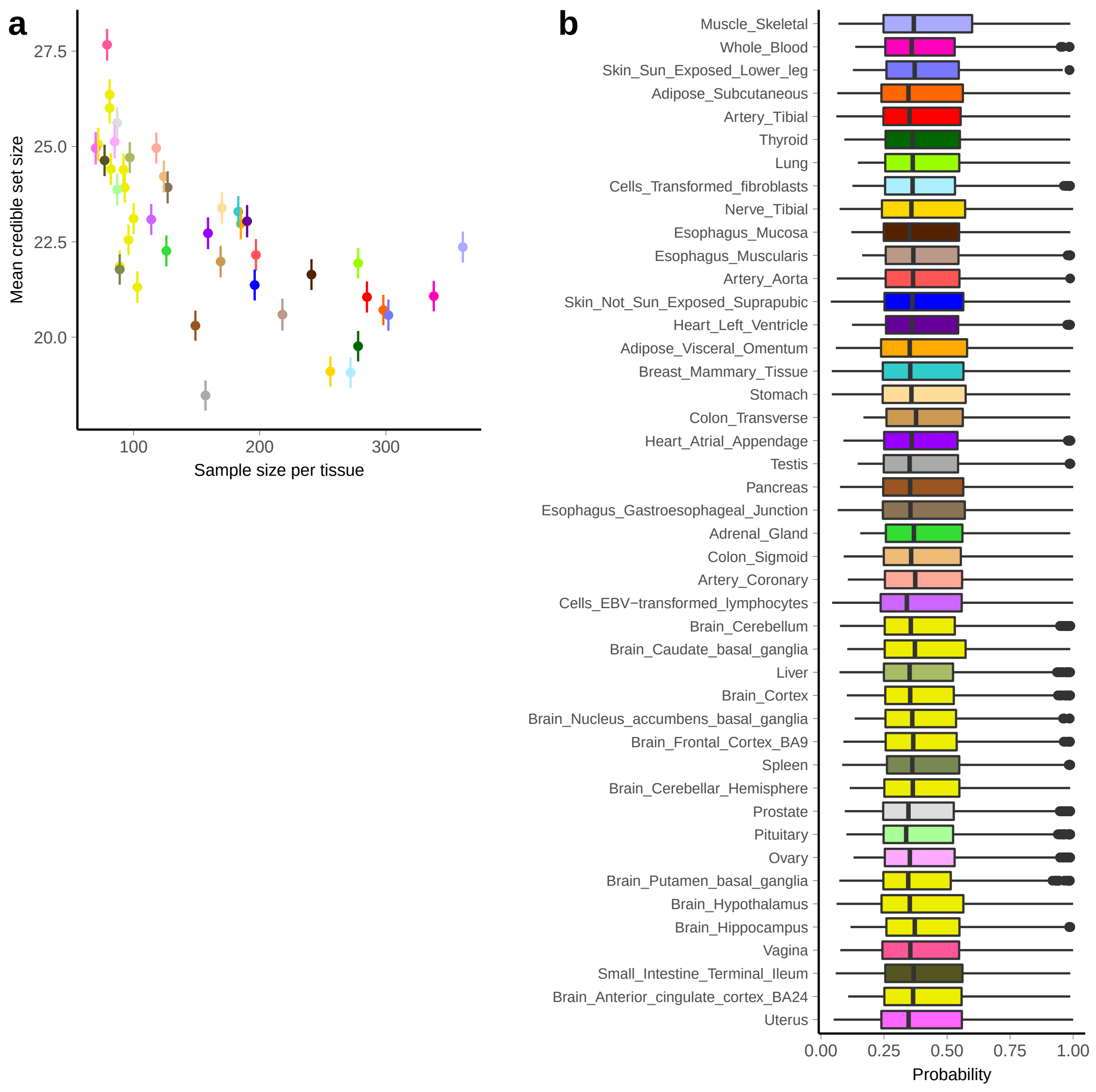

Supplementary Figure 18. Fine-mapping of cis-eQTLs. (a) Mean 90% credible set size (and its standard error) is plotted for the top 1,000 cis-eQTLs against the tissues sample size. **(b)** Distributions of CaVEMaN probabilities that lead cis-eQTLs for each tissue are causal variants (boxplots; x-axis) for each tissue (y-axis, by color). Tissues are sorted in decreasing order of sample size from top to bottom. Box plots depict the IQR, whiskers depict 1.5 x IQR.

Supplementary Table 1. Sample and variant Quality Control (QC) of merged Illumina Omni 5M and Omni 2.5M genotypes, pre-imputation. The Omni 5M or 2.5M genotypes of 455 GTEx donors were combined by merging the overlapping 2.5M set of variants. 450 donors remained after sample QC (excluding individuals with Klinefelter syndrome, related donors, replicate samples and a chr17p trisomy donor). The Hardy-Weinberg Equilibrium (HWE) test was performed using donors of European descent only (N= 378). The predicted genotype missingness using surrounding haplotypes was performed using the PLINK command: `"--test-mishap --fisher"`. The testing of association with chemistry plate batch was performed using the PLINK command: `"--loop-assoc --fisher"`. Note that the sum of variants removed or retained exceeds the starting number of variants, due to partial overlap between variants from the different QC steps.

QC Steps	# Unique GTEx individuals	# Flagged samples	# Variants kept	# Variants removed
**Pre-array Merging QC**				
**Original data**	455	-	2,307,617	-
1. Allele frequency association test between samples on Omni 5M and Omni 2.5M	455	-	2,307,615	2 (rs2300699, rs4961559)
2. Remove replicate variants with the same chromosome position but different variant IDs	455	-	2,307,261	354
3. Variants whose REF and ALT alleles did not align between Omni 2.5M and Omni 5M	455	-	2,307,260	1 (rs2040962)
4. Remove strand ambiguous variants (A/T, C/G), as many misaligned between the arrays	455	-	2,236,981	70,267
**Post-array Merging, Pre-imputation QC**				
**Merged Omni 5M and 2.5M samples**	455	-	2,236,981	-
5. Exclude variant genotyping call rate < 95%	455	-	2,193,089	43,892
**Sample QC steps**				
6. Exclude Individuals with call rate < 98%	455	-	2,193,089	-
7. Sex check (Chr X heterozygosity test)	454	1 donor	2,193,089	-
8. Heterozygosity test (per sample)	454	-	-	-
9. Genome identity-by-descent (IBD)	454	-	2,193,089	-
Sample contamination	454	-	2,193,089	-
Cryptic relationships (Pi_HAT>0.1875)	454	3 donors	2,193,089	-
Sample duplicates	451	-	2,193,089	-
10.Exclude monomorphic variants	451	-	2,013,620	179,469
11. Exclude variants with call rate < 98%	451	-	1,931,034	82,586
12. Exclude variants with differential missingness between Omni 2.5M and 5M arrays (miss> 0.02 with $P < 0.05$)	451	-		89,411
13.Variant-level tests				
Testing HWE (p< 1×10^{-6})	451	-		279
Genotype missingness predicted using surrounding haplotypes (p< 1×10^{-8})	451	-		2,710
Testing for association with chemistry plate batch (P< 1×10^{-8})	451	-		11,613
Variants with heterozygous haploid genotypes on sex chromosomes in males	451	-		636
**SubTotal**	**451**	-	**1,883,274**	**309,815**
**Total**	**451**	4	**1,883,274**	**424,343**
**Post-Imputation sample QC**				
Exclude chr17 trisomy donor (GTEX-UPIC; identified after imputation performed)	451	1		-
**Total**	**450**	-	**1,883,274**	-

Number of variants that overlapped between the Omni 2.5M and 5M arrays.

12 variants were lost during format conversion from VCF to .tped using VCFtools.

One donor with Klinefelter syndrome genotyped on Omni 2.5M was removed; 2 additional Klinefelter donors were previously removed from the Pilot phase analysis freeze.

One donor from a related pair and two donors from a related trio were removed based on an identity-by-descent (IBD) value of $\hat{\pi} > 0.1875$ that represents a relationship between third- and second-degree relatives. Genome-wide variants were used for the IBD calculation.

doi:10.1038/nature24277

Supplementary Table 2. Concordance of minor allele dosage between hard calls and imputed genotypes across 183 GTEx donors genotyped on Omni 5M array using the 2.5S portion of variants (c. 1.8 million). For concordance analysis, the Omni 5M genotypes were downsized to the 2.5M set of the array, and the 2.5S variants were imputed back into the samples, using 1000 Genomes Phase I v3 as the reference panel.

INFO (in OMNI 2.5M)	MAF	Mean R^2	Median R^2	# Variants
	5-50%	0.9364	0.9861	450,178
> 0.4	1-5%	0.72	0.7979	552,807
	<1%	0.6198	0.6648	336,005
	5-50%	0.9713	0.9893	405,031
> 0.9	1-5%	0.9073	0.9717	273,847
	<1%	0.8499	0.9994	118,170

Supplementary Table 3. Average imputation quality score (INFO) of autosomal variants stratified by minor allele frequency (MAF). INFO was computed using IMPUTE2. SNPs and indels were tested. The values were computed using all 14,390,153 variants obtained after imputation of 451 samples, before applying any variant filters.

MAF	Mean INFO	Median INFO	# variants
0-0.01	0.860	0.925	2,658,149
0.01-0.05	0.888	0.951	6,990,740
0.05-0.1	0.947	0.989	1,306,106
0.1-0.2	0.962	0.993	1,532,517
0.2-0.3	0.969	0.994	1,107,100
0.3-0.4	0.971	0.994	946,939
0.4-0.5	0.972	0.994	893,237
0.05-0.5	0.963	0.993	5,785,899

doi:10.1038/nature24277

Supplementary Table 4. Assessment of imputation accuracy post-variant QC. Concordance between imputed and directly called genotypes from 183 GTEx Pilot phase samples out of 450 imputed samples was computed, using the 2.5S set of variants present only on the Omni 5M array. Mean and median concordance values (R^2) were computed for common (MAF$= 0.05 - 0.5$) and low frequency (MAF$= 0.01 - 0.05$) 2.5S variants, separately, and for incremental IMPUTE2 INFO cutoffs starting with INFO> 0.4. Discrete values 0,1,2 were used for the hard calls, and continuous variables for the imputed calls. INFO: measure of imputation confidence computed by IMPUTE2. MAF: minor allele frequency.

A. $0.05 \leq$ MAF ≤ 0.50

IMPUTE2 INFO	Mean R^2	Median R^2	# Variants tested
>0.4	0.9313	0.9854	474,204
>0.5	0.9325	0.9854	473,490
>0.6	0.9357	0.9856	471,359
>0.7	0.9423	0.9861	465,920
>0.8	0.9532	0.9871	453,384
>0.9	0.9688	0.9891	423,106

B. $0.01 \leq$ MAF ≤ 0.05

IMPUTE2 INFO	Mean R^2	Median R^2	# Variants tested
>0.4	0.7226	0.8037	571,662
>0.5	0.7298	0.8092	564,329
>0.6	0.7495	0.8254	541,678
>0.7	0.7862	0.8552	492,978
>0.8	0.8398	0.9047	408,387
>0.9	0.9061	0.9764	279,918

Supplementary Table 5. Chromosome coordinates of PAR1, nonPAR and PAR2 regions on chromosome X. Coordinates were taken from UCSC browser (hg19). PAR: pseudoautosomal region; nonPAR: non-pseudoautosomal region.

Chr X region	Chromosome start position (bp; hg19)	Chromosome end position (bp; hg19)	Interval size (Mb)
PAR1	60,001	2,699,520	2.64
nonPAR	2,699,521	154,931,043	152.23
PAR2	154,931,044	155,260,560	0.33

doi:10.1038/nature24277

Supplementary Table 6. Average imputation quality score (INFO) stratified by minor allele frequency (MAF) for PAR1, PAR2 and nonPAR regions on chromosome X. INFO was computed using IMPUTE2. Variants tested include SNPs and indels. The values were computed using all variants obtained after imputation of 451 samples, before applying any filters.

chr X region	MAF	Mean INFO	Median INFO	# variants
PAR1	0-0.01	0.138	0.05	19,909
	0.01-0.05	0.175	0.076	6,226
	0.05-0.5	0.059	0.041	12,975
PAR2	0 - 0.01	0.006	0.002	1,928
	0.01 - 0.05	0.016	0.003	522
	0.05 - 0.5	0.012	0.011	683
nonPAR	0 - 0.01	0.483	0.516	562,816
	0.01 - 0.05	0.867	0.934	167,368
	0.05 - 0.5	0.944	0.985	197,858
Overall	0 - 0.01	0.47	0.472	584,653
	0.01 - 0.05	0.839	0.927	174,116
	0.05 - 0.5	0.887	0.982	211,516

Supplementary Table 7. Number of imputed variants on the X chromosome before and after variant filtering.

		Post-imputation, without filtering					Post-imputation, with filtering
**Chr X region**	**Total # variants**	**# variants INFO < 0.4**	**% variants INFO < 0.4**	**# variants MAF $< 1\%$**	**% variants MAF $< 1\%$**	**# variants INFO < 0.4 MAF $< 1\%$**	**# variants INFO ≥ 0.4 MAF $\geq 1\%$ HWE p $> 1e^{-6}$**
PAR1	39,200	36,047	92%	19,418	50%	17,350	1,067
PAR2	3,154	3,128	99%	1,892	60%	1,887	5
nonPAR	957,985	259,220	27%	549,054	57%	256,245	405,820
**Total**	1,000,339	39,175		570,364		275,482	406,892

doi:10.1038/nature24277

Supplementary Table 8. Average imputation quality score (INFO) stratified by minor allele frequency (MAF) for variants on chromosome X, after applying QC filters. INFO was computed using IMPUTE2. Variants tested include SNPs and indels. Variant filters applied: MAF $< 1\%$, INFO < 0.4, HWE $P < 1 \times 10^{-6}$.

MAF	Mean INFO	Median INFO	# variants
0.01-0.5	0.9443	0.985	199,099
0.01-0.05	0.8727	0.935	182,493
0.05-0.1	0.9204	0.976	47,384
0.1-0.2	0.9405	0.984	48,400
0.2-0.3	0.9571	0.989	39,162
0.3-0.4	0.9567	0.988	34,270
0.4-0.5	0.9573	0.987	29,883

Supplementary Table 9. Average imputation quality score (INFO) of autosomal variants stratified by minor allele frequency (MAF), using haploid imputation. Variants tested include SNPs and indels before applying any filtering to the phased variants. INFO was computed using IMPUTE2.

MAF	Mean INFO	Median INFO	# variants
0 - 0.01	0.485	0.52	18,504,053
0.01 - 0.05	0.904	0.964	3,997,251
0.05 - 0.1	0.945	0.989	1,283,309
0.1 - 0.2	0.96	0.993	1,530,730
0.2 - 0.3	0.967	0.994	1,109,200
0.3 - 0.4	0.969	0.994	950,117
0.4 - 0.5	0.97	0.994	884,740
0.05 - 0.5	0.961	0.993	5,758,096

doi:10.1038/nature24277

Supplementary Table 10. Concordance between imputed and phased genotypes (based on haploid imputation) across 450 GTEx samples. The mean and median concordance values (R^2) were computed separately for common (MAF$= 0.05 - 0.50$; A) and low frequency (MAF$= 0.01 - 0.05$; B) variants, and for incremental IMPUTE2 INFO cutoffs starting with INFO > 0.4. INFO: measure of imputation confidence computed by IMPUTE2. MAF: minor allele frequency.

A. $0.05 \leq$ MAF ≤ 0.50

IMPUTE2 INFO	Mean R^2	Median R^2	# Variants
>0.4	0.997	1	5,032,202
>0.5	0.997	1	5,032,202
>0.6	0.997	1	5,032,198
>0.7	0.997	1	5,032,187
>0.8	0.997	1	5,027,302
>0.9	0.997	1	4,949,434

B. $0.01 \leq$ MAF ≤ 0.05

IMPUTE2 INFO	Mean R^2	Median R^2	# Variants
>0.4	0.963	1	4,066,835
>0.5	0.965	1	4,038,503
>0.6	0.969	1	3,953,709
>0.7	0.974	1	3,777,965
>0.8	0.980	1	3,465,497
>0.9	0.985	1	2,920,317

Supplementary Table 11. RSIDs for trans-eVariants flagged as correlated with genotype PCs. These SNPs were flagged based on a threshold of ≥ 99th percentile of correlations observed among random variants (matched for MAF and distance to the nearest TSS), where the maximum correlation is assessed for each variant across all 20 genotype PCs. For all the listed trans-eVariants, the maximum correlation is obtained between the variant's genotype and genotype PC 1.

Variant ID	Maximum correlation
rs28429562	0.785
rs4588372	0.773
rs60413914	0.762
rs8006467	0.746
rs28613059	0.742
rs12114193	0.716
rs17840302	0.712
rs2731983	0.702
rs73112055	0.675
rs2169206	0.662

doi:10.1038/nature24277

Supplementary Table 12. Multi-tissue trans-eQTL using hierarchical FDR control $q = 0.1$ This table shows 23 significant variant-gene associations across all tissues using hierarchical testing procedure.

Variant	Gene Id	Tissue	P-value
rs832723	ENSG00000120437.7	Aorta	2.16×10^{-13}
rs56060157	ENSG00000198105.7	Cortex	6.49×10^{-12}
rs8128148	ENSG00000137824.11	Putamen (basal ganglia)	3.43×10^{-12}
rs11971996	ENSG00000058272.11	Sigmoid Colon	1.55×10^{-12}
rs1441563	ENSG00000162194.8	Esophagus Mucosa	1.53×10^{-12}
rs708993	ENSG00000114455.9	Esophagus Mucosa	1.79×10^{-16}
rs74462116	ENSG00000093144.14	Esophagus Mucosa	9.08×10^{-12}
rs74129340	ENSG00000136104.14	Left Ventricle	6.53×10^{-12}
rs12526847	ENSG00000175701.6	Lung	1.22×10^{-12}
rs73112055	ENSG00000166004.10	Lung	4.07×10^{-12}
rs7033206	ENSG00000132746.10	Not sun exposed skin (suprapubic)	1.42×10^{-12}
rs17116543	ENSG00000106268.11	Sun exposed skin (lower leg)	7.02×10^{-12}
rs6852182	ENSG00000108309.8	Sun exposed skin (lower leg)	3.09×10^{-14}
rs781658	ENSG00000108309.8	Sun exposed skin (lower leg)	3.32×10^{-15}
rs1810232	ENSG00000105185.7	Testis	8.84×10^{-13}
rs2293166	ENSG00000259817.1	Testis	2.49×10^{-11}
rs2745408	ENSG00000138813.5	Testis	1.51×10^{-11}
rs2745409	ENSG00000138813.5	Testis	3.06×10^{-12}
rs36095346	ENSG00000173418.7	Testis	1.11×10^{-13}
rs1867277	ENSG00000112379.8	Thyroid	8.44×10^{-18}
rs1867277	ENSG00000232070.4	Thyroid	7.38×10^{-20}
rs2120262	ENSG00000232070.4	Thyroid	5.36×10^{-13}
rs183791883	ENSG00000179979.7	Whole Blood	6.39×10^{-12}

doi:10.1038/nature24277

Supplementary Table 13. Trans-eVariant and eGene discoveries for restricted approaches in the GTEx data. Each tissue with non-zero values in one or more of the restricted approaches is included as a row with the combined total on the final row; the columns include the number of samples for that tissue, followed by the number of unique trans-eGenes and trans-eVariants identified three restricted runs—restricted to the LD-pruned, cis-eQTL, and trait associated variants—followed by the number of unique trans-eGenes and trans-eVariants identified by any of the four approaches including the genome-wide scan.

		**LD-pruned variants**		**cis-eQTL variants**		**Trait-associated variants**		**All approaches**	
Tissue	No. of samples	eGenes	eVariants	eGenes	eVariants	eGenes	eVariants	eGenes	eVariants
Muscle – Skeletal	361	0	0	3	4	3	2	10	44
Whole Blood	338	1	1	1	1	0	0	3	4
Skin – Sun Exposed (Lower leg)	302	10	12	2	3	2	3	15	30
Adipose – Subcutaneous	298	1	1	4	6	0	0	6	13
Lung	278	2	2	0	0	1	1	3	3
Thyroid	278	6	6	2	1	3	2	24	184
Cells – Transformed fibroblasts	272	0	0	4	5	3	3	6	15
Nerve – Tibial	256	0	0	1	1	0	0	1	1
Esophagus – Mucosa	241	4	4	2	2	0	0	6	14
Esophagus – Muscularis	218	1	1	1	1	1	1	3	3
Artery – Aorta	197	1	1	0	0	0	0	1	1
Skin – Not Sun Exposed (Suprapubic)	196	1	1	0	0	0	0	1	1
Heart – Left Ventricle	190	5	5	0	0	0	0	5	5
Adipose – Visceral (Omentum)	185	0	0	0	0	1	1	1	1
Breast – Mammary Tissue	183	0	0	2	4	0	0	2	4
Colon – Transverse	169	0	0	0	0	0	0	2	10
Heart – Atrial Appendage	159	2	3	0	0	0	0	2	3
Testis	157	5	6	6	6	7	8	41	274
Pancreas	149	0	0	1	2	1	1	3	13
Adrenal Gland	126	0	0	1	1	0	0	2	2
Colon – Sigmoid	124	1	1	0	0	0	0	2	10
Brain – Cerebellum	103	0	0	1	1	0	0	1	1
Brain – Caudate (basal ganglia)	100	7	7	0	0	0	0	7	7
Liver	97	1	1	0	0	0	0	1	1
Brain – Cortex	96	1	1	0	0	0	0	1	1
Brain – Nucleus accumbens (basal ganglia)	93	0	0	2	5	0	0	2	5
Brain – Cerebellar Hemisphere	89	0	0	1	1	0	0	1	1
Pituitary	87	0	0	0	0	1	1	1	1
Brain – Putamen (basal ganglia)	82	1	2	0	0	0	0	3	11
Vagina	79	0	0	0	0	1	1	5	28
Small Intestine – Terminal Ileum	77	0	0	1	1	0	0	1	1
Uterus	70	0	0	0	0	1	1	1	1
Total		47	54	33	41	25	24	157	676

Supplementary Table 14. LD blocks of trans-eVariants that are associated with multiple genes. Four regions including multiple trans-eVariants that have associations with more than one gene are listed. The variants included in each of the regions have linkage disequilibrium $R^2 > 0.5$ based on empirical R^2 values.

Chr.	Location	Associated genes	Tissue	eVariants included	Largest R^2
chr2	210619276-210694720	ENSG00000174567.7, ENSG00000231196.3	Testis	rs10174867, rs10932307, rs10932308, rs10932309, rs10932310, rs11692338, rs12694188, rs13017987, rs13029616, rs13034819, rs1558438, rs17803849, rs1990452, rs2041524, rs2109852, rs2191915, rs2286851, rs2370828, rs2370835, rs731953, rs7574101, rs7588518	0.992
chr5	131785770-131807624	ENSG00000092010.10, ENSG00000178685.9	Skeletal Muscle	rs10059611, rs12659708, rs2057655, rs2522047, rs2522054, rs2522055, rs2522056, rs2522062, rs2522063, rs2522064, rs2706339, rs2706373, rs2706379, rs2706383, rs4504381, rs6866467, rs757105, rs886286	0.548
chr9	115874261-115894240	ENSG00000268580.1, ENSG00000126934.9	Testis	rs2039221, rs62574457, rs7031790, rs7867889	0.841
chr9	100592030-100675976	ENSG00000112379.8, ENSG00000232070.4	Thyroid	rs12004762, rs12006522, rs12343182, rs13302470, rs1443434, rs1465965, rs1561961, rs1867277, rs1867278, rs1867279, rs1867280, rs2120262, rs3021523, rs3021526, rs35324451, rs3758248, rs3808893, rs3824495, rs6478423, rs7023267, rs7024345, rs7027221, rs7031386, rs7034249, rs7034336, rs7034648, rs7037324, rs7038998, rs7046645, rs7048255, rs7849497, rs907577, rs907581, rs925485, rs9299258, rs993501	0.948

Supplementary Table 15. Trans-eQTLs replicated in TwinsUK This table shows three significant variant-gene associations from GTEx trans-eQTL analysis that were also replicated in matched tissues in the TwinsUK dataset.

Variant	Gene Symbol	Tissue	P-value
rs1543438	RP11-725P16.2	Adipose - Subcutaneous	2.06×10^{-7}
rs1543438	RP11-725P16.2	Sun protected skin	2.06×10^{-9}
rs2456491	PDCD5	Lymphoblastoid Cell Lines	9.56×10^{-9}

Supplementary Table 16. GWAS datasets used for co-localization.

Phenotype	Abbreviation	Reference
High density lipid cholesterol levels	HDL	http://www.nature.com/ng/journal/v45/n11/full/ng.2797.html
Low density lipid cholesterol levels	LDL	http://www.nature.com/ng/journal/v45/n11/full/ng.2797.html
Triglycerides levels	TG	http://www.nature.com/ng/journal/v45/n11/full/ng.2797.html
Total cholesterol levels	TC	http://www.nature.com/ng/journal/v45/n11/full/ng.2797.html
Coronary artery disease	CAD	http://www.ncbi.nlm.nih.gov/pubmed/21378990
Body-mass index	BMI	http://www.nature.com/nature/journal/v518/n7538/full/nature14177.html
Waist-to-hip ratio adjusted by BMI	WHRadjBMI	http://www.nature.com/nature/journal/v518/n7538/abs/nature14132.html
Waist-to-hip ratio	WHR	http://www.nature.com/nature/journal/v518/n7538/abs/nature14132.html
Inflammatory bowel disease	IBD	http://www.nature.com/ng/journal/v47/n9/abs/ng.3359.html
Crohn's disease	Crohn's	http://www.nature.com/ng/journal/v47/n9/abs/ng.3359.html
Ulcerative colitis	UC	http://www.nature.com/ng/journal/v47/n9/abs/ng.3359.html
Systemic lupus erythematosus	SLE	http://www.ncbi.nlm.nih.gov/pubmed/26502338
Type 2 diabetes	T2D	http://www.nature.com/ng/journal/v47/n12/abs/ng.3437.html
Fasting glucose levels	FG	http://ukpmc.ac.uk/abstract/MED/20081858
Multiple sclerosis	MS	http://www.ncbi.nlm.nih.gov/pubmed/21833088
Primary biliary cirrhosis	PBC	http://www.ncbi.nlm.nih.gov/pubmed/26394269
Schizophrenia	SCZ	http://www.ncbi.nlm.nih.gov/pubmed/25056061
Heart rate	Heart rate	http://www.ncbi.nlm.nih.gov/pubmed/?term=23583979
Celiac disease	Celiac	http://www.ncbi.nlm.nih.gov/pubmed/20190752
Alzheimer's disease	Alzheimers	http://www.ncbi.nlm.nih.gov/pubmed/?term=24162737
Rheumatoid arthritis	RA	http://www.ncbi.nlm.nih.gov/pubmed/20453842
Bipolar disorder	BIP	http://www.ncbi.nlm.nih.gov/pubmed/21926972
Cross-psychiatric disorder traits	PGCCROSS	http://www.ncbi.nlm.nih.gov/pubmed/23453885

References

1. GTEx Consortium. The Genotype-Tissue Expression (GTEx) pilot analysis: multitissue gene regulation in humans. *Science* **348**, 648–660 (2015).

2. Carithers, L. J. *et al.* A novel approach to high-quality postmortem tissue procurement: The GTEx Project. *Biopreservation and Biobanking* **13**, 311–319 (2015).

3. Purcell, S. *et al.* PLINK: a tool set for whole-genome association and population-based linkage analyses. *American Journal of Humam Genetics* **81**, 559–575 (2007).

4. Howie, B. N., Donnelly, P. & Marchini, J. A flexible and accurate genotype imputation method for the next generation of genome-wide association studies. *PLoS Genetics* **5**, e1000529 (2009).

5. Delaneau, O., Marchini, J. & Zagury, J. F. A linear complexity phasing method for thousands of genomes. *Nature Methods* **9**, 179–181 (2011).

6. 1000 Genomes Project Consortium *et al.* An integrated map of genetic variation from 1,092 human genomes. *Nature* **491**, 56–65 (2012).

7. Marchini, J., Howie, B., Myers, S., McVean, G. & Donnelly, P. A new multipoint method for genome-wide association studies by imputation of genotypes. *Nature Genetics* **39**, 906–913 (2007).

8. Hinch, A. G., Altemose, N., Noor, N., Donnelly, P. & Myers, S. R. Recombination in the Human Pseudoautosomal Region PAR1. *PLoS Genetics* **10** (2014).

9. Price, A. L. *et al.* Principal components analysis corrects for stratification in genome-wide association studies. *Nature Genetics* **38**, 904–909 (2006).

10. Maquat, L. E., Tarn, W. Y. & Isken, O. The pioneer round of translation: features and functions. *Cell* **142**, 368–374 (2010).

11. Nagy, E. & Maquat, L. E. A rule for termination-codon position within intron-containing genes: when nonsense affects RNA abundance. *Trends Biochemical Science* **23**, 198–199 (1998).

12. Fisher, S. *et al.* A scalable, fully automated process for construction of sequence-ready human exome targeted capture libraries. *Genome Biology* **12**, R1 (2011).

13. Trapnell, C., Pachter, L. & Salzberg, S. L. TopHat: discovering splice junctions with RNA-Seq. *Bioinformatics* **25**, 1105–1111 (2009).

14. Wright, F. A. *et al.* Heritability and genomics of gene expression in peripheral blood. *Nature Genetics* **46**, 430–437 (2014).

15. DeLuca, D. S. *et al.* RNA-SeQC: RNA-seq metrics for quality control and process optimization. *Bioinformatics* **28**, 1530–1532 (2012).

16. Stegle, O., Parts, L., Durbin, R. & Winn, J. A Bayesian framework to account for complex non-genetic factors in gene expression levels greatly increases power in eQTL studies. *PLoS Computational Biology* **6**, e1000770 (2010).

17. Westra, H.-J. *et al.* Systematic identification of trans eQTLs as putative drivers of known disease associations. *Nature Genetics* **45**, 1238–1243 (2013).

18. Pickrell, J. K. *et al.* Understanding mechanisms underlying human gene expression variation with RNA sequencing. *Nature* **464**, 768–772 (2010).

19. Ongen, H., Buil, A., Brown, A. A., Dermitzakis, E. T. & Delaneau, O. Fast and efficient QTL mapper for thousands of molecular phenotypes. *Bioinformatics* **32**, 1479–1485 (2016).

20. Storey, J. D. & Tibshirani, R. Statistical significance for genomewide studies. *Proceedings of the National Academy of Sciences of the United States of America* **100**, 9440–9445 (2003).

21. Davis, J. R. *et al.* An efficient multiple-testing adjustment for eQTL studies that accounts for linkage disequilibrium between variants. *American Journal of Human Genetics* **98**, 216–224 (2016).

22. Benjamini, Y. & Hochberg, Y. Controlling the false discovery rate: A practical and powerful approach to multiple testing. *Journal of the Royal Statistical Society. Series B Methodological* **57**, 289–300 (1995).

23. Rao, S. S. *et al.* A 3d map of the human genome at kilobase resolution reveals principles of chromatin looping. *Cell* **159**, 1665–1680 (2014).

24. Langmead, B., Trapnell, C., Pop, M. & Salzberg, S. L. Ultrafast and memory-efficient alignment of short DNA sequences to the human genome. *Genome Biology* **10**, R25 (2009).

25. Peterson, C. B., Bogomolov, M., Benjamini, Y. & Sabatti, C. TreeQTL: hierarchical error control for eQTL findings. *Bioinformatics* **32**, 2556–2558 (2016).

26. Bogomolov, M., Peterson, C. B., Benjamini, Y. & Sabatti, C. Testing hypotheses on a tree: new error rates and controlling strategies. *arXiv preprint arXiv:1705.07529* (2017).

27. Simes, R. J. An improved Bonferroni procedure for multiple tests of significance. *Biometrika* **73**, 751–754 (1986).

28. Storey, J. D. & Tibshirani, R. Statistical significance for genomewide studies. *Proceedings of the National Academy of Sciences* **100**, 9440–9445 (2003).

29. Benjamini, Y. & Bogomolov, M. Selective inference on multiple families of hypotheses. *Journal of the Royal Statistical Society: Series B (Statistical Methodology)* **76**, 297–318 (2014).

30. Hormozdiari, F., Kostem, E., Kang, E. Y., Pasaniuc, B. & Eskin, E. Identifying causal variants at loci with multiple signals of association. *Genetics* **198**, 497–508 (2014).

31. Buil, A. *et al.* Gene-gene and gene-environment interactions detected by transcriptome sequence analysis in twins. *Nature Genetics* **47**, 88–91 (2015).

32. Mohammadi, P., Castel, S. E., Brown, A. A. & Lappalainen, T. Quantifying the regulatory effect size of cis-acting genetic variation using allelic fold change. *Genome Research* **0** (2017).

33. Palowitch, J., Shabalin, A., Zhou, Y., Nobel, A. B. & Wright, F. A. Estimation of interpretable eqtl effect sizes using a log of linear model. *arXiv preprint arXiv:1605.08799* (2016).

34. McDowell, I. C. *et al.* Many long intergenic non-coding RNAs distally regulate mRNA gene expression levels. *bioRxiv* 044719 (2016).

35. Waszak, S. M. *et al.* Population variation and genetic control of modular chromatin architecture in humans. *Cell* **162**, 1039 – 1050 (2015).

36. Lander, E. S. *et al.* Initial sequencing and analysis of the human genome. *Nature* **409**, 860–921 (2001).

37. Marco-Sola, S., Sammeth, M., Guigó, R. & Ribeca, P. The GEM mapper: Fast, accurate and versatile alignment by filtration. *Nature Methods* **9**, 1185–1188 (2012).

38. Harrow, J. *et al.* GENCODE: the reference human genome annotation for The ENCODE Project. *Genome Research* **22**, 1760–1774 (2012).

39. Bates, D., Mächler, M., Bolker, B. & Walker, S. Fitting linear mixed-effects models using lme4. *Journal of Statistical Software* **67**, 1–48 (2015).

40. Lappalainen, T. *et al.* Transcriptome and genome sequencing uncovers functional variation in humans. *Nature* **501**, 506–511 (2013).

41. Giambartolomei, C. *et al.* Bayesian test for colocalisation between pairs of genetic association studies using summary statistics. *PLoS Genetics* **10**, e1004383 (2014).

42. Panousis, N. I., Gutierrez-Arcelus, M., Dermitzakis, E. T. & Lappalainen, T. Allelic mapping bias in RNA-sequencing is not a major confounder in eQTL studies. *Genome Biology* **15**, 467 (2014).

43. Castel, S. E., Levy-Moonshine, A., Mohammadi, P., Banks, E. & Lappalainen, T. Tools and best practices for data processing in allelic expression analysis. *Genome Biology* **16**, 195 (2015).
